# Exploring rotavirus proteome to identify potential B- and T-cell epitope using computational immunoinformatics

**DOI:** 10.1016/j.heliyon.2020.e05760

**Published:** 2020-12-29

**Authors:** Yengkhom Damayanti Devi, Arpita Devi, Hemanga Gogoi, Bondita Dehingia, Robin Doley, Alak Kumar Buragohain, Ch. Shyamsunder Singh, Partha Pratim Borah, C.Durga Rao, Pratima Ray, George M. Varghese, Sachin Kumar, Nima D. Namsa

**Affiliations:** a Department of Molecular Biology and Biotechnology, Tezpur University, Napaam 784 028, Assam, India; b Department of Biotechnology, Royal Global University, Guwahati, India; c Department of Paediatrics, Regional Institute of Medical Sciences, Imphal, India; d Department of Paediatrics and Neonatology, Pratiksha Hospital, Guwahati, India; e School of Liberal Arts and Basic Sciences, SRM University AP, Amaravati, India; f Department of Biotechnology, Jamia Hamdard, Delhi, India; g Department of Infectious Diseases, Christian Medical College, Vellore, India; h Department of Biosciences and Bioengineering, Indian Institute of Technology, Guwahati, India

**Keywords:** Rotavirus, Immune epitope, Structural proteins, Non-structural proteins

## Abstract

Rotavirus is the most common cause of acute gastroenteritis in infants and children worldwide. The functional correlation of B- and T-cells to long-lasting immunity against rotavirus infection in the literature is limited. In this work, a series of computational immuno-informatics approaches were applied and identified 28 linear B-cells, 26 conformational B-cell, 44 T_C_ cell and 40 T_H_ cell binding epitopes for structural and non-structural proteins of rotavirus. Further selection of putative B and T cell epitopes in the multi-epitope vaccine construct was carried out based on immunogenicity, conservancy, allergenicity and the helical content of predicted epitopes. An *in-silico* vaccine constructs was developed using an N-terminal adjuvant (RGD motif) followed by T_C_ and T_H_ cell epitopes and B-cell epitope with an appropriate linker. Multi-threading models of multi-epitope vaccine construct with B- and T-cell epitopes were generated and molecular dynamics simulation was performed to determine the stability of designed vaccine. Codon optimized multi-epitope vaccine antigens was expressed and affinity purified using the *E. coli* expression system. Further the T cell epitope presentation assay using the recombinant multi-epitope constructs and the T cell epitope predicted and identified in this study have not been investigated. Multi-epitope vaccine construct encompassing predicted B- and T-cell epitopes may help to generate long-term immune responses against rotavirus. The computational findings reported in this study may provide information in developing epitope-based vaccine and diagnostic assay for rotavirus-led diarrhea in children's.

## 1 Introduction

Rotavirus is the most common cause of acute gastroenteritis in infants and children worldwide. As per WHO reports of 2013 about 215 000 children under five-years of age die annually due to rotavirus infections mainly in low-income countries [1]. Rotavirus particles naturally excreted in the stools of infected children are transmitted mainly through the fecal-oral route, close-contact and fomites [2]. Rotaviruses are nonenveloped RNA viruses and belongs to the family *Reoviridae*. The mature infectious rotavirus particles is made up of three layers of capsid proteins: outer (proteins VP7 and VP4), middle (protein VP6), and inner (protein VP2). The dsRNA genome of rotavirus encodes for 6 structural proteins and 6 non-structural proteins [3]. Rotavirus infectivity is enhanced by cleavage of VP4 protein into two fragments, VP5∗ (facilitates cell membrane penetration) and VP8∗ (mediates cell attachment) [4]. Rotavirus VP4 and VP7 proteins that are commonly used for serotyping are equally important for vaccine development due to development of neutralizing antibodies to VP7, VP8∗, and VP5∗ during natural rotavirus infection [5]. Rotavirus is further divided into nine serogroups (A-I) based on group specific viral antigen VP6 [6].

Vaccination is considered the most reliable preventive measure to avoid serious consequences of rotaviral gastroenteritis that can even lead to death. Two oral live attenuated rotavirus vaccines (Rotarix (monovalent, GSK Biologicals) and RotaTeq (pentavalent bovine-human reassortant, Merck) were available in the year 2006 for Indian children immunization [7,8]. The effectiveness of Rotarix® and RotaTeq® in high-and middle-income countries were observed high ranging from 85% to 98% [9,10]. However, average efficacies (51%–64%) were found in the low-income Asian and African countries [11,12]. Rotavac™ (Bharat Biotech, Hyderabad, a bovine-human reassortant neonatal 116E strain (G9P [11]) and RotaSiil™ (Serum Institute of India Pvt. Ltd., Pune, a bovine-human reassortant with human G1, G2, G3 and G4 bovine UK G6P [5] backbone) are two Indian-produced live-attenuated oral RV vaccines that are found effective in preventing rotavirus-induced gastroenteritis [13]. The efficacy and effectiveness for Rotavac™ against severe gastroenteritis during the first year of life was 56% [14] and 49% in the subsequent year [15]. RotaSiil is a heat-stable oral vaccine that can retain stability up to 18 months during storage at 40 °C [16]. The vaccine efficacy against severe rotavirus gastroenteritis in an immunized Indian children was 33% [17] and 67% in Niger [18]. The results of an Indian manufactured vaccines showed similar efficacy with the existing commercial vaccines for rotavirus in developing country populations. Live-attenuated strains of RV vaccines have the inherent potential to interchange genetic materials with the circulating strains that might generate a virulent RV strains [19]. Although at a lower rate, live-attenuated oral RV vaccines are associated with the risk of developing intussusception (IS) [20,21] in an age-dependent manner [22,23]. An estimated of about 1–6 cases of IS during the administration of first or second dose for Rotarix® and RotaTeq® per 100,000 immunized children have been reported [20]. Similar rates of IS have been observed for an Indian manufactured vaccines, Rotavac® [24] and RotaSIIL® [25]. Recombinant vaccines could overcome the adverse consequences of live-attenuated vaccines. The common recombinant subunit vaccines are Hepatitis B vaccine which consists of hepatitis B virus surface antigen and recent recombinant vaccine against human papillomaviruses [26]. Novel next generation vaccination involving a rational design of B- and T-cell epitope-based vaccine have made substantial progress in the clinical trials. The recombinant epitope-based malaria vaccine that successfully reached phase-III trials might become the first commercial vaccine for parasitic disease [27].

Immunoinformatic approaches have been used for prediction of an antigenic epitope for vaccine development and high-affinity antibodies for therapeutic and diagnostic applications [28,29]. Some examples of computational immunoinformatic tools that has the potential to help experimental researchers to validate the *in silico* designed epitope-based vaccine includes but not limited to SARS-CoV-2 [30], Zika virus [31], and Nipah virus [32]. *In silico* identified protein regions with high probability of being effective epitopes might help in designing effective experimental assays with improved precision [33]. The present work involves the application of extensive computational immunoinformatic tools to identify potential B and T- cell epitopes to enable us to design a multi-epitope vaccine construct containing predicted antigenic fragments of rotavirus proteins. A tripeptide Arg-Gly-Asp (RGD) cell adhesion motif was added at the N-terminal end of the final vaccine construct to improve the immunogenicity. Allergenicity, antigenicity, epitope conservancy, structural modelling, docking and molecular dynamics simulation of vaccine constructs were carried out to ensure vaccine property of multi-epitope protein. Codon optimized multi-epitope vaccine antigens was expressed, and affinity purified in *E. coli*. The present observations of computational bioinformatics are expected to help researchers to select epitopes for further experimental validations and develop recombinant subunit vaccine against rotavirus.

## 2 Results and discussion

### 2.1 Sequence retrieval and selection of antigenic rotavirus proteins

**Figure 1:**
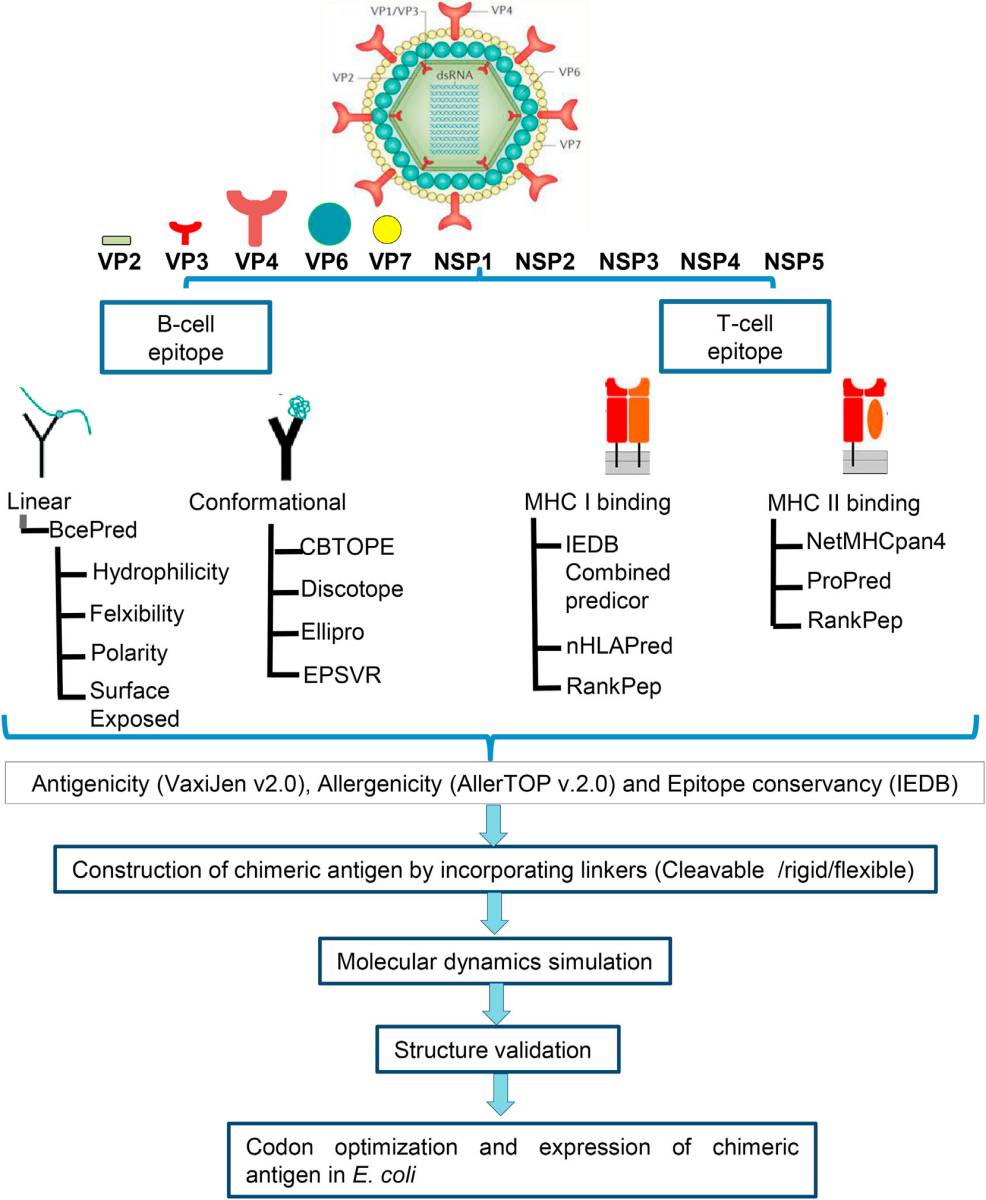
Schematic representation of an *in-silico* strategy followed for prediction of B- and T-cell epitope using proteome of rotavirus.

The prototype simian agent 11 (SA11) was used as group A rotavirus reference strain to retrieve protein sequences in FASTA format due to availability of complete genome sequence (Table S1). Rotavirus pathogenesis is multifactorial and the outcome of disease is determined by both host and viral factors. Genetic analysis of selected virus reassortants identified several proteins of rotaviruses but not limited to VP3, VP4, VP6, VP7, NSP1, NSP2, NSP3, and NSP4 that are involved in virulence. The VP6 protein sequences of Adult diarrheal rotavirus (ADRV) and Cowden strain of porcine were included as group B and group C prototype strains. Among 12 rotavirus proteins, only 9 proteins were predicted as antigenic using VaxiJen v.2.0 with probability of antigenicity scores in the range of 0.4043–0.5734 (Table S1). Rotavirus NSP4 was predicted as non-antigen by VaxiJen which might be due to the limitation of server. NSP4 protein has pleiotropic properties including viral enterotoxin, intracellular role in viral replication and morphogenesis [34]. Recombinant NSP4 protein is known to induce age-dependent diarrhea in suckling mice mimicking a rotavirus disease caused during natural infection [35]. NSP4 and 9 other rotavirus proteins predicted as antigens was analyzed for the identification of B-and T-cell epitopes using well established immunoinformatic prediction methods (Figure 1).

### 2.2 B-cell epitope mapping

**Figure 2:**
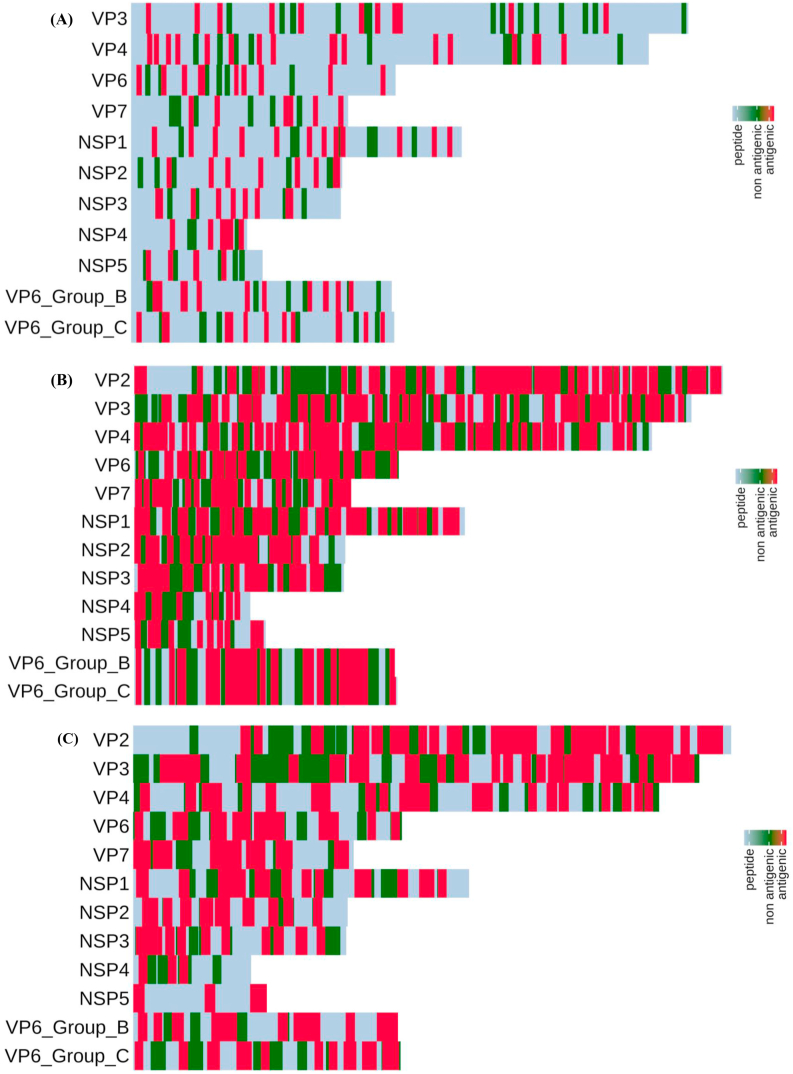
**Summary of rotavirus protein-derived B- and T-cell epitopes**. Heat map showing the distribution of (A) linear (continuous) B-cell epitopes, (B) HLA-class I and (C) II epitopes across the structural and non-structural protein sequences of rotavirus. Strong binding affinity epitopes with <0.5% rank and 2% rank, to HLA class I and class II, respectively, for each HLA molecule are represented here. Red color represents likely antigenic epitopes that were predicted using the methods described in Figure 1.

B cells are primarily involved in the production of antibodies to provide a protective immune defense against the foreign pathogens through the complement activation, antibody-mediated cytotoxicity, and Fc-mediated endocytosis. Additionally, B cells can exert antibody-independent functions like antigen presentation, cytokine secretion, and immunoregulation [36]. The possible presence of linear B-cell epitopes in structural (VP2, VP3, VP4, VP6, VP7) and non-structural (NSP1, NSP2, NSP3, NSP4, NSP5) proteins were predicted by Bcepred using parameters such as antigenicity, surface accessibility, turns, flexibility and hydrophilicity. Bcepred prediction method uses a dataset of 1029 experimentally proved continuous B cell epitopes and 1029 non-epitopes covering a proteins derived from viruses, bacteria, protozoa and fungi. Therefore, Bcepred server is a reliable method used to predict the linear B-cell epitopes with an accuracy of 58.70% based on amino acid properties namely hydrophilicity, flexibility, polarity and exposed surface [37]. A total of 31 linear B-cell epitopes (9–15-mer length) were predicted for all proteins including 3 and 4 epitopes for group B and group C VP6 protein, respectively (Table 1). We have developed a heat map to show the distribution of predicted linear B-cell epitopes across structural and nonstructural proteins of rotavirus (Figure 2A). We have selected 14 continuous B-cell epitopes with 2 epitopes each from VP6 group B and group C for inclusion in the vaccine construct based on epitope conservancy and agadir score of predicted epitopes. A maximum of 6 linear B-cell epitopes were predicted for VP4, 1 epitope was predicted for NSP5 and no epitope was predicted for NSP1 (Table S2a). A conformational B-cell epitope is made of discontinuous stretches of amino acid residues that are tightly held together in tertiary conformation and as over 90% of epitopes are conformational epitopes [38]. Rotaviral proteins lacking crystallographic structures were modeled using multiple- threading approach server-I-TASSER. The c-score of best modeled structures were 0.010, -0.84, -1.41, -1.33, -1.71, -1.17 and -2.83 for VP2, VP3, VP4, VP7, NSP3, NSP4 and NSP5, respectively (Figure S1). The best selected modeled structures were energy minimized and validated structures were used for prediction of conformational B-cell epitopes using four different independent tools to achieve maximum accuracy of computed epitope mapping; CBTOPE, DiscoTope 2.0, Ellipro and EPSVR (Table S2b). The epitopes predicted by at least two different tools and found as antigenic, non-allergenic with agadir score and good conservancy across antigens have been selected as potential discontinuous B-cell epitope. Maximum 4 discontinuous 9 to 20-mer epitopes were predicted for VP4, while only 1 epitope each was predicted for NSP2, and VP4 (Table 2). The localization of selected linear B-cell epitope in their native rotavirus protein structure was carried out using structural superimposition (Figure S2). Agadir algorithm analyzes the stability of isolated α-helices and the alpha-helical tendency of the peptide in solution [39]. We found the epitope of rotavirus proteins VP3 (aa238-249), VP7 (aa168–184), NSP2 (aa298-312), NSP3 (aa108-120), and NSP5 (aa170-183) that are predicted to function as both linear and conformational B-cell epitopes (Table S2a and S2b).

**Table 1:** Rotavirus proteins, total number of epitopes predicted and the immunogenicity/antigenicity/allergenicity as obtained from immune epitope database.

Sl No.	RV protein	B-/T-cell epitope	Types	Total no. of epitopes	Selected Epitope for vaccine construct	Length	Immunogenicity	Antigenicity	Allergenicity	Agadir Score	Conservancy
1	VP2	B-cell epitope	Linear	5	189-AVENKNSRDAGK-200	12	-	-	-	0.33	98.82%
			Confo	4	K339,E340,L341,V342,S343,T344,E345,A346,Q347,I348,Q349,K350,M351	13	-	-	-	0.41	97.65%
		T-cell epitope	MHC I	14	544-QLVDLTRLL-552	9	0.09866	-	Non-allergen	-	95.88%
			MHC II	10	534-GILLLSNRLGQLV-546	13	-	0.7962	Non-allergen	-	97.65%
2	VP3	B-cell epitope	Linear	3	238-TIKLKQERWLGK-249	12	-	-	-	0.36	46.08%
			Confo	3	R176,M177,T178,T179,S180,L181,P182,I183,A184,R185,L186,S187,N188,R189,V190,F191,R192	17	-	-	-	0.53	98.16%
		T-cell epitope	MHC I	4	72-LFTLIRCNF-80	9	0.13048	-	Non-allergen	-	68.66%
			MHC II	5	612-HVYNALIYYRYNY-624	13	-	0.6634	Non-allergen	-	97.24%
3	VP4	B-cell epitope	Linear	6	241-RDVIHYRAQANED-253	13	-	-	-	0.29	4.00%
					208-IPRSEESKCTEYI-220	14	-	-	-	0.41	4.67%
					262-WKEMQYNRDI-271	10	-	-	-	0.48	97.33%
					657-PDIVTEASEKF-667	11	-	-	-	0.4	76.67%
			Confo	1	T413,Q414,F415,T416,D417,F418,V419,S420,L421,N422,S423,L424	12	-	-	-	0.27	93.33%
		T-cell epitope	MHC I	3	288-GYKWSEISF-296	9	0.1017	-	Non-allergen	-	25.93%
			MHC II	3	416-TDFVSLNSLRFRF-428	13	-	1.3966	Non-allergen	-	94.67%
4	VP6	B-cell epitope	Linear	5	9-KTLKDARDKIVEG-21	13	-	-	-	0.63	88.52%
					139-WNLQNRRQRTG-149	11	-	-	-	0.69	98.36%
					373-NYSPSREDNLQR-384	12	-	-	-	0.3	96.72%
			Confo	3	Y24,S25,N26,V27,S28,D29,L30,I31,Q32,Q33,F34,N35,Q36	13	-	-	-	0.57	90.16%
					D74,A75,N76,Y77,V78,E79,T80,A81,R82,N83,T84,I85,D86,Y87	14	-	-	-	0.58	44.26%
		T-cell epitope	MHC I	4	226-LPDAERFSF-234	9	0.19493	-	Non-allergen	-	95.08%
			MHC II	4	284-NFDTIRLSFQLMR-296	13	-	0.5044	Non-allergen	-	91.87%
5	VP7	B-cell epitope	Linear	2	308-QVMSKRSRSLNSA-320	13	-	-	-	0.28	73.88%
			Confo	4	R286,I287,N288,W289,K290,K291,W292,W293,Q294,V295	10	-	-	-	0.61	26.87%
					D169,I170,T171,L172,Y173,Y174,Y175,Q176,Q177,T178,D179,E180,A181,N182,K183,W184	16	-	-	-	0.32	29.10%
		T-cell epitope	MHC I	5	15-SIILLNYIL-23	9	0.08979	-	Non-allergen	-	67.91%
			MHC II	2	13-LISIILLNYILKS-25	13	-	0.5661	Non-allergen	-	57.46%
6	NSP2	B-cell epitope	Linear	2	267-QNWYAFTSSMKQGNT-281	15	-	-	-	0.32	72.34%
			Confo	1	N298,P299,F300,K301,G302,L303,S304,T305,D306,R307,K308,M309,D310,E311,V312,S313	16	-	-	-	0.39	89.36%
		T-cell epitope	MHC I	3	9-YPHLENDSY-18	9	0.01281	-	Allergen	-	94.68%
			MHC II	3	46-SIIYGIAPPPQFK-58	13	-	0.6568	Non-allergen	-	41.49%
7	NSP3	B-cell epitope	Linear	3	108-LSSKGIDQKMRVL-120	13	-	-	-	0.48	96.74%
			Confo	3	K77,F78,G79,S80,A81,I82,R83,N84,R85,N86	10	-	-	-	0.36	45.65%
		T-cell epitope	MHC I	3	58-GVKNNLIGK-66	9	0.03887	-	Non-allergen	-	28.26%
			MHC II	2	101-NKLRMMLSSKGID-113	13	-	0.8777	Non-allergen	-	22.83%
8	NSP4	B-cell epitope	Linear	4	117-TTREIEQVELLK-128	13	-	-	-	0.48	96.74%
			Confo	2	I51,P52,T53,M54,K55,I56,A57,L58,K59	9	-	-	-	0.36	91.74%
		T-cell epitope	MHC I	2	36-IASVLTVLF-44	9	0.04194		Non-allergen		91.07%
			MHC II	3	29-GMAYFPYIASVLT-41	13		0.7181	Non-allergen		92.86%
9	NSP5	B-cell epitope	Linear	1	170-KCKNCKYKKKYFAL-183	14	-	-	-	0.55	74.47%
			Confo	3	A66,S67,N68,D69,P70,L71,T72,S73,F74,S75,I76,R77,S78,N79,A80,V81,K82,T83,N84,A85	20	-	-	-	0.5	89.36%
		T-cell epitope	MHC I	1	2-SLSIDVTSL-10	9	0.07678		Allergen		92.55%
			MHC II	4	176-YKKKYFALRMRMK-188	13		1.5785	Non-allergen		47.87%
10	VP6 Group B	B-cell epitope	Linear	3	74-ISTDDYDDMRSGI-86	13	-	-	-	0.28	76.19%
					197-GMDSEHRFTVELKTR-211	15	-	-	-	0.57	34.38%
			Confo	1	E154,N155,P156,L157,Y158,A159,D160,I161,I162,E163,Q164,I165,V166,H167,R168	15	-	-	-	0.59	35.71%
		T-cell epitope	MHC I	3	89-ILDVLAAAI-97	9	0.13065	-	Non-allergen	-	64.29%
			MHC II	1	315-AISFMFETRRTFT-327	13	-	1.2887	Non-allergen	-	50.00%
11	VP6 Group C	B-cell epitope	Linear	4	93-EAVCDDEIVREA-104	12	-	-	-	0.48	86.96%
					143-SRRENPVYEYKNPM-156	14	-	-	-	0.3	60.87%
			Confo	1	F364,P365,W366,E367,Q368,T369,L370,S371,N372,Y373,T374,V375,A376,Q377,E378	15	-	-	-	0.32	59.57%
		T-cell epitope	MHC I	2	325-ILDATTESV-233	9	0.10875	-	Non-allergen	-	69.57%
			MHC II	3	381-LERLLLVASVKRM-393	13	-	0.415	Non-allergen	-	44.68%

**Table 2:** **Predicted B- and T-cell epitopes obtained from the immune epitope database**. The amino acid sequence of selected epitopes used for design of final multi-subunit chimeric antigen constructs.

Multi-epitope antigen construct		RV Protein	Linear B-cell epitope	Conformational B-cell epitope	CTL epitope	HTL epitope
VP6A/B/C (Construct 1)		VP6 GroupA	9-KTLKDARDKIVEG-21, 139-WNLQNRRQRTG-149, 373-NYSPSREDNLQR-384	Y24,S25,N26,V27,S28,D29,L30,I31,Q32,Q33,F34,N35,Q36	226-LPDAERFSF-234	284-NFDTIRLSFQLMR-296
				D74,A75,N76,Y77,V78,E79,T80,A81,R82,N83,T84,I85,D86,Y87		
		VP6 Group B	74-ISTDDYDDMRSGI-86, 197-GMDSEHRFTVELKTR-211	E154,N155,P156,L157,Y158,A159,D160,I161,I162,E163,Q164,I165,V166,H167,R168	89-TINAPIISL-97	315-AISFMFETRRTFT-327
		VP6 Group C	93-TVSDLKKKV-104, 143-EAVCDDEIVREA-156	F364,P365,W366,E367,Q368,T369,L370,S371,N372,Y373,T374,V375,A376,Q377,E378	325-ILDATTESV-334	381-LERLLLVASVKRM-393
VP4/6/7 (Construct 2)	VP4/A (Construct 8)	VP4	241-RDVIHYRAQANED-253, 208-IPRSEESKCTEYI-220, 262-WKEMQYNRDI-271, 657-PDIVTEASEKF-667	T413,Q414,F415,T416,D417,F418,V419,S420,L421,N422,S423,L424	288-GYKWSEISF-296	416-TDFVSLNSLRFRF-428
	VP6/A (Construct 9)	VP6	9-KTLKDARDKIVEG-21, 373-NYSPSREDNLQR-384	Y24,S25,N26,V27,S28,D29,L30,I31,Q32,Q33,F34,N35,Q36	226-LPDAERFSF-234	284-NFDTIRLSFQLMR-296
				D74,A75,N76,Y77,V78,E79,T80,A81,R82,N83,T84,I85,D86,Y87		
	VP7/A (Construct 10)	VP7	308-QVMSKRSRSLNSA-320	D169,I170,T171,L172,Y173,Y174,Y175,Q176,Q177,T178,D179,E180,A181,N182,K183,W184	15-SIILLNYIL-23	13-LISIILLNYILKS-25
VP2/3/4/6/7-NSP2/3/4/5 (Construct 5)	VP2/3/4/6/7 (Construct 3)	VP2	189-AVENKNSRDAGK-200	K339,E340,L341,V342,S343,T344,E345,A346,Q347,I348,Q349,K350,M351	544-QLVDLTRLL-552	534-GILLLSNRLGQLV-546
		VP3	238-TIKLKQERWLGK-249	R176,M177,T178,T179,S180,L181,P182,I183,A184,R185,L186,S187,N188,R189,V190,F191,R192	72-LFTLIRCNF-80	612-HVYNALIYYRYNY-624
		VP4	657-PDIVTEASEKF-667	T413,Q414,F415,T416,D417,F418,V419,S420,L421,N422,S423,L424	288-GYKWSEISF-296	416-TDFVSLNSLRFRF-428
		VP6	373-NYSPSREDNLQR-384	Y24,S25,N26,V27,S28,D29,L30,I31,Q32,Q33,F34,N35,Q36	226-LPDAERFSF-234	284-NFDTIRLSFQLMR-296
		VP7	308-QVMSKRSRSLNSA-320	D169,I170,T171,L172,Y173,Y174,Y175,Q176,Q177,T178,D179,E180,A181,N182,K183,W184	15-SIILLNYIL-23	13-LISIILLNYILKS-25
	NSP2/3/4/5 Construct 4)	NSP2	267-QNWYAFTSSMKQGNT-281	N298,P299,F300,K301,G302,L303,S304,T305,D306,R307,K308,M309,D310,E311,V312,S313	9-YPHLENDSY-18	46-SIIYGIAPPPQFK-58
		NSP3	108-LSSKGIDQKMRVL-120	K77,F78,G79,S80,A81,I82,R83,N84,R85,N86	58-GVKNNLIGK-66	101-NKLRMMLSSKGID-113
		NSP4	117-TTREIEQVELLK-128	I51,P52,T53,M54,K55,I56,A57,L58,K59	36-IASVLTVLF-44	29-GMAYFPYIASVLT-41
		NSP5	170-KCKNCKYKKKYFAL-183	A66,S67,N68,D69,P70,L71,T72,S73,F74,S75,I76,R77,S78,N79,A80,V81,K82,T83,N84,A85	2-SLSIDVTSL-10	176-YKKKYFALRMRMK-188
VP6A/B/C–B (Construct 6)		VP6 GroupA	9-KTLKDARDKIVEG-21, 139-WNLQNRRQRTG-149, 373-NYSPSREDNLQR-384	Y24,S25,N26,V27,S28,D29,L30,I31,Q32,Q33,F34,N35,Q36	-	-
				D74,A75,N76,Y77,V78,E79,T80,A81,R82,N83,T84,I85,D86,Y87		
		VP6 Group B	74-ISTDDYDDMRSGI-86, 197-GMDSEHRFTVELKTR-211	E154,N155,P156,L157,Y158,A159,D160,I161,I162,E163,Q164,I165,V166,H167,R168	-	-
		VP6 Group C	93-TVSDLKKKV-104, 143-EAVCDDEIVREA-156	F364,P365,W366,E367,Q368,T369,L370,S371,N372,Y373,T374,V375,A376,Q377,E378	-	-
VP4/6/7-B (Construct 7)		VP4	241-RDVIHYRAQANED-253, 208-IPRSEESKCTEYI-220, 262-WKEMQYNRDI-271, 657-PDIVTEASEKF-667	T413,Q414,F415,T416,D417,F418,V419,S420,L421,N422,S423,L424	-	-
		VP6	9-KTLKDARDKIVEG-21, 139-WNLQNRRQRTG-149, 373-NYSPSREDNLQR-384	Y24,S25,N26,V27,S28,D29,L30,I31,Q32,Q33,F34,N35,Q36	-	-
				D74,A75,N76,Y77,V78,E79,T80,A81,R82,N83,T84,I85,D86,Y87		
		VP7	308-QVMSKRSRSLNSA-320	D169,I170,T171,L172,Y173,Y174,Y175,Q176,Q177,T178,D179,E180,A181,N182,K183,W184	-	-

### 2.3 Computational mapping of T-cell epitopes

In a given population some representative alleles called supertypes are found more frequently than others and have commonly shared binding specificity and these are of empirical use for epitope based-vaccine development [40]. 27 alleles (Table S3a) of major Human Leukocyte Antigen class I (HLA-I) supertypes are known to have more than 97% population coverage (African Americans, Caucasians, Hispanics, Asians, North American Natives) [41,42]. Similarly, for HLA-II, 27 alleles (Table S3b) have been shown to provide more than 99% population coverage [43,44].

Cytotoxic T lymphocyte (CTL) belongs to the CD8+ subset of T cells that are associated with killing of cells-infected with intracellular virus, bacterial or protozoal parasites. CD8+ T cells bind epitopes that are presented by the MHC class I molecule while the CD4+ T cells recognize epitopes that are presented by MHC class I molecule. T cells are required for both cell- and antibody-mediated immune protection system. A total of 39 CTL epitopes (9-mer) were predicted for rotavirus proteome using three different tools namely IEDB Proteasomal cleavage/TAP transport/MHC class I combined predictor, nHLAPred and RankPep (Table 1). Similarly, a heat map was generated to indicate the distribution of predicted HLA-class I (Figure 2B) and II epitopes (Figure 2C) of structural and non-structural proteins of rotavirus. The highly conserved VP6 capsid protein sequences from group B and C rotaviruses were similarly analyzed and predicted 3 and 2 CTL epitopes, respectively (Table 2 and Table S4a). We used NetMHCpan 3.1, ProPred and RankPep tools for prediction of HTL epitopes based on high affinity score. We have predicted and found total of 36 HTL epitopes (13-mer) for all proteins analyzed including one and three epitopes for VP6 of group B and C rotaviruses, respectively (Table 2 and Table S4b). Herein we reported prediction of CTL and HTL epitopes of rotavirus proteins using a total of 27 alleles of major HLA-I and HLA-II supertypes. Immunogenicity of CTL epitopes was predicted using IEDB method and epitopes that were assigned a higher score were selected indicating a greater probability to induce an immune response. Similarly, using IEDB tool we have predicted the immunogenicity of MHC class II epitopes and the results generated peptides of 15-mer amino acid residues with immunogenicity score ranging from 73-99 indicating non-immunogenic peptides (Table S4b). The observed poor predicted immunogenicity of CD4 epitopes could be due to the availability of only seven number of HLA alleles in the database tool [45,46]. The probable cross-reactive allergenic peptides were predicted by online server AllerTOP v2.0. IEDB tool was used to analyze epitope conservancy with sequence identity threshold less than or equal to 100% and avoiding duplicated protein sequences. We have applied four criteria such as immunogenicity, non-allergenicity, conservancy and the same epitopes is being predicted by two of the three methods used to qualify the selection of predicted CTL and HTL epitopes for docking with MHC molecules (Table S4a and S4b).

### 2.4 Docking of predicted T-cell epitope

The structure of peptide was generated by PEP-FOLD 2.0 and capping was performed. The PDB files of MHC I and MHC II molecules were retrieved from the Protein Data Bank and 27 HLA supertypes were used in the prediction of epitopes (Table S5a and S5b). MHC I alleles lacking crystal structure was modelled using I- TASSER. Some of the MHC II alleles were used as reference crystallographic structures for docking with predicted T-cell epitopes. All MHC structures were energy minimized and MHC-peptide docking simulation was performed using ClusPro v.2.0 [47]. Interaction energy was analyzed by prodigy. CTL and HTL epitopes predicted as immunogenic/antigenic, non-allergenic and conserved across the antigens have been selected for designing multi-epitope vaccine based on docking score or free energy (Table S5a and S5b). We have selected the docking models of MHC-I/II and T-cell epitope complexes having the lowest binding energies. Conserved peptides with their interaction energies for structural and non-structural proteins in kcal mol^−1^ are given in Table S5a and S5b. Binding studies have shown that nonameric peptide is the most compatible length and binds MHC I molecules with the closed-ended peptide-binding cleft than peptides longer or shorter than nonameric peptide [48]. Anchor residues are generally hydrophobic in nature and found one at carboxyl terminus and second and third in amino-terminal end of the peptide (Table S4). MHC II binding peptides has specific motif with a central core of 13 amino acid residues. Internal sequence stretches of 7–10 residues form the contact points with an N-terminal aromatic or hydrophobic residue, three hydrophobic residues at the centre and carboxyl end of the peptide (Table S4b). This criterion was considered for the selection of final potent T-cell epitopes.

### 2.5 Designing of multi-epitope subunit vaccine

**Figure 3:**
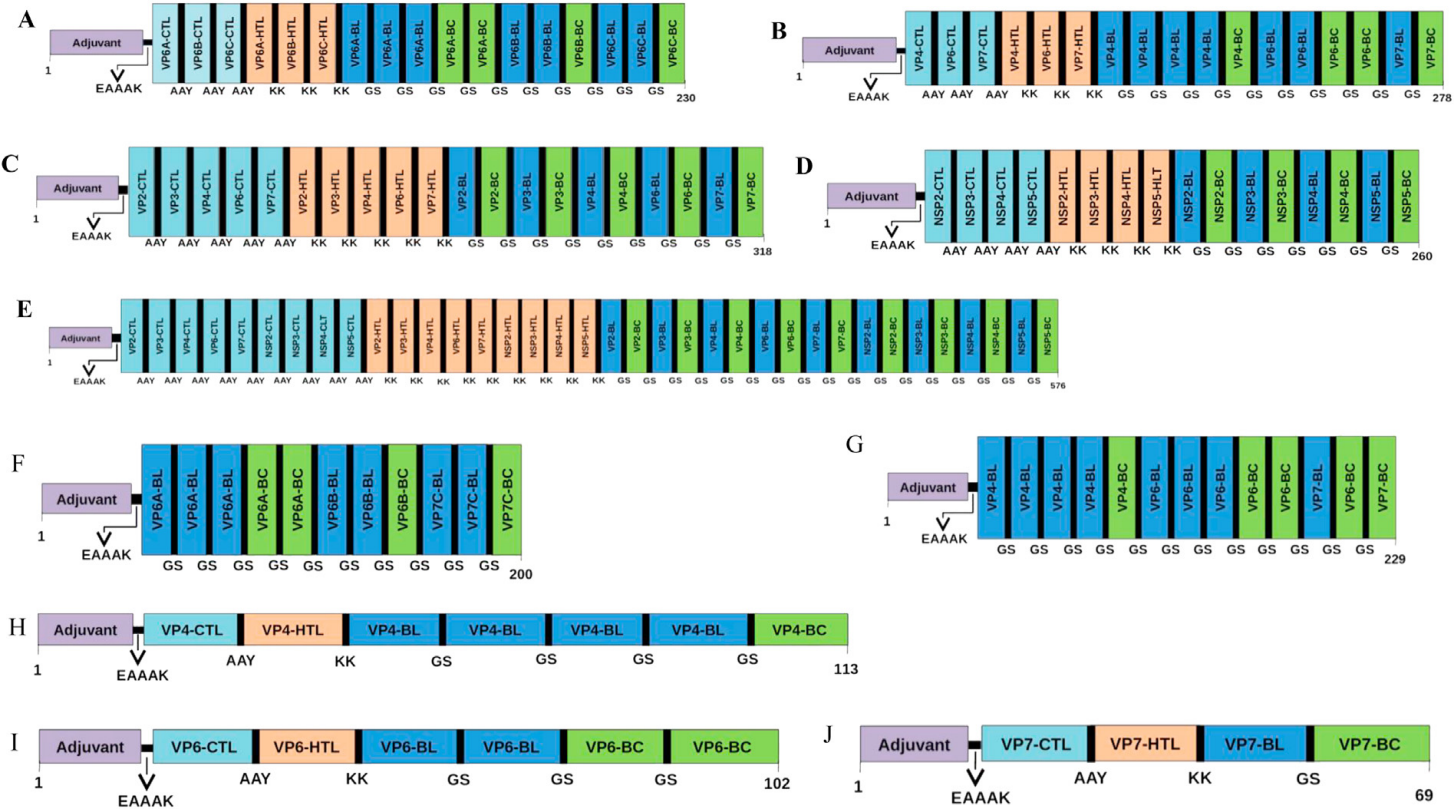
**Schematic diagram of multi-epitope chimeric constructs**. The multi-epitope constructs sequence consisting adjuvant followed by T- and B-cell epitope. Adjuvant and CTL epitope has been joined by EAAAK linker, whereas the AAY, KK and GGGGS linkers were used to join the CTL, HTL and linear/conformational B-cell epitopes, respectively. A. Construct 1 (VP6A/B/C), B. Construct 2 (VP6/4/7), C. Construct 3 (VP2/3/4/6/7), D. Construct 4 (NSP2/3/4/5) and E. Construct 5 (VP2/3/4/6/7-NSP2/3/4/5), F. Construct 6 (VP6A/B/C–B), G. Construct 7 (VP4/6/7-B), H. Construct 8 (VP4/A), I. Construct 9 (VP6/A) and J. Construct 10 (VP7/A); (BL- Linear B-cell epitope, BC- Conformational B-cell epitope). A/B/C: VP6 sequence of group A, B and C rotaviruses; A: group A rotavirus; B: B-cell epitopes (Both linear and conformational).

A total of 10 multi-epitope vaccine constructs comprising of 69 amino acids (aa) through 576 aa consisting of 11 CTL, 11 HTL, 18 linear and 14 conformational B-cell epitopes have been described (Table 1 and Table 2). These predicted epitopes were derived from 5 structural (VP2, VP3, VP4, VP6 and VP7) and 4 non-structural proteins (NSP2, NSP3, NSP4 and NSP5). Linear B-cell epitopes were selected and included in the vaccine constructs based on (i) Agadir score (the helical content of peptide), (ii) conservancy (Table S2a), (iii) surface localization of epitopes on the native protein of rotavirus (Figure S2). Conformational B cell epitopes were selected based on prediction of the same epitope by (i) two prediction tools used, (ii) Agadir score and (iii) conservancy (Table S2b). Similarly, T cell epitopes were included in the multi-subunit vaccine constructs based on prediction of the same epitope by (i) three prediction tools used, (ii) antigenicity/immunogenicity, (iii) non-allergenicity, (iv) conservancy (Table S4a & S4b), and best docking score (Table S5a & S5b). Each epitope in the vaccine construct was occupied by the appropriate linkers, adjuvant and CTL epitopes were combined by EAAAK rigid linker, intra-CTL and intra-HTL epitopes joint by AAY and KK cleavable linker, respectively, and B- cell epitopes were linked together by GGGGS flexible linker [30,31,32,49]. Poly-Gly-rich flexible linkers are well characterized and generally do not affect the folding and function of fusion proteins [50]. Finally, vaccine construct was made containing N-terminal integrin binding motif (RGD) as adjuvant, CTL, HTL and B-cell epitopes (Figure 3). Rotavirus entry into the cell involves a multi-step process with sialic acid and integrins as viral receptors. The arginine-glycine-aspartate (RGD) motif has been shown to enhance immunogenicity and adjuvanicity in peptide antigens [51]. Since integrins are used as one of the receptors by rotavirus, RGD motif was selected as biological adjuvant to improve the immunogenicity of vaccine constructs. The rationale for a non-live subunit RV vaccine has no competition of uptake with enteric viruses in the gut, live-attenuated oral RV vaccines have lower rate of efficacies in developing countries and genetic background of the population is not critical (e.g. secretors/non-secretors) [11,12]. We have designed a total of 10 possible multi-subunit vaccine constructs using the predicted B and T cell epitopes of RV proteins. Construct 1 (VP6A/B/C) has a combination of B and T cell epitope predicted using the VP6 protein of group A, B and C rotaviruses, Construct 2 (VP4/6/7) has B and T cell epitope of VP4, VP6 and VP7 proteins, Construct 3 (VP2/3/4/6/7) has B and T cell epitope of VP2, VP3, VP4, VP6 and VP7 proteins, Construct 4 (NSP2/3/4/5) has B and T cell epitope of NSP2, NSP3, NSP4 and NSP5 proteins, Construct 5 (VP2/3/4/6/7-NSP2/3/4/5) has B and T cell epitope of VP2, VP3, VP4, VP6, VP7, NSP2, NSP3, NSP4, and NSP5 proteins, Construct 6 (VP6A/B/C–B) has B-cell epitope predicted using the VP6 protein of group A, B and C rotaviruses, Construct 7 (VP4/6/7-B) has B cell epitope of VP4, VP6 and VP7 proteins, Construct 8 (VP4/A) has B and T cell epitope of VP4 protein of group A rotavirus, Construct 9 (VP6/A) has B and T cell epitope of VP6 protein of group A rotavirus, and Construct 10 (VP7/A) has B and T cell epitope of VP7 protein of group A rotavirus (Table 1 and Table 2). Some of the constructs were designed with predicted B-cell epitope with a long-term goal to express chimeric antigen to possibly develop rapid detection of rotavirus antigen in stool specimens. Intestinal mucosal immunity mediated mainly by IgA antibodies is often associated with protective immunity during rotavirus reinfection. Rotavirus particle is made up of three layers of capsid proteins surrounding a genome of 11 segments of double-stranded RNA. The outer capsid layer is composed of VP4 and VP7 proteins that are mainly responsible for viral attachment and entry are often targeted for protective neutralizing antibodies during rotavirus infections [5,52]. However, a non-neutralizing IgA monoclonal antibody directed against the VP6 protein have been shown to protect mice from rotavirus infection [53]. Studies on several non-replicating RV vaccines are being performed and assessed in various animal models. The expressed truncated VP6 and VP8 protein sub-units, bivalent vaccine (NSP4 & VP6) and ‘‘virus-like particles” (VP2, VP4, VP6, and VP7) are being investigated as potential vaccine for rotavirus [54]. The results of preclinical studies have shown that majority of these potential vaccine candidates for rotavirus induces a strong immune responses and provides a protection against oral challenge with rotavirus strains in mice model [55,56,57]. VP6-specific polyclonal IgA inhibits RV replication at the transcription level by blocking channels on RV particles and preventing RV mRNA release [58]. The recent findings of an efficient intracellular neutralization mediated mainly by VP6-specific IgG and subsequent protection of mice against the challenge of rotavirus might have implications in developing next generation vaccine for rotavirus [59].

### 2.6 Allergenicity, antigenicity and physicochemical parameters of the vaccine constructs

All vaccine constructs were predicted as non-allergenic by Allertop v.2. Construct 1 was predicted as non-antigenic by Vexijen v.2.0 with a score (3.888) close to default threshold value of 4.0. Of 10 vaccine constructs, the antigenicity of construct 4 (NSP2/3/4/5), construct 5 (VP2/3/4/6/7- NSP2/3/4/5) and construct 8 (VP4/A) were predicted to be 0.7059, 0.6263, 0.7943, respectively, using the VaxiJen server indicating the probable antigenic properties of vaccine constructs (Table 3). Various physicochemical parameters of vaccine constructs were analyzed by ProtParam. The final vaccine constructs were found moderately thermostable based on the aliphatic index scores (Table 3). We found negative value of gravy scores suggesting the likelihood of multi-epitope vaccine being globular and hydrophilic in nature.

**Table 3:** Physico-chemical parameter of final multi-epitope constructs. Number of residues, theoretical pI, molecular weight, aliphatic index, and grand average of hydrophobicity (GRAVY) by ProtParam.

Multi-epitope antigen	No. of residues	Isoelectric Point	Mol Wt. in KDa	Aliphatic Index	GRAVY Score	Secondary structure by PSIPRED	Allergenicity by Allertop v.2.0	Antigenicity by Vexijen v2.0 (T = 0.4)
VP6A/B/C (Construct 1)	230	5.24	24.77	70.04	-0.532	52.2% Helix, 3.0% Sheet and 44.8% Coil	Non-allergen	Non-antigen (0.3888)
VP4/6/7 (Construct 2)	278	8.91	30.10	66.01	-0.613	10.43% Helix, 9.71% Sheet and 79.86% Coil	Non-allergen	Antigen (0.5901)
VP2/3/4/6/7 (Construct 3)	318	9.96	34.87	81.32	-0.375	36.2% Helix,11.0% Sheet and 52.8% Coil	Non-allergen	Antigen (0.5537)
NSP2/3/4/5 (Construct 4)	260	10.11	27.63	66.88	-0.428	25.39% Helix, 11.92% Sheet and 62.69% Coil	Non-allergen	Antigen (0.7059)
VP2/3/4/6/7-NSP2/3/4/5 (Construct 5)	576	10.04	62.05	74.91	-0.369	34.0% Helix, 3.7% Sheet and 62.3% Coil	Non-allergen	Antigen (0.6263)
VP6A/B/C–B (Construct 6)	200	4.83	20.64	52.65	-0.872	28.5% Helix, 4.0% Sheet and 67.5% Coil	Non-allergen	Antigen (0.5319)
VP4/6/7-B (Construct 7)	229	6.83	24.04	45.59	-0.976	19.2% Helix, 4.8% Sheet and 76.0% Coil	Non-allergen	Antigen (0.5374)
VP4/A (Construct 8)	113	6.80	12.31	50.09	-0.777	11.5% Helix, 32.7% Sheet and 55.8% Coil	Non-allergen	Antigen (0.7943)
VP6/A (Construct 9)	102	6.45	11.09	55.59	-0.884	40.2% Helix, 2% Sheet and 57.8% Coil	Non-allergen	Antigen (0.4670)
VP7/A (Construct 10)	69	9.52	7.7	110.43	-0.123	59.4% Helix, 11.6% Sheet and 29% Coil	Non-allergen	Antigen (0.4165)

### 2.7 Prediction of secondary and tertiary structure

**Figure 4:**
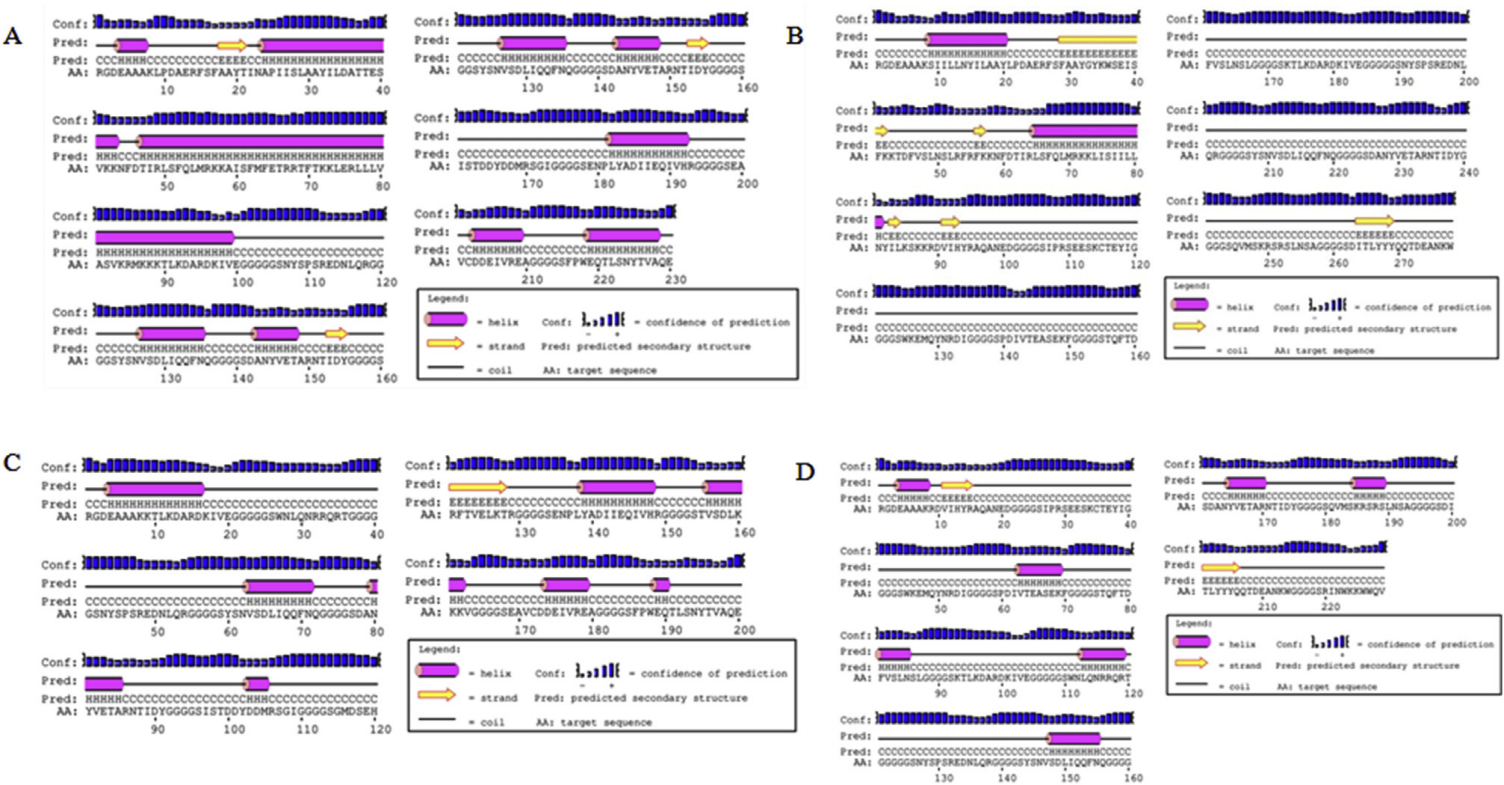
**Graphical representation of secondary structure obtained for the multi-epitope constructs using PSIPRED server**. A. Construct 1, 52.2% helix, 3.0% sheet and 44.8% coil, B. Construct 2, 10.43% helix, 9.71% sheet and 79.86% coil, C. Construct 6, 28.5% helix, 4.0% sheet and 67.5% coil and D. Construct 7, 19.2% helix, 4.8% sheet and 76.0% coil.

**Figure 5:**
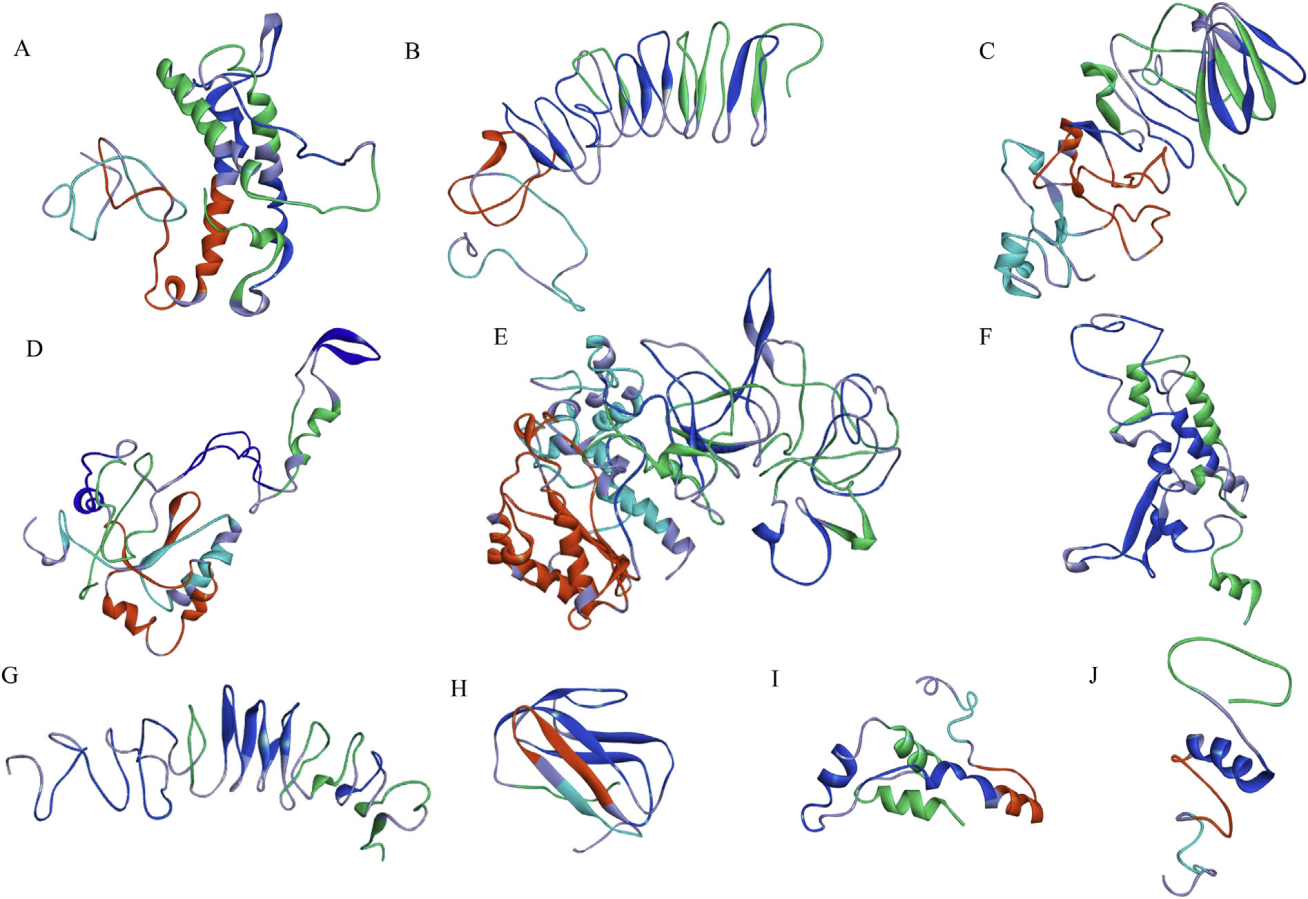
**Molecular dynamics simulation study of final multi-epitope constructs representing root mean square deviation**. A simulation was carried out for time duration of 20 ns. Representative graphs for construct 1, 2, 6 and 7 are provided.

We used online server PSIPRED to predict the secondary structure of the final vaccine constructs. The predicted α-helix, β-sheets and random coil of vaccine constructs have been provided in Table 3 and Figure 4 & S3. Tertiary structure of the final vaccine constructs was modeled using I- TASSER, RaptorX and Phyre (Figure 5). All the modeled structures were analyzed, and the common predicted structure was selected for further molecular simulation. I-TASSER modeled structures were found satisfactory for vaccine constructs (1, 2, 3, 4, 5, 7, 8, 9, & 10) with c-values of the best models -3.19, -2.68, 3.84, -2.84, -1.99, -1.69, -2.42, -2.59 and -2.51, respectively, while for construct 6, RaptorX modeled structure was chosen with a P-value of 2.78e-03, a lower p-value is indicative of best modelled structure [60].

### 2.8 Molecular dynamics simulation and tertiary structure validation

**Figure 6:**
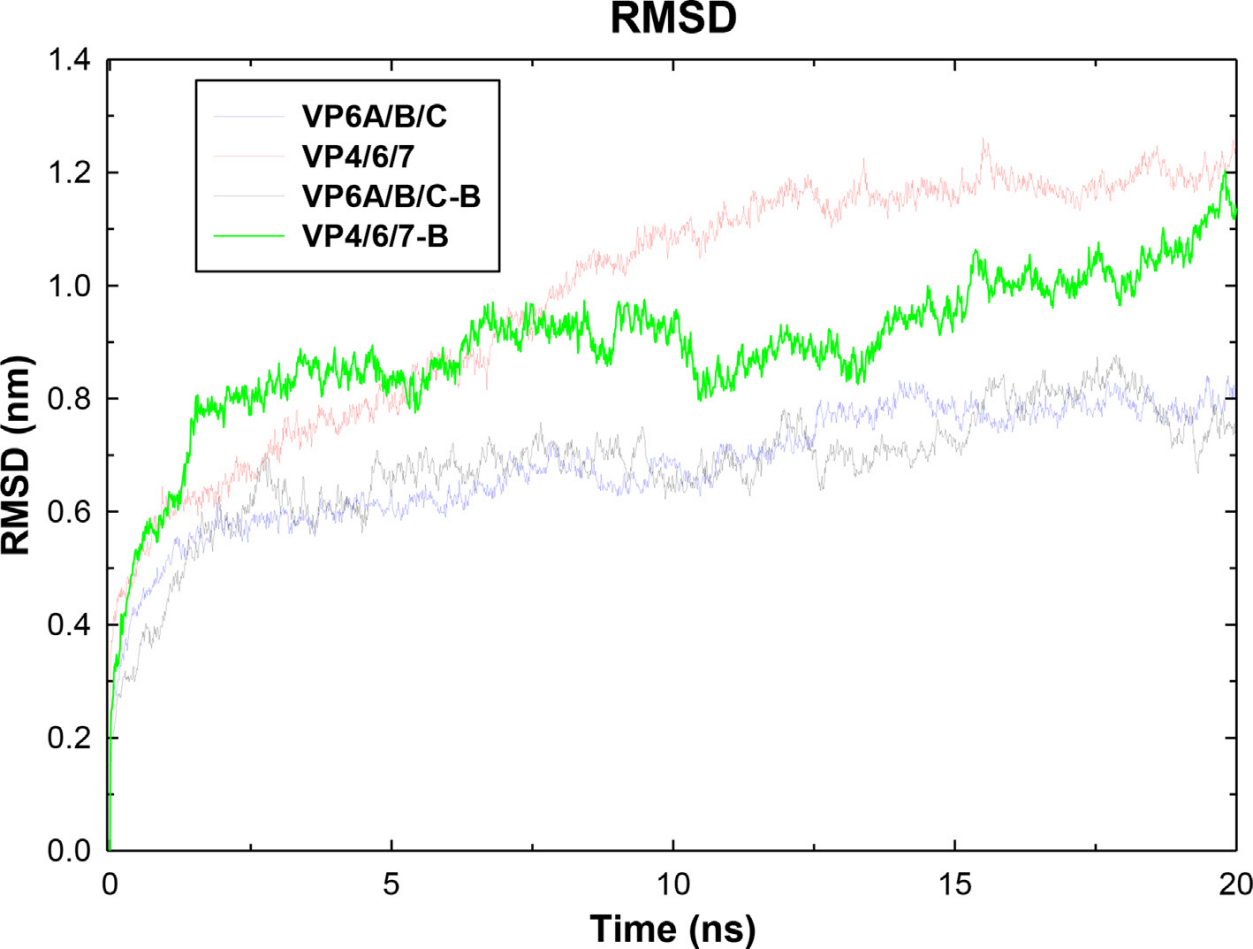
**Tertiary structure modeling and structure validation of multi-epitope constructs**. Cyan color represents CTL epitopes, orange represents HTL epitopes, blue represents linear B- cell epitopes and conformational B-cell epitope is highlighted with green. A. Construct 1; B. Construct 2; C. Construct 3; D. Construct 4; E. Construct 5; F. Construct 6; G. Construct 7; H. Construct 8; I. Construct 9; and J. Construct 10.

**Figure 7:**
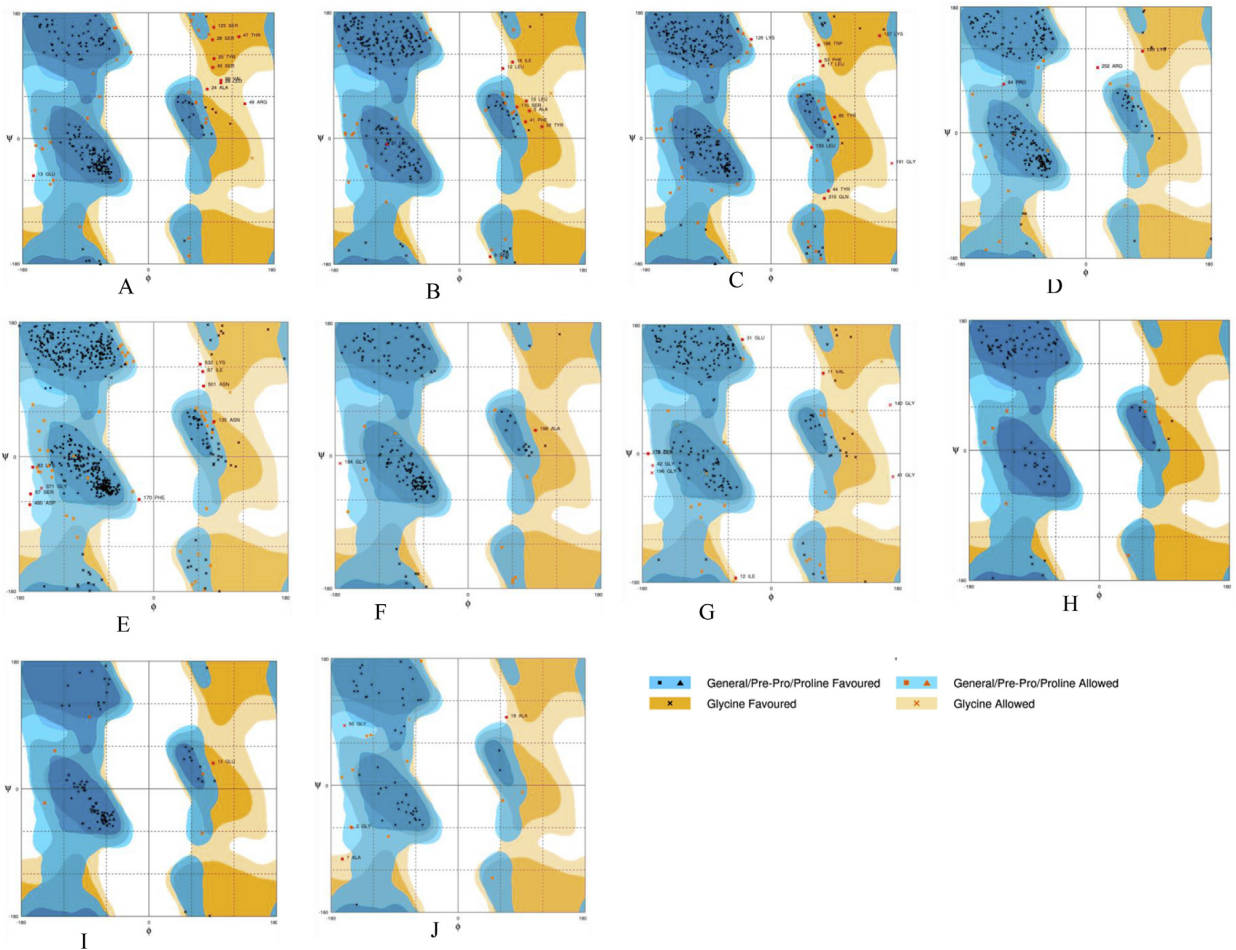
**Surface accessibility of linkers in the final multi-epitope constructs**. A. Construct 1; B. Construct 2; C. Construct 3; D. Construct 4; E. Construct 5; F. Construct 6; G. Construct 7; H. Construct 8; I. Construct 9; and J. Construct 10. Blue color represents AAY linker, cyan represents KK linker and GGGGS is represented by red color.

Further refinement and overall stability of multi-epitope subunit vaccine constructs were performed using molecular dynamics simulation in GROMACS, CHARMM27 force field and SPC/E water model as described previously. A plot of root square deviation (RMSD) against time reflects fluctuations generated within a time interval of 20 ns for all constructs and 40 ns for construct 5 having a predicted molecular weight of 62 kDa (Table 3). RMSD value of multi-subunit vaccine backbone was predicted to be 0.2–0.7 nm (Figure 6 and Figure S4) and the structure validation of final vaccine was carried out using RAMPAGE server (Figure 7). Table 4 provides the summary of distribution of amino acid residues in energetically favored area, allowed part and outlier region of vaccine constructs. The results of Ramachandran plots are suggestive of high structural quality due to the presence of a minimum steric atomic clashes between the residues in the refined vaccine constructs (Figure 7 and Table 4).

**Table 4:** Summary of amino acid residues of vaccine constructs in the energetically favored, allowed and residues in the outlier region as analyzed by Physico-chemical parameter of final multi-epitope constructs.

Multi-epitope antigen	No. of residues in the favored region	No. of residues in the allowed region	No. of residues in the outlier region
VP6A/B/C (Construct 1)	188 (82.8%)	29 (12.8%)	10 (4.4%)
VP4/6/7 (Construct 2)	242 (88.0%)	24 (8.7%)	9 (3.3%)
VP2/3/4/6/7 (Construct 3)	270 (85.7%)	35 (11.1%)	10 (3.2%)
NSP2/3/4/5 (Construct 4)	237 (92.2%)	17 (6.6%)	3 (1.2%)
VP2/3/4/6/7-NSP2/3/4/5 (Construct 5)	522 (91%)	42 (7.3%)	9 (1.6%)
VP6A/B/C–B (Construct 6)	184 (93.4%)	11 (5.6%)	2 (1.0%)
VP4/6/7-B (Construct 7)	193 (85.4%)	24 (10.6%)	9 (4.0%)
VP4/A (Construct 8)	101 (91.8%)	9 (8.2%)	0 (0.0%)
VP6/A (Construct 9)	93 (93.9%)	5 (5.1%)	1 (1.0%)
VP7/A (Construct 10)	50 (75.8%)	12 (18.2%)	4 (6.1%)

### 2.9 Surface accessibility and verification of conformational B-cell epitopes in the vaccine construct

**Figure 8:**
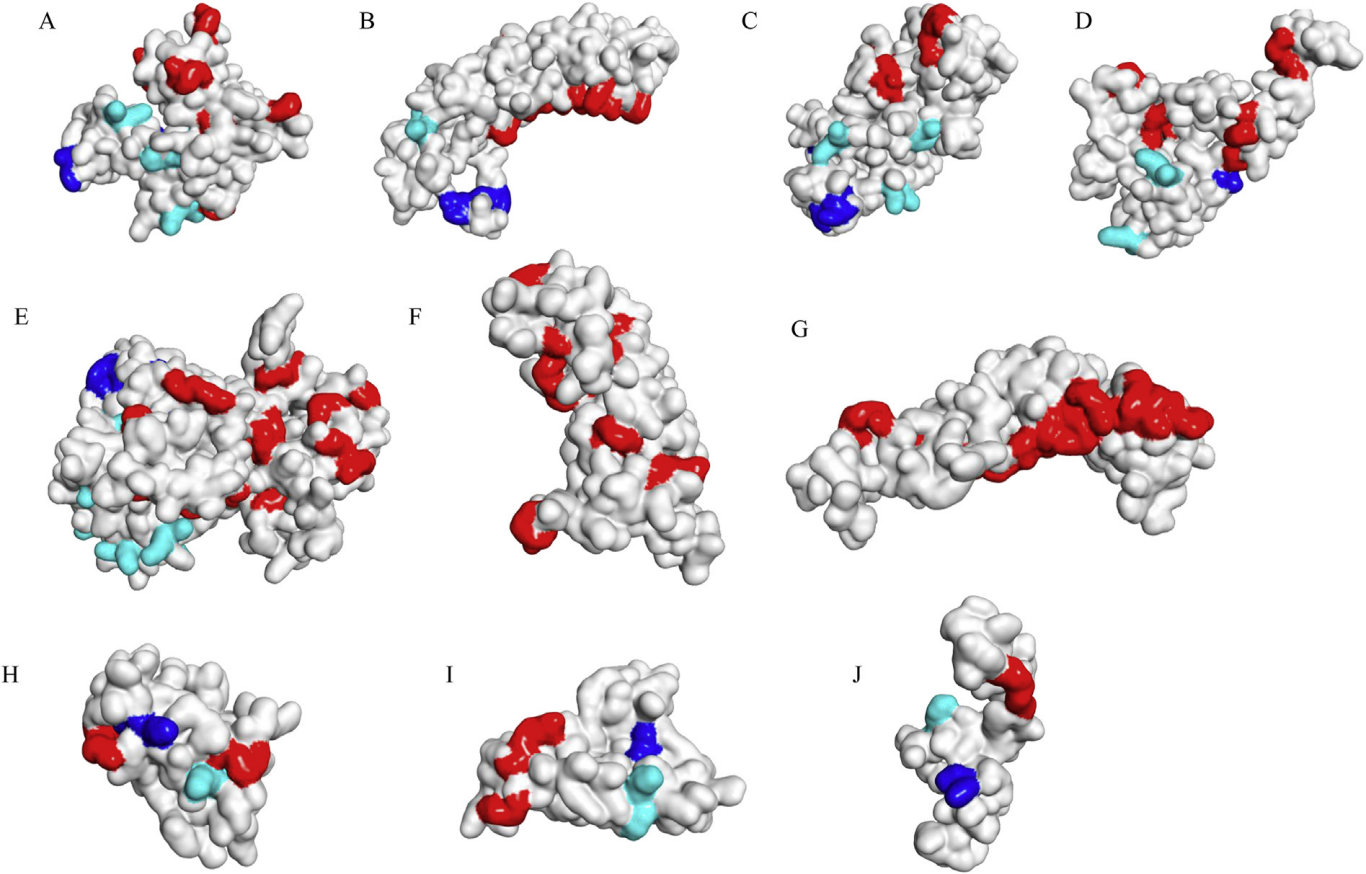
**Conformational B-cell epitopes prediction for the final multi-epitope constructs by Ellipro**. A. Construct 1, B. Construct 2, C. Construct 3, D. Construct 4, E. Construct 5, F. Construct 6, G. Construct 7, H. Construct 8, I. Construct 9 and J. Construct 10. The epitopes are represented as colored spheres in the final vaccine model where each color represents one epitope.

**Figure 9:**
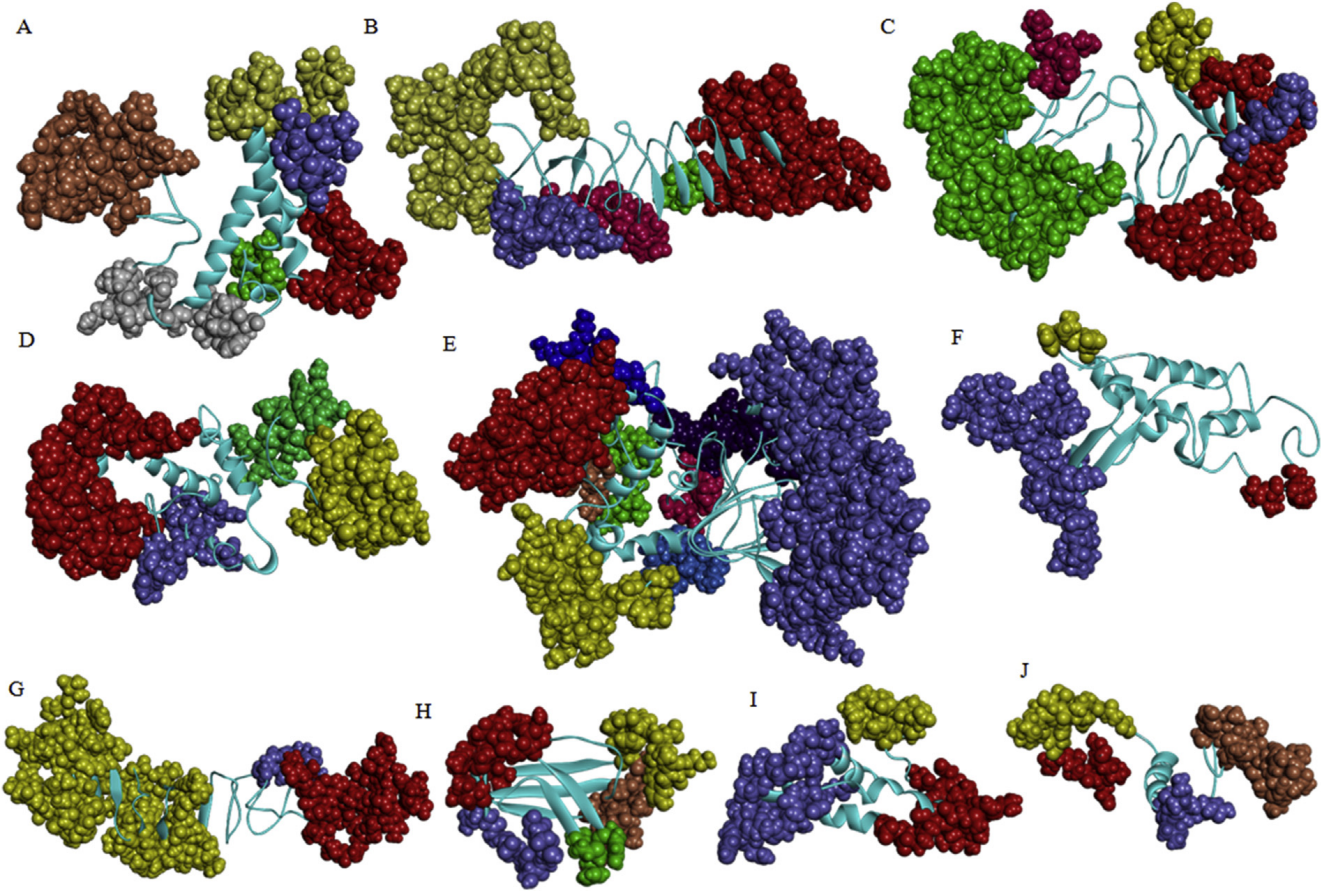
**Structure prediction and validation of final multi-epitope constructs**. Ramachandran plot analysis of the simulated structures. Summary of residues in favored, allowed and in outlier part is provided in Table 4.

Cathepsin and carboxypeptidase are involved in MHC class II antigen presentation pathway through proteolytic cleavage of dibasic (RR, KK, KR or RK) sites present in the endocytosed proteins [61]. MHC class II molecules expressed by antigen presenting cells are associated with presentation of processed peptides to CD4+ T cells. Proteases that are involved in MHC class II antigen presentation pathway exhibits preferential cleavage of substrates containing hydrophobic motifs (AAY). We found that the cleavable linker residues (AAY and KK) in the multi-epitope subunit vaccines were accessible suggesting that the probability of T- cell epitopes presentation by MHC molecules as predicted by discovery studio (Figure 8). Conformational B-cell epitopes that were included in the final vaccine construct was further verified with the help of four prediction servers -CBTOPE, Ellipro, Discotope and EPSVR. The results showed that the conformation epitopes were similarly predicted by CBTOPE, Ellipro, Discotope and EPSVR (Table S6). We have predicted an additional discontinuous B-cell epitopes with the help of Ellipro (Figure 9 and Table S7). ElliPro is a web-based server commonly used for prediction of an antibody epitopes in protein antigens [62,63].

### 2.10 Docking of vaccine constructs with receptor

**Figure 10:**
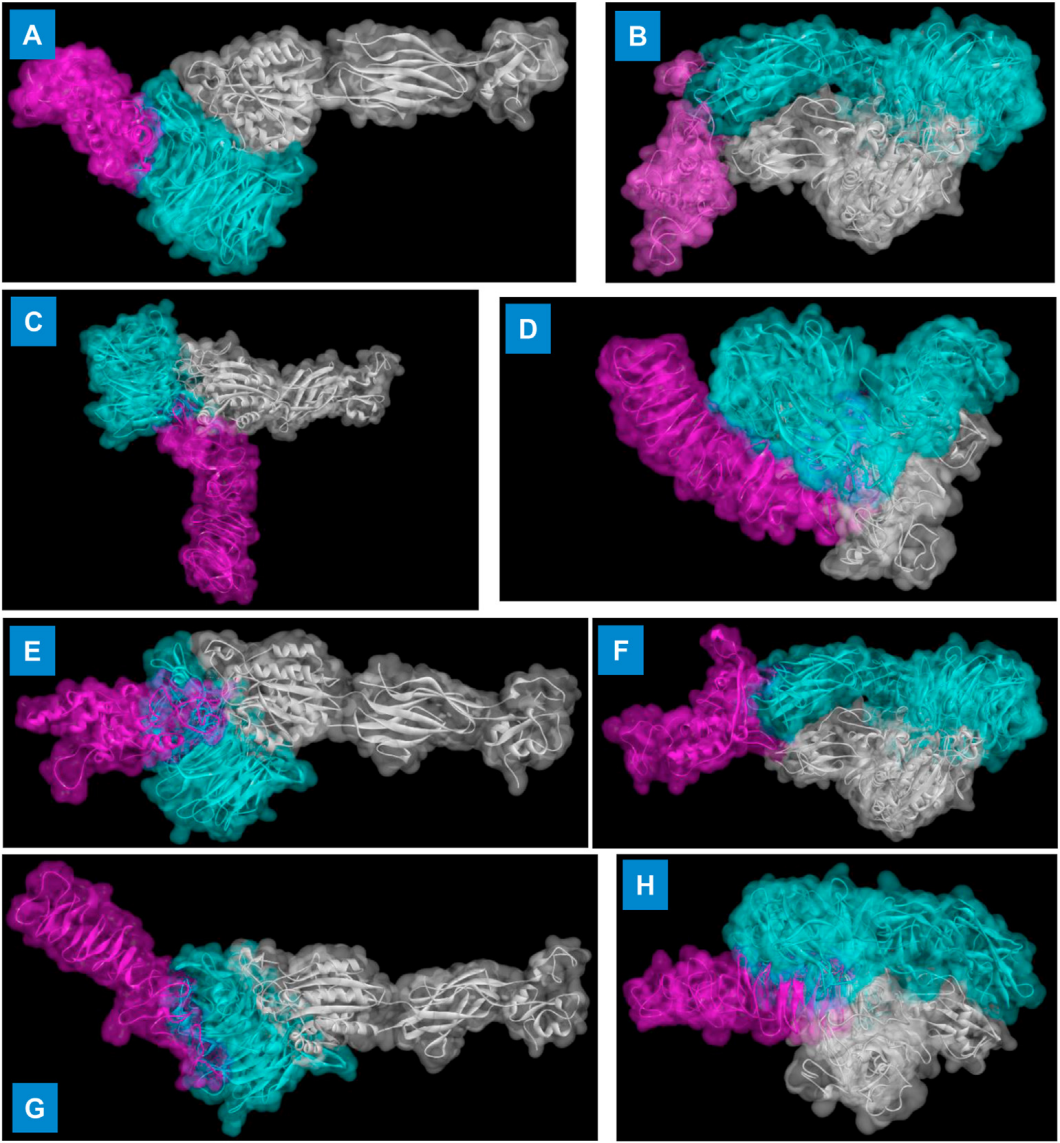
**Docked complex of multi-subunit vaccine constructs with integrin receptor**. A. Construct1 interaction with αIIbβ3 B. Construct 1 with α_V_β_3_ C. Construct 2 with αIIbβ3 D. Construct 2 with α_V_β_3_ E. Construct 6 with αIIbβ3 F. Construct 6 with α_V_β_3_ G. Construct 7 with αIIbβ3 H. Construct 7 with α_V_β_3_. Integrin receptor chain A and B has been shown in cyan and silver color, respectively, whereas magenta color represents the multi-epitope vaccine constructs in the docked complex.

Rotavirus entry is a multistep process involving the proteolytic cleavage of spike protein VP4 into two fragments VP5 and VP8, the interaction of these polypeptides and VP7 with integrins (αvβ3) and sialic acid including heat shock cognate protein [64,65]. Modeled and refined structures of vaccine constructs was used for molecular docking with integrin receptors using ClusPro v.2.0 docking program (www.cluspro.bu.edu) with default settings [47]. The interacting residues of four vaccine constructs with integrin receptor chain A and B is summarized in Table 5. All the four vaccine models have shown interactions with chain A of integrin subunit (αIIbβ3 and αVβ3) receptors (Figure 10) that are well known to mediate the entry of rotavirus involving VP4 and VP7 surface proteins [64,65].

**Table 5:** A list of interacting residues of docked multi-subunit vaccine constructs with integrin receptor complex.

Vaccine constructs	Integrin receptor	Binding energy (kcal mol-1)		Interacting residues of vaccine constructs with	
		Integrin receptor chain A	Integrin receptor chain B	Integrin receptor chain A	Integrin receptor chain B
Construct 1	αIIbβ3 (PDB ID: 2vdp)	-10.8	0	GLU75, ARG76, TYR145, VAL146, GLU147, THR148, ALA149,THR152, GLN220, PHE216, TRP218, GLU219, THR221, VAL227, ALA228,	-
Construct 1	αVβ3 (PDB ID: 4O02)	-8.5	-6.0	LEU9, GLU13, TYR20, THR21, ILE22, ASN23, THR38, ARG49, LEU50, SER51, ARG67	GLN53, MET55, ARG56, LYS58, ALA228
Construct 2	αIIbβ3 (PDB ID: 2vdp)	-9.4	-8.1	GLU4, ALA5, LYS8, SER9, ILE10, LEU12, LEU17, ALA18, TYR20, GLU25	LEU12, LEU13, TYR15, LEU17, ARG26, PHE27, SER28, TYR34, ASN59,
Construct 2	αVβ3 (PDB ID: 4O02)	-14.7	-10.4	ASP3, ALA31, TYR32, LYS35, LYS57, LYS58, ILE119, GLY120, GLY121, TRP125, GLY153, GLY171	LYS8, SER9, ILE11, ILE12, TYR20, GLU25, ARG26, LYS42
Construct 6	αIIbβ3 (PDB ID: 2vdp)	-11.1	-6.7	GLY23, GLY24, GLY25, SER26, TRP27, LEU29, ARG33, ARG35, ASN51, GLN53, GLY115, ASP141, GLU144, GLN145, HIS148, ARG149	LEU29, ASN31, ARG32
Construct 6	αVβ3 (PDB ID: 4O02)	-10.5	-7.1	ARG1, TYR81, TYR91, GLY148, LYS162, PHE186, PRO187, TRP188, LEU192, TYR195, ALA198, GLU200,	ASN80, TYR81, GLU83, ARG86
Construct 7	αIIbβ3 (PDB ID: 2vdp)	-20.1	0	GLY2, GLU4, ALA6, ALA7, ARG9, ARG15, ALA16, ALA18, ASN19, ASP21, GLY22, GLY25, TYR38, ILE39, GLY40, GLY41, SER44, TRP45, SER59, PRO60, ILE62, VAL63, THR64, GLU65, ALA66, GLN77, THR79,	-
Construct 7	αVβ3 (PDB ID: 4O02)	-14.4	-7.4	ARG132, ASP134, SER148, ASP149, GLN152, GLU167, ARG170, LYS185, ARG186, GLN206, LYS225, TRP226, TRP227, VAL229,	SER198, GLU210, LYS224, GLN228

### 2.11 Functional validations of predicted B and T cell epitopes based on published literature

Immunoinformatic approaches have commonly been used to identify potential B and T-cell epitopes that can help to induce humoral and cell-mediated immune responses. Neutralizing antibodies to VP4 and VP7 proteins are known to induce immunity against RV in natural infection in humans [5,66], anti-VP6 antibodies and CD4+ T cells have also been implicated in immune protection [67,68]. In this work we have identified a total of 4 linear B-cell epitopes, of which two linear epitopes (aa117-128 and aa144-155) and 2 conformational B-cell epitopes (aa151-159, aa157-167) forms a part of secreted soluble form of NSP4 (Table 1 and Table 2, Table S2a and S2b). It was previously shown that the secreted form of NSP4 (aa112–175) during rotavirus- infected cells was characterized as an enterotoxin of rotavirus protein. In a previous study an antibody to NSP4 aa112-175 was found to reduce the occurrence and severity of rotavirus-induced diarrhea in suckling mice pups [69]. It has been previously shown that a peptide (aa266-326) derived from VP7 can permeabilize artificial membranes leading to subsequent replication in virus-infected cells [70]. *In silico* analysis of rotavirus VP7 revealed the presence of potential linear (aa308-320), conformational B-cell (aa286-295) and CTL (aa316-324) epitopes in the membrane permeabilization domain (Table S2a and S2b). Antibody to such peptides might block membrane crossing by non-enveloped rotavirus during infection. Rotavirus VP7 protein has well defined antigenic epitopes namely 7-1 and 7-2. 7-1 epitope is subdivided into 7-1a and 7-1b [71]. Region 7-1 that spans the inter-subunit boundary is reported as an immunodominant epitope. Antibodies that target region 7-1 of VP7 probably neutralized entry of rotavirus through stabilization of VP7 trimer and inhibition of uncoating signal required for VP4 structural rearrangement [71]. Cytotoxic T lymphocytes specific to rotavirus is reported to play an important role in the clearance of rotavirus infection. Rotavirus VP7 protein was shown to induce a class I MHC-restricted CTL response and the CTL epitopes (aa5-13, aa8-16 and aa31-40) were mapped to H1 and H2 signal sequence of protein [72]. Using immunoinformatic tools we have identified and mapped CTL (aa15-23) and HTL (aa13-25) epitopes that were previously characterized as MHC class I epitopes of VP7 (Table 2). It has been shown that a synthetic peptide containing aa642 to 658 of VP5 can compete with the binding of the RRV to the heat shock cognate protein, HSC70 [73]. The VP5∗ subunit (aa308-310) of cleaved fragment of VP4 spike protein contains the α2β1 integrin (Asp-Gly-Glu) binding motif [74]. Synthetic peptides or antibodies to the regions spanning the predicted conformational (aa413-424) and linear B-cell epitope (aa657-667) of VP5 might provide a steric hindrance to rotavirus particle that uses α2β1 integrin as a receptor during entry (Table 2). VP6 protein is the most abundant and highly conserved group specific antigen of rotavirus. The sequence between amino acid residues 48 to 75 of VP6 has previously been characterized as immunodominant based on reactivity of monoclonal antibodies [75]. In the present study, we have identified aa68-81 as potential HTL epitope spanning the previously predicted antibody binding epitope of VP6 protein (Table S4b). In a previous study a synthetic peptide comprising of 14-amino acid spanning the region aa289-302 (RLSFQLVRPPNMTP) of VP6 protein was found to provide complete protection of mice against oral challenge of rotavirus [75]. Intriguingly, we predicted a 13-mer peptide (aa284-296) as potential HTL epitope of VP6 with a high conservancy (92%) among different rotavirus strains (Table 2).

VP4 and VP7 proteins are the primary targets of vaccine development and neutralizing antibodies against VP4 and VP7 proteins do not prevent rotavirus reinfection suggesting the possible role of other structural and nonstructural proteins. Previous literature have observed the presence of NSP2-specific IgA and IgG antibodies in more than 75% of naturally rotavirus infected children [76]. The region of NSP2 that interacts with NSP5 protein include the C-terminal α-helix, the loop between aa 291 and 302, the loops between aa 64 to 68 and aa 179 to 183 and the helix between residues 232 and 251 [77]. Using phage display, antibody-binding epitope aa244-252 has been mapped to the region on NSP2 protein known to interact with NSP5 during viroplasm formation in virus-infected cells [77]. NSP2 aa298–312 (linear epitope) and aa298-313 (conformational B- cell epitope) predicted as B-cell epitope with a conservancy of around 89% (Table 1 and Table S2a,b) might be useful for further experimental validations. The highly conserved C- terminal domain of rotavirus phosphoprotein NSP5 is required for viroplasm-like structure formation and is important for insolubility and hyperphosphorylation during rotavirus replication [78]. The findings of present *in silico* analysis revealed the presence of four overlapping HTL epitopes corresponding to NSP5 amino acid positions, aa175-193 using three independent prediction tools (Table S4b). NSP5 aa170-183 and aa173-184 that have been found to contain predicted linear and conformational B-cell epitope, respectively, this region of NSP5 is also predicted as HTL epitopes (Table S2a and S2b). Similarly, NSP5 aa2-10 (9-mer peptide) was predicted as CTL epitope, while the aa19-36 and aa66-85 of N-terminal region of NSP5 protein was predicted as conformational B-cell epitopes (Table S2b). Interestingly, N- (aa1–33) and C-terminal region (aa 131–198) of NSP5 was previously shown to involved in interaction with NSP2 during rotavirus infection [79]. Rotavirus NSP2 and NSP5 are required for viroplasm formation and targeting both proteins may provide therapeutic implications during rotavirus-infected cells.

### 2.12 Codon optimization, synthesis, expression, and affinity purification of chimeric constructs in *E. coli*

The vaccine construct was codon optimized as per *E. coli* (Strain ATCC 27325/DSM 5911/W3110/K12K12) strain using JCAT server and we found GC content of vaccine constructs 1 to 10 as 45.87%, 48.93%, 48.38%, 45.98%, 47.57%, 52.24%, 51.45, 47.67, 50.49 and 46.19%, respectively. GC content observed was in the range of 30–70% suggesting a minimal impact on transcriptional and translational efficiency. The value of codon adaptive index (CAI) for all vaccine constructs was 1 which is considered as good and satisfactory.

**Figure 11:**
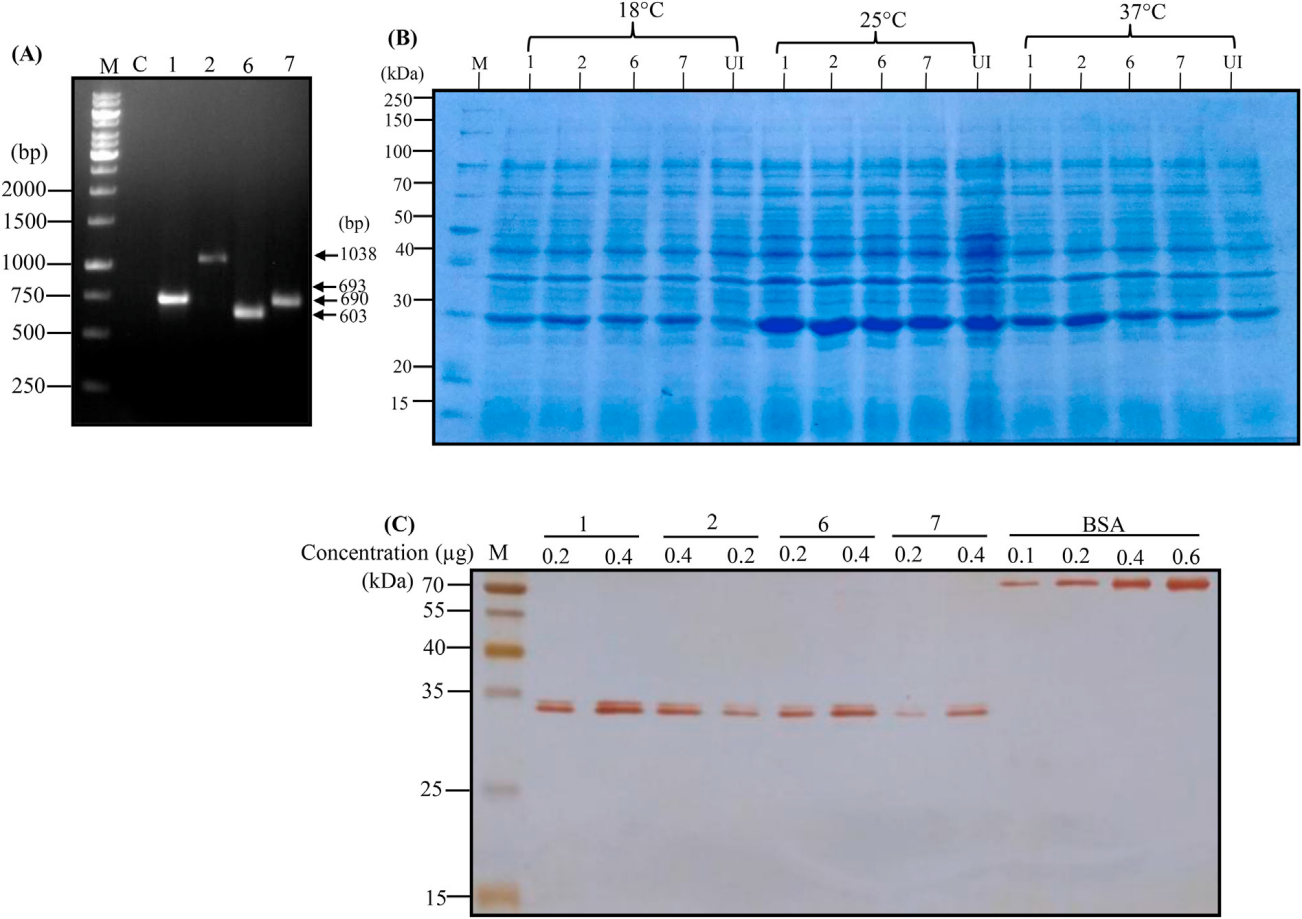
**Cloning, expression, and affinity purification of four chimeric constructs in *E. coli***. A) Confirmation of recombinant clones using PCR. Construct 1 (expected gene size 693 bp); Construct 2 (expected gene size 837 bp with 201 bp from vector sequence due to use of T7 forward primer), Construct 6 (expected gene size 603 bp) and Construct 7 (expected gene size 690 bp) were synthesized and cloned into champion pET directional TOPO expression vector (pET100/D-TOPO). C: Negative control without template DNA; M: GeneRuler 1 kb DNA ladder (SM0311, Thermo Scientific). B) SDS-PAGE analysis showing the expression of recombinant chimeric proteins induced with IPTG (200 μM) at 18 °C, 25 °C and 37 °C induction temperature. Construct 1 (expected size 28.9 kDa including tag); Construct 2 (expected size 34 kDa including tag); Construct 6 (expected size 28.9 kDa including tag); Construct 7 (expected size 34 kDa including tag); UI: uninduced *E. coli* whole cell lysates C) Silver stained-SDS-PAGE gel electrophoresis showing the purity of multi-epitope antigens. Different concentrations of BSA were loaded to determine the approximate concentration of purified proteins estimated using Bradford assay. M: Prestained protein ladder (Cat. 26616, ThermoScientific). See fig. S5 for full, uncropped image.

Of the 10 multi-subunit vaccines chimeric antigens designed, four constructs namely, construct 1, construct 2, construct 6 and construct 7 (Table 2) have been synthesized and cloned into champion pET directional TOPO expression system (pET100/D-TOPO) using manufacturer instructions (Thermofischer Scientific). The recombinant clones were verified by PCR using gene specific primers (Figure 11A). We found optimum expression and solubility of N-terminal 6xHis-tagged multi-subunit chimeric antigens induced with 200 μM concentration of IPTG at 25 °C temperature for 16 h (Figure 11B). Silver stained-SDS-PAGE gel electrophoresis confirmed the homogeneity of affinity purified chimeric proteins (Figure 11C and Figure S5) and different concentrations of BSA was loaded to determine the approximate concentration of purified proteins estimated using Bradford assay.

## 3 Conclusion

In this study, we have predicted and identified immuno-dominant antigenic fragments derived from 9 protein sequences of rotavirus structural (VP2, VP3, VP4, VP6 and VP7) and non-structural proteins (NSP2, NSP3, NSP4 and NSP5) that might have the abilities to induce immunity against rotavirus infection. As a part of our preliminary work we have cloned and expressed the multi-epitope vaccine constructs in *E. coli* and need to be experimentally validated for further use. Although the findings of present study is mainly based on computational prediction algorithms, but the immune epitopes presented herein will provide a platform for future experimental validations that may help to design peptide-based vaccine against rotavirus.

## 4 Materials and methods

### 4.1 Rotavirus protein sequence and selection of antigenic protein

In this study, the prototype simian group A rotavirus SA11 strain was used as reference strain to download sequences of structural (VP1, VP2, VP3, VP4, VP6 and VP7) and non-structural proteins (NSP1, NSP2, NSP3, NSP4 and NSP5); VP6 protein sequences of Adult diarrheal rotavirus (ADRV) and Cowden strain of porcine were included as group B and group C reference strains. The accession numbers of rotavirus proteins used for various bioinformatics analyses have been given in Table S1. All retrieved rotavirus protein sequence was analyzed for antigenicity using VaxiJen v2.0 server (http://ddg- pharmfac.net/vaxijen). VaxiJen is the first server used for prediction of whole protein antigenicity in an alignment-independent manner with high prediction accuracy of 70%–89% [80].

### 4.2 B-cell epitope prediction

Antigenic/immunogenic epitopes are specific part of an antigens that are recognized by the immune B-cell antibodies. We have predicted linear B-cell epitope using Bcepred server [81,82]. Bcepred predict B-cell epitopes based on combination of four parameters such as flexibility, hydrophilicity, polarity, and exposed surface with a prediction accuracy of about 58.7% (http://crdd.osdd.net/raghava/bcepred). We used full-length or partial crystallographic {VP4 {VP8 (PDB ID: 2P3I), VP5 (PDB ID: 2B4H)}, VP6 (PDB ID: 1QHD), VP7 (PDB ID: 3FMG), NSP2 (PDB ID: 1L9V), NSP3 (PDB ID: 1KNZ)} and modelled structures of rotavirus proteins to identify and predict a conformational B-cell epitopes [81]. Four different servers have been used to develop a reliable identification of conformational B-cell epitopes of rotavirus proteins [31,32,33,62]. CBTOPE server (http://crdd.osdd.net/raghava/cbtope) predicts B-cell epitope of an antigen using SVM-based model [83]. B-cell epitope prediction using DiscoTope 2.0 server (http://tools.iedb.org/discotope/) is based on estimation of surface accessibility with a default threshold setting value of -3.7. ElliPro (http://tools.iedb.org/ellipro/)predicts B-cell epitopes using the structure of an antigen. EPSVR (http://sysbio.unl.edu/EPSVR/) uses a Support Vector Regression (SVR) method to predict B-cell epitopes and shown to exhibit high performance with AUC value 0.597 as compared to the existing prediction servers [84]. Agadir score (http://agadir.crg.es/)was calculated for selection of potent epitopes based on its helical content [85].

### 4.3 Prediction of cytotoxic T lymphocytes and helper T-cell epitope

Cytotoxic T lymphocytes (CTL) epitopes of 9-mer peptide length were predicted using three different tools [81]. Proteasomal cleavage/TAP transport/MHC class I combined predictor (http://tools.iedb.org/processing) is a tool that predicts CTL epitopes based on combination of prediction scores obtained for each of proteasomal processing, TAP transport, and MHC binding^. The top 2% binders with an IC^50 ^less than or equal to^ 500nM was considered [64]. nHLAPred (http://crdd.osdd.net/raghava/nhlapred) predicts MHC I binding peptide using a neural network method [86]. Rankpep (http://imed.med.ucm.es/tools/rankpep.html)is used for prediction of peptides binding to MHC I and MHC II molecules using position specific scoring matrices and the top 2% MHC binders were selected [87]. Similarly, helper T-cell (HTL) epitopes were identified by NetMHCIIpan 3.1 (http://cbs.dtu.dk/services/NetMHCIIpan-3.1/). The threshold value for peptides with strong binding affinity was set as top 2%. ProPred (http://crdd.osdd.net/raghava/propred/) have been used to identify 9-mer promiscuous MHC II peptides based on quantitative matrices and the top 3% predicted peptides were selected as best binders [88]. Rankpep server predicted MHC class-II binding peptides top 5% best binders were selected in this study. A total of 27 HLA supertypes alleles with maximum population coverage (approximately >97%) were selected to identify and predict MHC I and II binding peptides [40,41,42,43,44].

### 4.4 Epitope immunogenicity, conservancy analysis and allergenicity assessment

Antigenicity of predicted epitopes in our vaccine constructs were assessed by VaxiJen v2.0. The identification of immunogenic epitopes is of great importance in understanding cellular immune responses and vaccine development. IEDB Class I immunogenicity tool (http://tools.iedb.org/immunogenicity/)was used to characterize the immunogenic potential of predicted 9-mer MHC I binding peptide [89]. We have predicted the immunogenicity (http://tools.iedb.org/CD4episcore) of MHC II binding epitopes using 7-allele method, immunogenicity method and combined method [45,46]. AllerTOP v2.0 (http://www.ddg-pharmfac.net/AllerTOP/) was used to predict the allergenic properties and the route of exposure of vaccine construct [85]. A web-based IEDB tool (http://tools.iedb.org/conservancy/)was used to predict the conservancy of epitopes that were included in the vaccine constructs [90].

### 4.5 Molecular docking and CTL/HTL mediated immunogenicity prediction

It is important to determine the strength of interaction of peptide-MHC molecules and the T-cell receptors. Molecular docking approach was used to identify and select the best CTL/HTL epitopes binding to MHC molecules. The 3D structure of peptide was generated by PEP-FOLD 2.0 (http://bioserv.rpbs.univ-paris-diderot.fr/services/PEP-FOLD/) and N- and C-terminals ends were capped [91]. We retrieved the PDB files of MHC molecules from Protein Data Bank for docking purpose. MHC allele lacking a crystal structure was modelled by I-TASSER (https://zhanglab.ccmb.med.umich.edu/I-TASSER/). MHC-peptide docking simulation was performed using ClusPro v.2.0 docking program (www.cluspro.bu.edu) with default settings [47]. Interaction energy was analyzed by prodigy (http://milou.science.uu.nl/services/PRODIGY/) [92].

### 4.6 Designing epitope-based vaccine constructs

Using the information of predicted epitopes, a multi-epitope-based vaccine was designed using high scoring peptide sequence consisting of T- and B-cell epitopes. Predicted immune epitopes were joined by cleavable and flexible linkers [30,31,62]. Integrin binding motif (RGD) fused with EAAAK linker was added as adjuvant and forms the component of final multi-epitope vaccine [39].

### 4.7 Structure prediction and validation

Physiochemical properties of vaccine constructs were analyzed using ProtParam [93]. Secondary structure of designed vaccine was predicted using the sequence of amino acid as input data by PSIPRED v3.3 (http://bioinf.cs.ucl.ac.uk/psipred/). Position-specific iterated BLAST (PSI-BLAST) was employed to select sequences exhibiting homology to vaccine constructs [94]. Modeling of vaccine constructs was done by I-TASSER [95], Phyre [96] and RaptorX. The best structures selected based on similarity of structure modelled by all three methods (I-TASSER, RaptorX, Phyre2) was used for validation and molecular dynamics simulation. We validated modelled structure by RAMPAGE server that provides a Ramachandran plots for glycine and proline amino acid residues. Ramachandran plot shows the distribution of torsion angles [psi (ψ) and phi (ϕ)] in a protein structure based on calculated van der Waal radius of the side chain [97].

### 4.8 Molecular dynamics simulation of epitope-based vaccine

Molecular dynamics (MD) is a computer simulation method that provides detailed information on the fluctuations and conformational changes of atoms and molecules. MD simulation of epitope-based vaccine was performed using GROMACS, v4.6.5 [98]. We used single point charge water molecules and CHARMM27 force field to determine the intermolecular interactions. Solvation was done in a cubic boxtype and appropriate number of chloride and sodium ions was used to neutralize peptide charges. Energy minimization was done using the steepest decent algorithm for 50000 steps with the maximum force of 1000 kJ/mol/nm. Equilibration of NVT (constant Number of particles, Volume and Temperature) and NPT (constant Number of particle, Pressure and Temperature) ensemble was done for 100 ps using Particle Mesh Ewald algorithm. After equilibrations of NVT at 300 K and NPT at 1 bar and production MD run was performed for 20 ns using LINCS (Linear Constant Solver) algorithm [98]. Root mean square deviation and root mean square fluctuation were performed to calculate standard deviation and fluctuation of the protein backbone with respect to time.

### 4.9 Codon optimization, synthesis, expression, and affinity purification of chimeric constructs in *E. coli*

Codon optimized epitope-based vaccine namely constructs 1, 2, 6 and 7 was cloned into the Champion pET directional TOPO expression cloning vector (pET100/D-TOPO, Thermofischer Scientific) to achieve high level expression in *E. coli*. Expression of N-terminal 6xHis-tagged multi-subunit chimeric antigens was induced at culture OD_600_ of 0.6 using different concentration of IPTG at 18 °C, 25 °C and 37 °C. Ni^2+^*-*affinity purification of soluble protein was carried out using Tris-NaCl buffer with 5 mM imidazole in the binding buffer and eluted with 250 mM imidazole, pH 7.4. The purified protein was dialyzed in a Tris buffer supplemented with NaCl. The purity of protein was analyzed on silver-stained SDS-PAGE and different concentrations of BSA was loaded to determine the approximate concentration of purified proteins estimated using Bradford assay.

## Declarations

### Author contribution statement

Yengkhom Damayanti Devi: Performed the experiments; Analyzed and interpreted the data; Wrote the paper.

Arpita Devi, Hemanga Gogoi, Bondita Dehingia: Performed the experiments.

Robin Doley: Contributed reagents, materials, analysis tools or data.

Alak Kumar Buragohain, Ch. Shyamsunder Singh, Partha Pratim Borah, C Durga Rao, Pratima Ray, George M. Varghese, Sachin Kumar: Analyzed and interpreted the data.

Nima D Namsa: Conceived and designed the experiments; Analyzed and interpreted the data; Contributed reagents, materials, analysis tools or data; Wrote the paper.

### Funding statement

Yengkhom Damayanti Devi was supported by UGC-CSIR Junior Research Fellowship for PhD. NDN duly acknowledged 10.13039/501100001407DBT, Govt. of India for partial financial support (Grant No. BT/338/NE/TBP/2012 dtd 14th August 2014).

### Data availability statement

Data included in article and supplementary material.

### Competing interest statement

The authors declare no conflict of interest.

### Additional information

No additional information is available for this paper.

## Appendix A Supplementary data

Sup_Figure Sup_Figure

Sup figure_revised Sup figure_revised
