# Supplementary material for: Exploring rotavirus proteome to identify potential B- and T-cell epitope using computational immunoinformatics
Source: Heliyon. 2020 Dec 29;6(12):e05760. doi: 10.1016/j.heliyon.2020.e05760 (PMC7779714; doi:10.1016/j.heliyon.2020.e05760)
Supplement: Sup_Figure [file mmc1.doc]

**Exploring rotavirus proteome to identify potential B- and T-cell epitope using computational immunoinformatics**

Damayanti Yengkhom Devi^1^, Arpita Devi^1^, Hemanga Gogoi^1^, Bondita Dehingia^1^, Robin Doley^1^, Alak Kumar Buragohain^2^, Ch. Shyamsunder Singh^3^, Partha Pratim Borah^4^, C Durga Rao^5^, Pratima Ray^6^, George M. Varghese^7^ Sachin Kumar^8^ and Nima D Namsa^1^

^1^Department of Molecular Biology and Biotechnology, Tezpur University, Napaam 784 028, Assam, India.

^2^Department of Biotechnology, Royal Global University, Guwahati, India

^3^Department of Paediatrics, Regional Institute of Medical Sciences, Imphal, India ^4^Department of Paediatrics and Neonatology, Pratiksha Hospital, Guwahati, India

^5^School of Liberal Arts and Basic Sciences, SRM University AP, Amaravati, India

^6^Department of Biotechnology, Jamia Hamdard, Delhi, India

^7^Department of Infectious Diseases, Christian Medical College, Vellore, India

^8^Department of Biosciences and Bioengineering, Indian Institute of Technology, Guwahati, India

Correspondence and requests for materials should be addressed to N.D. Namsa (email: [namsa@tezu.ernet.in](mailto:namsa@tezu.ernet.in))

#

# Additional supplementary information

**Table S1. Physico-chemical analysis and prediction of antigenicity of RV proteins.**

**Table S2a. Linear B-cell epitopes were predicted by Bcepred.** Four physico-chemical properties namely, hydrophilicity (Property 1), flexibility (Property 2), polarity (Property 3) and exposed surface (Property 4) were considered for prediction of B-cell epitopes. The selected epitopes used for designing multi-epitope vaccine constructs are highlighted in bold. **S2b**. **Predicted conformational B cell epitopes.** Conformational epitopes were predicted using four servers, one primary sequence dependent, CBTOPE (1) and three structure-dependent tools, Ellipro (2), Discotope (3) and EPSVR (4).

**Table S3. List of (a) HLA class I and (b) HLA class II supertype alleles used in this study. Table S4a. Predicted MHC I binding epitopes.** Three different tools were used and servers that predicted the corresponding epitope are numbered as IEDB Proteasomal cleavage/TAP transport/MHC class I combined predictor (1), nHLAPred (2) and RankPep (3). **S4b. Predicted MHC II binding epitopes.** Three different tools were used and servers that predicted the corresponding epitope are numbered as NetMHCpan 3.1 (1), ProPred (2) and RankPep (3).

# Table S5. Molecular docking of predicted CTL and HTL epitopes with (a) MHC I and (b) MHC II complexes.

**Table S6. Verification of conformational B-cell epitopes.** The epitopes in the final vaccine constructs were superimposed with their native structure by Phymol and Yale alignment server. The corresponding B-cell epitopes or part of the epitope predicted using four servers are numbered as CBTOPE (1), Ellipro (2), Discotope (3) and EPSVR (4).

# Table S7. Conformational B-cell epitopes prediction for the final multi-epitope vaccine construct by Ellipro

**Figure S1. Structure modeling of rotavirus proteins using I-TASSER.** A. VP2, B. VP3, C. VP4, D. VP7, E. NSP1, F. NSP3, G. NSP4 and H. NSP5.

**Figure S2. Localization of selected linear B-cell epitope in native rotavirus protein.** A. VP2; 189-AVENKNSRDAGK-200, B. VP3; 238-TIKLKQERWLGK-249, C. VP4; 208- IPRSEESKCTEYI-220, D. VP4; 241-RDVIHYRAQANED-253, E. VP4; 262-WKEMQYNRDI- 271, F. VP4; 657-PDIVTEASEKF-667, G. VP6; 9-KTLKDARDKIVEG-21, H. VP6; 139- WNLQNRRQRTG-149, I. VP6; 373-NYSPSREDNLQR-384, J. VP7; 308-QVMSKRSRSLNSA- 320, K. NSP2; 267-QNWYAFTSSMKQGNT-281, L. NSP3; 108-LSSKGIDQKMRVL-120, M. NSP4; 117-TTREIEQVELLK-128, N. NSP5; 170-KCKNCKYKKKYFAL-183, O. VP6 (Group A); 74-ISTDDYDDMRSGI-86, P. VP6 (Group B); 197-GMDSEHRFTVELKTR-211, Q. VP6 (Group C); 93-EAVCDDEIVREA-104, R. VP6 (Group C); 143-SRRENPVYEYKNPM-156.

**Figure S3. Graphical representation of secondary structure obtained for the final multi- epitope constructs.** A. Construct 3, 36.2% helix,11.0% sheet and 52.8% coil, B. Construct 4, 25.39% helix, 11.92% sheet and 62.69% coil, C. Construct 8, 11.5% helix, 32.7% sheet and

55.8% coil, D. Construct 9, 40.2 % helix, 2 % sheet and 57.8 % coil and E. Construct 10, 59.4 % helix, 11.6 % sheet and 29 % coil.

**Figure S4. Molecular dynamics simulation study of final multi-epitope constructs.** A. Simulation for construct 5 was performed for the time duration of 40 ns, while 20 ns for (B) constructs 3,4,8,9 and 10.

**Figure S5. Silver stained-SDS-PAGE gel showing the images of complete gel purity of multi-epitope antigens.** 1: Construct 1; 2: Construct 2; 6: Construct 6; 7: Construct 7. BSA: Bovine serum albumin protein was used as standard to estimate the approximate concentration of purified proteins. M: Prestained protein ladder (Cat. 26616, ThermoScientific).
