# Supplementary material for: Exploring rotavirus proteome to identify potential B- and T-cell epitope using computational immunoinformatics
Source: Heliyon. 2020 Dec 29;6(12):e05760. doi: 10.1016/j.heliyon.2020.e05760 (PMC7779714; doi:10.1016/j.heliyon.2020.e05760)
Supplement: Sup figure_revised [file mmc2.pptx]

## Slide 1
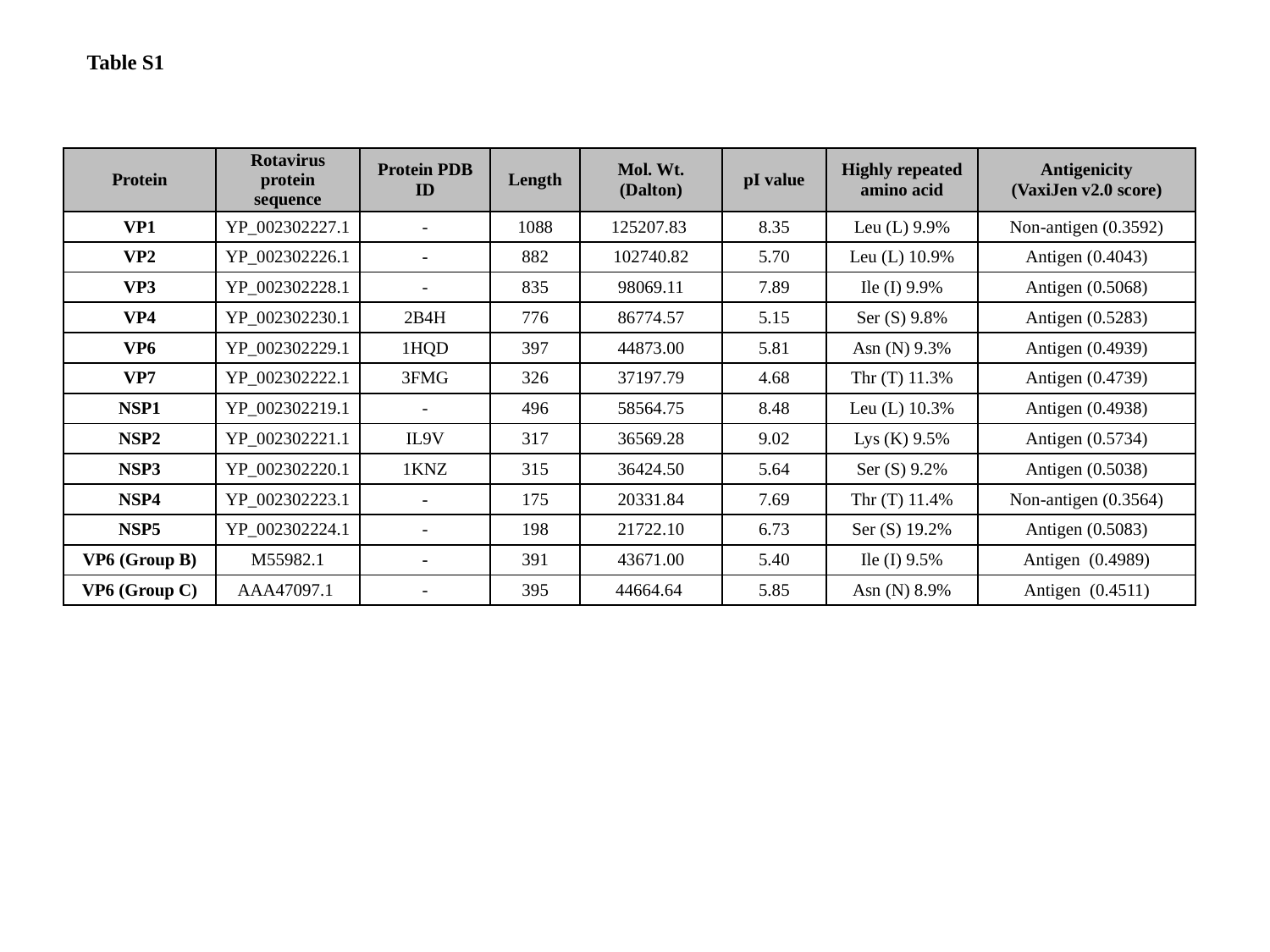

Table S1
| Protein | Rotavirus protein sequence | Protein PDB ID | Length | Mol. Wt. (Dalton) | pI value | Highly repeated amino acid | Antigenicity (VaxiJen v2.0 score) |
| --- | --- | --- | --- | --- | --- | --- | --- |
| VP1 | YP\_002302227.1 | - | 1088 | 125207.83 | 8.35 | Leu (L) 9.9% | Non-antigen (0.3592) |
| VP2 | YP\_002302226.1 | - | 882 | 102740.82 | 5.70 | Leu (L) 10.9% | Antigen (0.4043) |
| VP3 | YP\_002302228.1 | - | 835 | 98069.11 | 7.89 | Ile (I) 9.9% | Antigen (0.5068) |
| VP4 | YP\_002302230.1 | 2B4H | 776 | 86774.57 | 5.15 | Ser (S) 9.8% | Antigen (0.5283) |
| VP6 | YP\_002302229.1 | 1HQD | 397 | 44873.00 | 5.81 | Asn (N) 9.3% | Antigen (0.4939) |
| VP7 | YP\_002302222.1 | 3FMG | 326 | 37197.79 | 4.68 | Thr (T) 11.3% | Antigen (0.4739) |
| NSP1 | YP\_002302219.1 | - | 496 | 58564.75 | 8.48 | Leu (L) 10.3% | Antigen (0.4938) |
| NSP2 | YP\_002302221.1 | IL9V | 317 | 36569.28 | 9.02 | Lys (K) 9.5% | Antigen (0.5734) |
| NSP3 | YP\_002302220.1 | 1KNZ | 315 | 36424.50 | 5.64 | Ser (S) 9.2% | Antigen (0.5038) |
| NSP4 | YP\_002302223.1 | - | 175 | 20331.84 | 7.69 | Thr (T) 11.4% | Non-antigen (0.3564) |
| NSP5 | YP\_002302224.1 | - | 198 | 21722.10 | 6.73 | Ser (S) 19.2% | Antigen (0.5083) |
| VP6 (Group B) | M55982.1 | - | 391 | 43671.00 | 5.40 | Ile (I) 9.5% | Antigen (0.4989) |
| VP6 (Group C) | AAA47097.1 | - | 395 | 44664.64 | 5.85 | Asn (N) 8.9% | Antigen (0.4511) |

## Slide 2
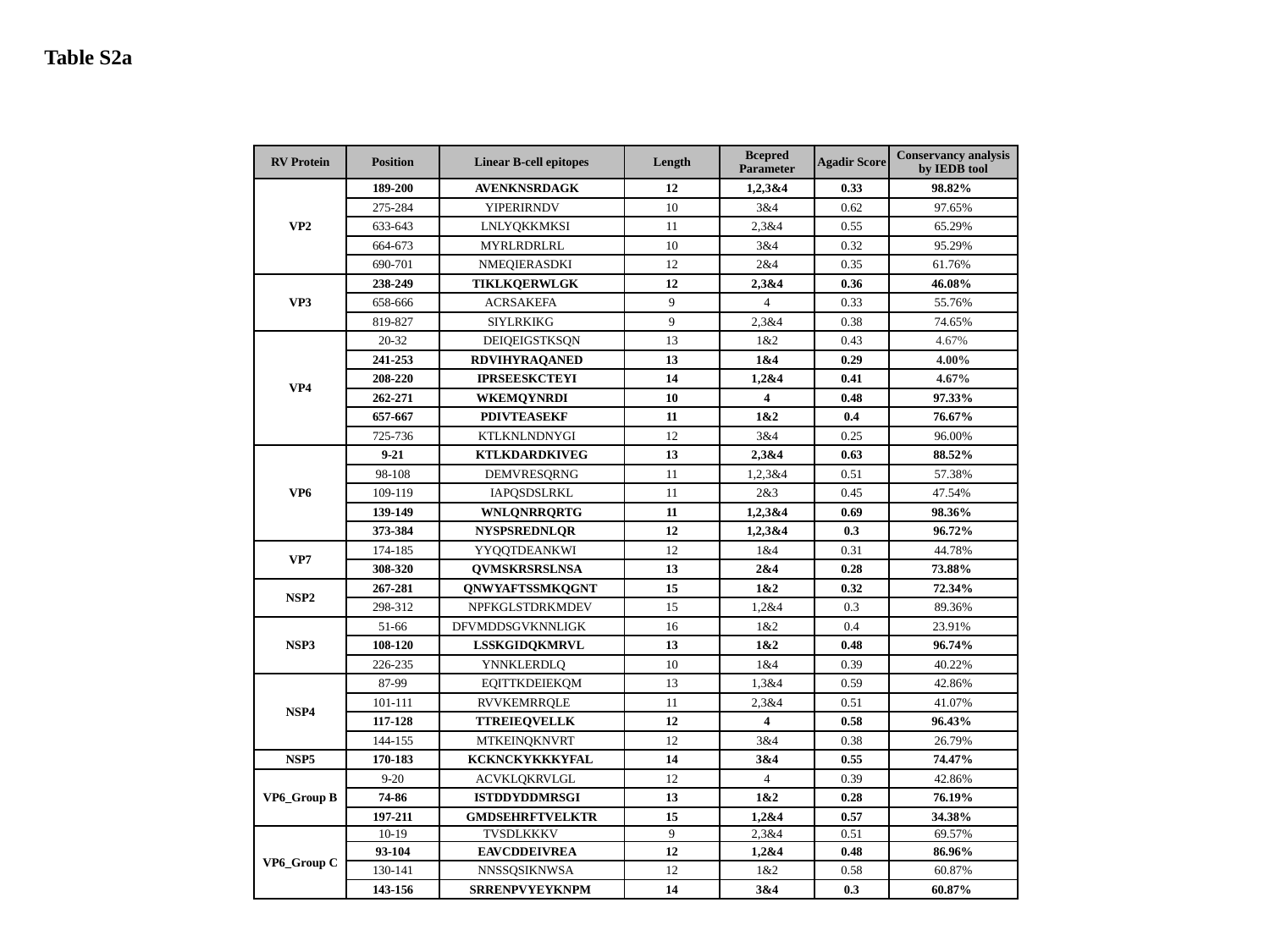

Table S2a
| RV Protein | Position | Linear B-cell epitopes | Length | Bcepred Parameter | Agadir Score | Conservancy analysis by IEDB tool |
| --- | --- | --- | --- | --- | --- | --- |
| VP2 | 189-200 | AVENKNSRDAGK | 12 | 1,2,3&4 | 0.33 | 98.82% |
| | 275-284 | YIPERIRNDV | 10 | 3&4 | 0.62 | 97.65% |
| | 633-643 | LNLYQKKMKSI | 11 | 2,3&4 | 0.55 | 65.29% |
| | 664-673 | MYRLRDRLRL | 10 | 3&4 | 0.32 | 95.29% |
| | 690-701 | NMEQIERASDKI | 12 | 2&4 | 0.35 | 61.76% |
| VP3 | 238-249 | TIKLKQERWLGK | 12 | 2,3&4 | 0.36 | 46.08% |
| | 658-666 | ACRSAKEFA | 9 | 4 | 0.33 | 55.76% |
| | 819-827 | SIYLRKIKG | 9 | 2,3&4 | 0.38 | 74.65% |
| VP4 | 20-32 | DEIQEIGSTKSQN | 13 | 1&2 | 0.43 | 4.67% |
| | 241-253 | RDVIHYRAQANED | 13 | 1&4 | 0.29 | 4.00% |
| | 208-220 | IPRSEESKCTEYI | 14 | 1,2&4 | 0.41 | 4.67% |
| | 262-271 | WKEMQYNRDI | 10 | 4 | 0.48 | 97.33% |
| | 657-667 | PDIVTEASEKF | 11 | 1&2 | 0.4 | 76.67% |
| | 725-736 | KTLKNLNDNYGI | 12 | 3&4 | 0.25 | 96.00% |
| VP6 | 9-21 | KTLKDARDKIVEG | 13 | 2,3&4 | 0.63 | 88.52% |
| | 98-108 | DEMVRESQRNG | 11 | 1,2,3&4 | 0.51 | 57.38% |
| | 109-119 | IAPQSDSLRKL | 11 | 2&3 | 0.45 | 47.54% |
| | 139-149 | WNLQNRRQRTG | 11 | 1,2,3&4 | 0.69 | 98.36% |
| | 373-384 | NYSPSREDNLQR | 12 | 1,2,3&4 | 0.3 | 96.72% |
| VP7 | 174-185 | YYQQTDEANKWI | 12 | 1&4 | 0.31 | 44.78% |
| | 308-320 | QVMSKRSRSLNSA | 13 | 2&4 | 0.28 | 73.88% |
| NSP2 | 267-281 | QNWYAFTSSMKQGNT | 15 | 1&2 | 0.32 | 72.34% |
| | 298-312 | NPFKGLSTDRKMDEV | 15 | 1,2&4 | 0.3 | 89.36% |
| NSP3 | 51-66 | DFVMDDSGVKNNLIGK | 16 | 1&2 | 0.4 | 23.91% |
| | 108-120 | LSSKGIDQKMRVL | 13 | 1&2 | 0.48 | 96.74% |
| | 226-235 | YNNKLERDLQ | 10 | 1&4 | 0.39 | 40.22% |
| NSP4 | 87-99 | EQITTKDEIEKQM | 13 | 1,3&4 | 0.59 | 42.86% |
| | 101-111 | RVVKEMRRQLE | 11 | 2,3&4 | 0.51 | 41.07% |
| | 117-128 | TTREIEQVELLK | 12 | 4 | 0.58 | 96.43% |
| | 144-155 | MTKEINQKNVRT | 12 | 3&4 | 0.38 | 26.79% |
| NSP5 | 170-183 | KCKNCKYKKKYFAL | 14 | 3&4 | 0.55 | 74.47% |
| VP6\_Group B | 9-20 | ACVKLQKRVLGL | 12 | 4 | 0.39 | 42.86% |
| | 74-86 | ISTDDYDDMRSGI | 13 | 1&2 | 0.28 | 76.19% |
| | 197-211 | GMDSEHRFTVELKTR | 15 | 1,2&4 | 0.57 | 34.38% |
| VP6\_Group C | 10-19 | TVSDLKKKV | 9 | 2,3&4 | 0.51 | 69.57% |
| | 93-104 | EAVCDDEIVREA | 12 | 1,2&4 | 0.48 | 86.96% |
| | 130-141 | NNSSQSIKNWSA | 12 | 1&2 | 0.58 | 60.87% |
| | 143-156 | SRRENPVYEYKNPM | 14 | 3&4 | 0.3 | 60.87% |

## Slide 3
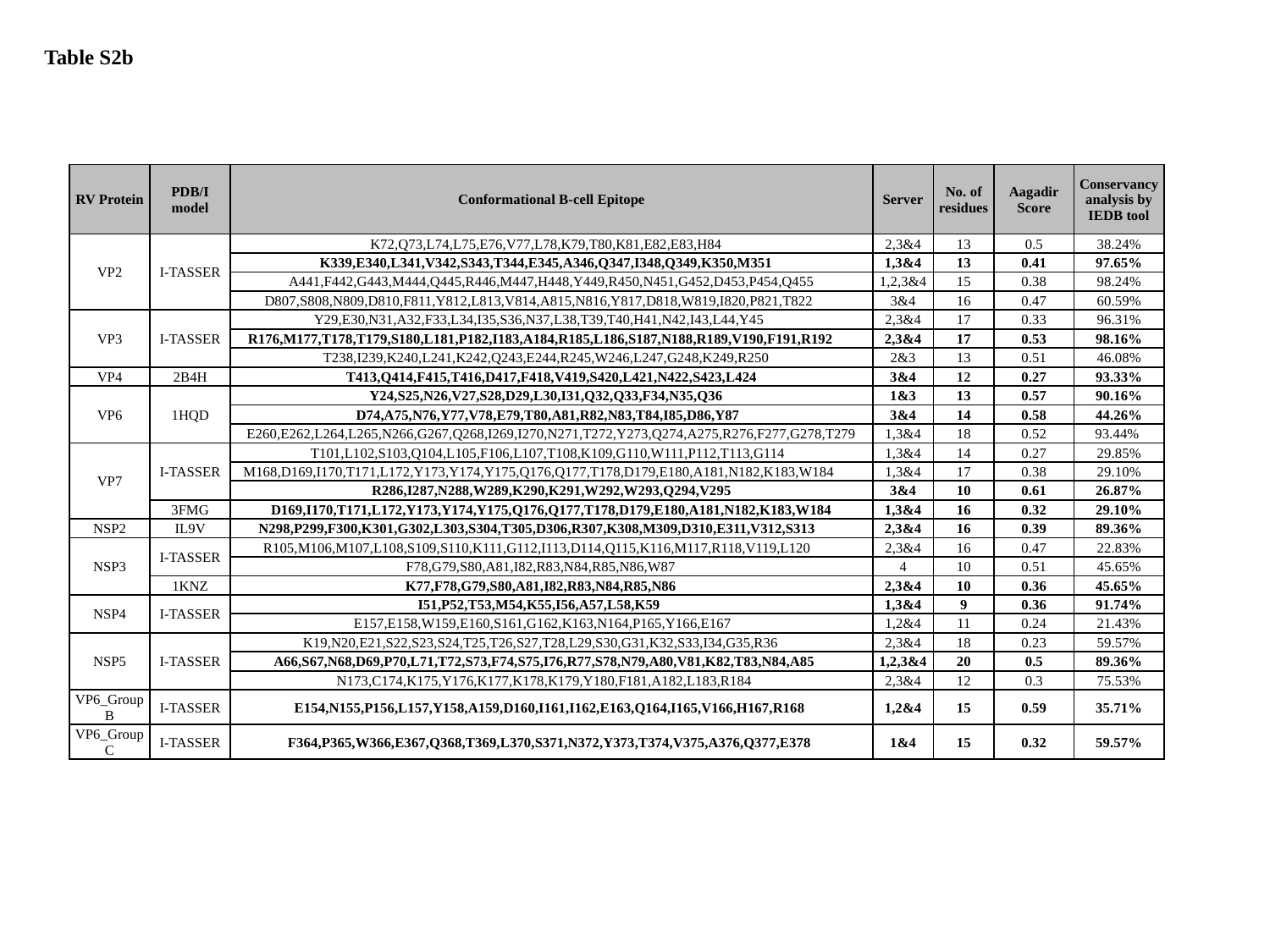

Table S2b
| RV Protein | PDB/I model | Conformational B-cell Epitope | Server | No. of residues | Aagadir Score | Conservancy analysis by IEDB tool |
| --- | --- | --- | --- | --- | --- | --- |
| VP2 | I-TASSER | K72,Q73,L74,L75,E76,V77,L78,K79,T80,K81,E82,E83,H84 | 2,3&4 | 13 | 0.5 | 38.24% |
| | | K339,E340,L341,V342,S343,T344,E345,A346,Q347,I348,Q349,K350,M351 | 1,3&4 | 13 | 0.41 | 97.65% |
| | | A441,F442,G443,M444,Q445,R446,M447,H448,Y449,R450,N451,G452,D453,P454,Q455 | 1,2,3&4 | 15 | 0.38 | 98.24% |
| | | D807,S808,N809,D810,F811,Y812,L813,V814,A815,N816,Y817,D818,W819,I820,P821,T822 | 3&4 | 16 | 0.47 | 60.59% |
| VP3 | I-TASSER | Y29,E30,N31,A32,F33,L34,I35,S36,N37,L38,T39,T40,H41,N42,I43,L44,Y45 | 2,3&4 | 17 | 0.33 | 96.31% |
| | | R176,M177,T178,T179,S180,L181,P182,I183,A184,R185,L186,S187,N188,R189,V190,F191,R192 | 2,3&4 | 17 | 0.53 | 98.16% |
| | | T238,I239,K240,L241,K242,Q243,E244,R245,W246,L247,G248,K249,R250 | 2&3 | 13 | 0.51 | 46.08% |
| VP4 | 2B4H | T413,Q414,F415,T416,D417,F418,V419,S420,L421,N422,S423,L424 | 3&4 | 12 | 0.27 | 93.33% |
| VP6 | 1HQD | Y24,S25,N26,V27,S28,D29,L30,I31,Q32,Q33,F34,N35,Q36 | 1&3 | 13 | 0.57 | 90.16% |
| | | D74,A75,N76,Y77,V78,E79,T80,A81,R82,N83,T84,I85,D86,Y87 | 3&4 | 14 | 0.58 | 44.26% |
| | | E260,E262,L264,L265,N266,G267,Q268,I269,I270,N271,T272,Y273,Q274,A275,R276,F277,G278,T279 | 1,3&4 | 18 | 0.52 | 93.44% |
| VP7 | I-TASSER | T101,L102,S103,Q104,L105,F106,L107,T108,K109,G110,W111,P112,T113,G114 | 1,3&4 | 14 | 0.27 | 29.85% |
| | | M168,D169,I170,T171,L172,Y173,Y174,Y175,Q176,Q177,T178,D179,E180,A181,N182,K183,W184 | 1,3&4 | 17 | 0.38 | 29.10% |
| | | R286,I287,N288,W289,K290,K291,W292,W293,Q294,V295 | 3&4 | 10 | 0.61 | 26.87% |
| | 3FMG | D169,I170,T171,L172,Y173,Y174,Y175,Q176,Q177,T178,D179,E180,A181,N182,K183,W184 | 1,3&4 | 16 | 0.32 | 29.10% |
| NSP2 | IL9V | N298,P299,F300,K301,G302,L303,S304,T305,D306,R307,K308,M309,D310,E311,V312,S313 | 2,3&4 | 16 | 0.39 | 89.36% |
| NSP3 | I-TASSER | R105,M106,M107,L108,S109,S110,K111,G112,I113,D114,Q115,K116,M117,R118,V119,L120 | 2,3&4 | 16 | 0.47 | 22.83% |
| | | F78,G79,S80,A81,I82,R83,N84,R85,N86,W87 | 4 | 10 | 0.51 | 45.65% |
| | 1KNZ | K77,F78,G79,S80,A81,I82,R83,N84,R85,N86 | 2,3&4 | 10 | 0.36 | 45.65% |
| NSP4 | I-TASSER | I51,P52,T53,M54,K55,I56,A57,L58,K59 | 1,3&4 | 9 | 0.36 | 91.74% |
| | | E157,E158,W159,E160,S161,G162,K163,N164,P165,Y166,E167 | 1,2&4 | 11 | 0.24 | 21.43% |
| NSP5 | I-TASSER | K19,N20,E21,S22,S23,S24,T25,T26,S27,T28,L29,S30,G31,K32,S33,I34,G35,R36 | 2,3&4 | 18 | 0.23 | 59.57% |
| | | A66,S67,N68,D69,P70,L71,T72,S73,F74,S75,I76,R77,S78,N79,A80,V81,K82,T83,N84,A85 | 1,2,3&4 | 20 | 0.5 | 89.36% |
| | | N173,C174,K175,Y176,K177,K178,K179,Y180,F181,A182,L183,R184 | 2,3&4 | 12 | 0.3 | 75.53% |
| VP6\_Group B | I-TASSER | E154,N155,P156,L157,Y158,A159,D160,I161,I162,E163,Q164,I165,V166,H167,R168 | 1,2&4 | 15 | 0.59 | 35.71% |
| VP6\_Group C | I-TASSER | F364,P365,W366,E367,Q368,T369,L370,S371,N372,Y373,T374,V375,A376,Q377,E378 | 1&4 | 15 | 0.32 | 59.57% |

## Slide 4
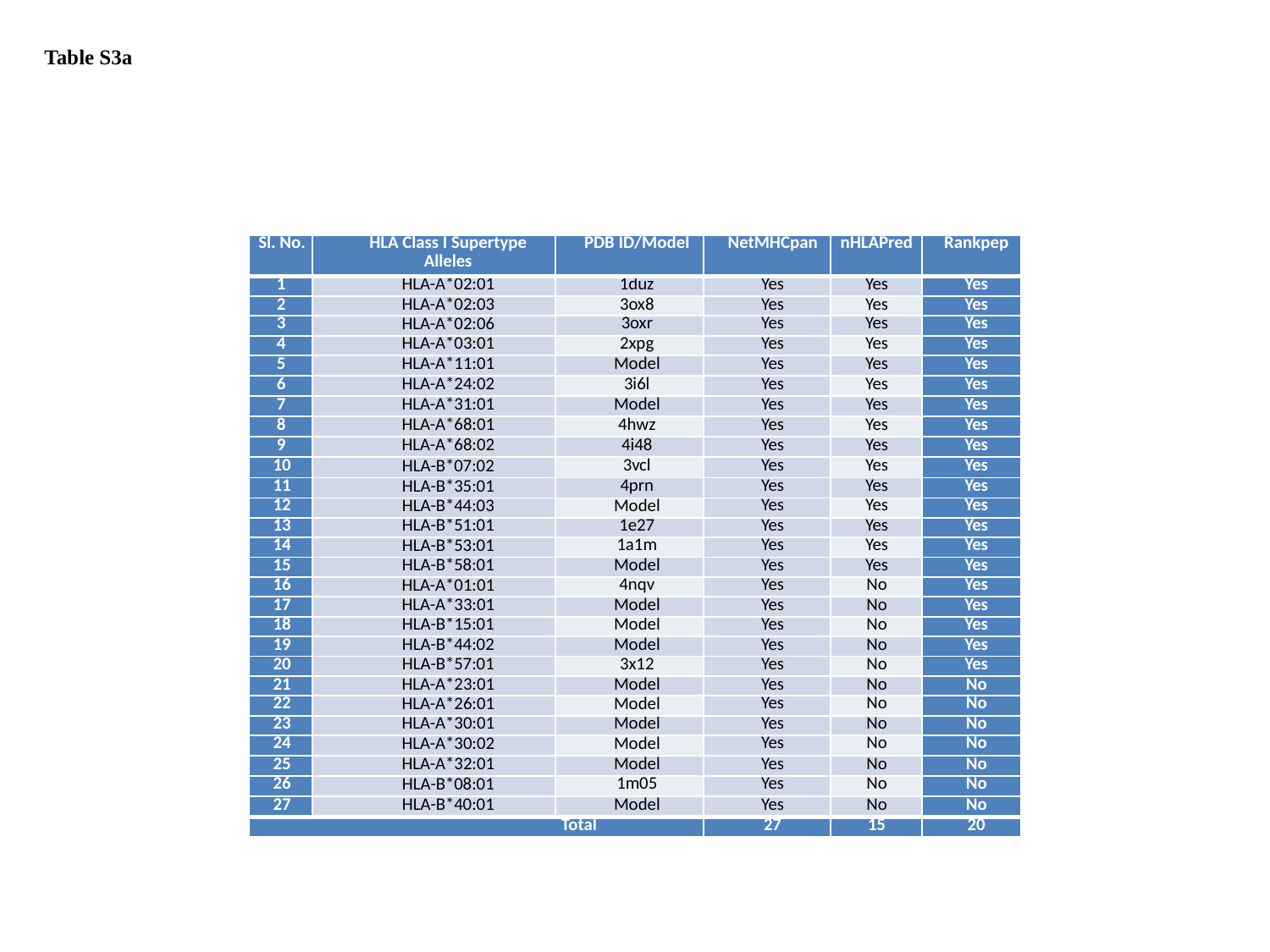

Table S3a
| Sl. No. | HLA Class I Supertype Alleles | PDB ID/Model | NetMHCpan | nHLAPred | Rankpep |
| --- | --- | --- | --- | --- | --- |
| 1 | HLA-A\*02:01 | 1duz | Yes | Yes | Yes |
| 2 | HLA-A\*02:03 | 3ox8 | Yes | Yes | Yes |
| 3 | HLA-A\*02:06 | 3oxr | Yes | Yes | Yes |
| 4 | HLA-A\*03:01 | 2xpg | Yes | Yes | Yes |
| 5 | HLA-A\*11:01 | Model | Yes | Yes | Yes |
| 6 | HLA-A\*24:02 | 3i6l | Yes | Yes | Yes |
| 7 | HLA-A\*31:01 | Model | Yes | Yes | Yes |
| 8 | HLA-A\*68:01 | 4hwz | Yes | Yes | Yes |
| 9 | HLA-A\*68:02 | 4i48 | Yes | Yes | Yes |
| 10 | HLA-B\*07:02 | 3vcl | Yes | Yes | Yes |
| 11 | HLA-B\*35:01 | 4prn | Yes | Yes | Yes |
| 12 | HLA-B\*44:03 | Model | Yes | Yes | Yes |
| 13 | HLA-B\*51:01 | 1e27 | Yes | Yes | Yes |
| 14 | HLA-B\*53:01 | 1a1m | Yes | Yes | Yes |
| 15 | HLA-B\*58:01 | Model | Yes | Yes | Yes |
| 16 | HLA-A\*01:01 | 4nqv | Yes | No | Yes |
| 17 | HLA-A\*33:01 | Model | Yes | No | Yes |
| 18 | HLA-B\*15:01 | Model | Yes | No | Yes |
| 19 | HLA-B\*44:02 | Model | Yes | No | Yes |
| 20 | HLA-B\*57:01 | 3x12 | Yes | No | Yes |
| 21 | HLA-A\*23:01 | Model | Yes | No | No |
| 22 | HLA-A\*26:01 | Model | Yes | No | No |
| 23 | HLA-A\*30:01 | Model | Yes | No | No |
| 24 | HLA-A\*30:02 | Model | Yes | No | No |
| 25 | HLA-A\*32:01 | Model | Yes | No | No |
| 26 | HLA-B\*08:01 | 1m05 | Yes | No | No |
| 27 | HLA-B\*40:01 | Model | Yes | No | No |
| Total | | | 27 | 15 | 20 |

## Slide 5
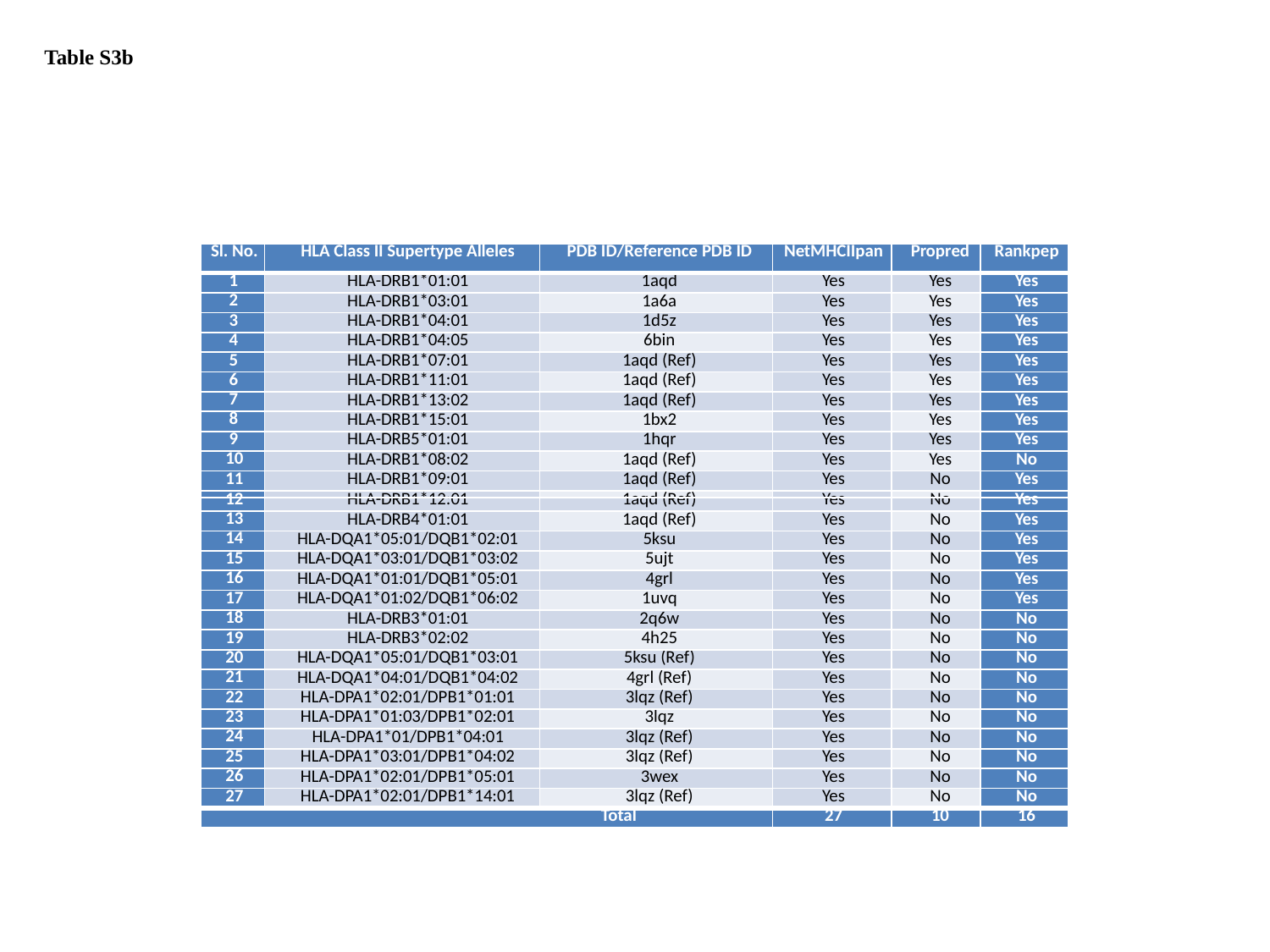

Table S3b
| Sl. No. | HLA Class II Supertype Alleles | PDB ID/Reference PDB ID | NetMHCIIpan | Propred | Rankpep |
| --- | --- | --- | --- | --- | --- |
| 1 | HLA-DRB1\*01:01 | 1aqd | Yes | Yes | Yes |
| 2 | HLA-DRB1\*03:01 | 1a6a | Yes | Yes | Yes |
| 3 | HLA-DRB1\*04:01 | 1d5z | Yes | Yes | Yes |
| 4 | HLA-DRB1\*04:05 | 6bin | Yes | Yes | Yes |
| 5 | HLA-DRB1\*07:01 | 1aqd (Ref) | Yes | Yes | Yes |
| 6 | HLA-DRB1\*11:01 | 1aqd (Ref) | Yes | Yes | Yes |
| 7 | HLA-DRB1\*13:02 | 1aqd (Ref) | Yes | Yes | Yes |
| 8 | HLA-DRB1\*15:01 | 1bx2 | Yes | Yes | Yes |
| 9 | HLA-DRB5\*01:01 | 1hqr | Yes | Yes | Yes |
| 10 | HLA-DRB1\*08:02 | 1aqd (Ref) | Yes | Yes | No |
| 11 | HLA-DRB1\*09:01 | 1aqd (Ref) | Yes | No | Yes |
| | | | | | |
| 12 | HLA-DRB1\*12:01 | 1aqd (Ref) | Yes | No | Yes |
| 13 | HLA-DRB4\*01:01 | 1aqd (Ref) | Yes | No | Yes |
| 14 | HLA-DQA1\*05:01/DQB1\*02:01 | 5ksu | Yes | No | Yes |
| 15 | HLA-DQA1\*03:01/DQB1\*03:02 | 5ujt | Yes | No | Yes |
| 16 | HLA-DQA1\*01:01/DQB1\*05:01 | 4grl | Yes | No | Yes |
| 17 | HLA-DQA1\*01:02/DQB1\*06:02 | 1uvq | Yes | No | Yes |
| 18 | HLA-DRB3\*01:01 | 2q6w | Yes | No | No |
| 19 | HLA-DRB3\*02:02 | 4h25 | Yes | No | No |
| 20 | HLA-DQA1\*05:01/DQB1\*03:01 | 5ksu (Ref) | Yes | No | No |
| 21 | HLA-DQA1\*04:01/DQB1\*04:02 | 4grl (Ref) | Yes | No | No |
| 22 | HLA-DPA1\*02:01/DPB1\*01:01 | 3lqz (Ref) | Yes | No | No |
| 23 | HLA-DPA1\*01:03/DPB1\*02:01 | 3lqz | Yes | No | No |
| 24 | HLA-DPA1\*01/DPB1\*04:01 | 3lqz (Ref) | Yes | No | No |
| 25 | HLA-DPA1\*03:01/DPB1\*04:02 | 3lqz (Ref) | Yes | No | No |
| 26 | HLA-DPA1\*02:01/DPB1\*05:01 | 3wex | Yes | No | No |
| 27 | HLA-DPA1\*02:01/DPB1\*14:01 | 3lqz (Ref) | Yes | No | No |
| Total | | | 27 | 10 | 16 |

## Slide 6
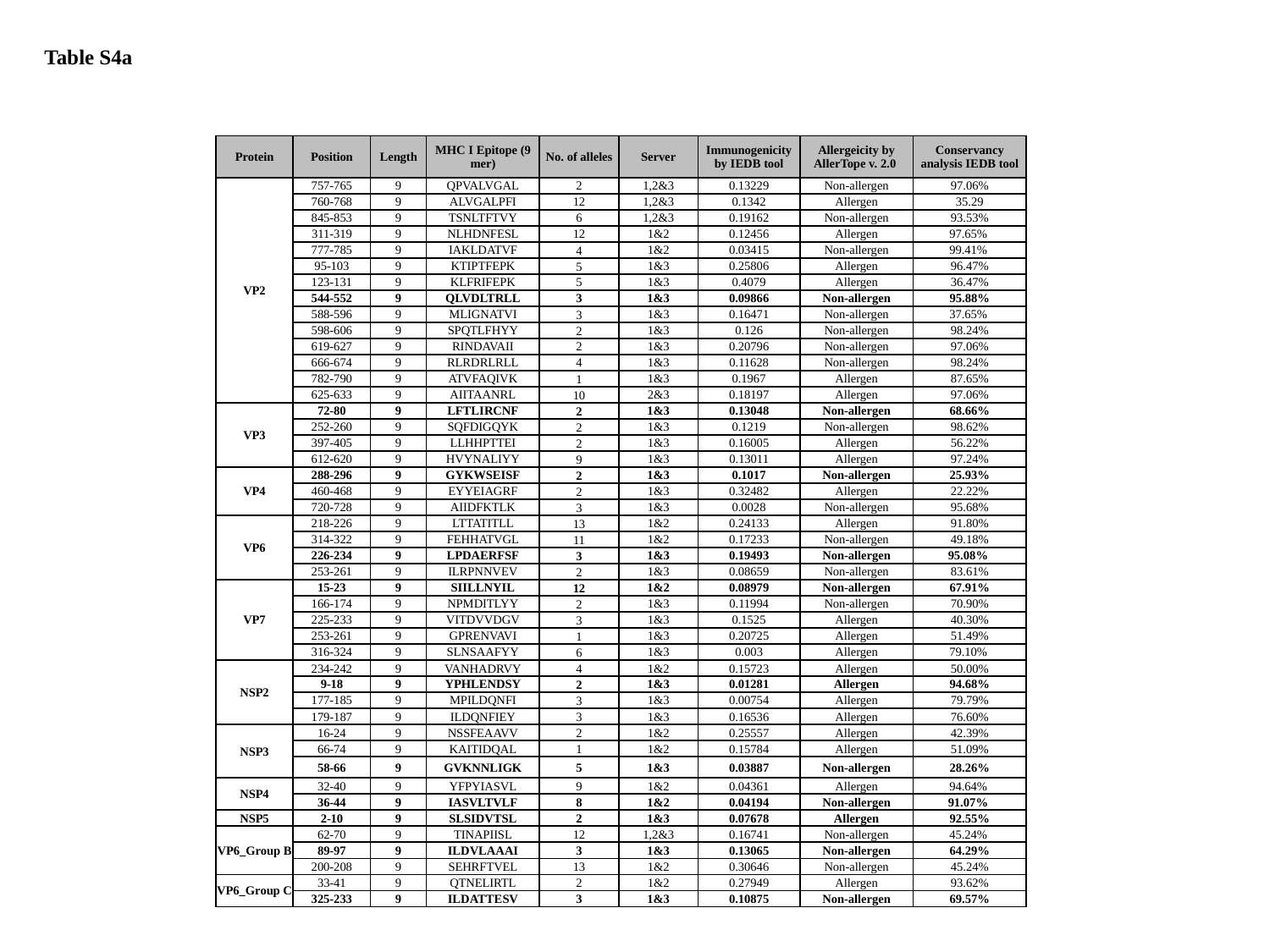

Table S4a
| Protein | Position | Length | MHC I Epitope (9 mer) | No. of alleles | Server | Immunogenicity by IEDB tool | Allergeicity by AllerTope v. 2.0 | Conservancy analysis IEDB tool |
| --- | --- | --- | --- | --- | --- | --- | --- | --- |
| VP2 | 757-765 | 9 | QPVALVGAL | 2 | 1,2&3 | 0.13229 | Non-allergen | 97.06% |
| | 760-768 | 9 | ALVGALPFI | 12 | 1,2&3 | 0.1342 | Allergen | 35.29 |
| | 845-853 | 9 | TSNLTFTVY | 6 | 1,2&3 | 0.19162 | Non-allergen | 93.53% |
| | 311-319 | 9 | NLHDNFESL | 12 | 1&2 | 0.12456 | Allergen | 97.65% |
| | 777-785 | 9 | IAKLDATVF | 4 | 1&2 | 0.03415 | Non-allergen | 99.41% |
| | 95-103 | 9 | KTIPTFEPK | 5 | 1&3 | 0.25806 | Allergen | 96.47% |
| | 123-131 | 9 | KLFRIFEPK | 5 | 1&3 | 0.4079 | Allergen | 36.47% |
| | 544-552 | 9 | QLVDLTRLL | 3 | 1&3 | 0.09866 | Non-allergen | 95.88% |
| | 588-596 | 9 | MLIGNATVI | 3 | 1&3 | 0.16471 | Non-allergen | 37.65% |
| | 598-606 | 9 | SPQTLFHYY | 2 | 1&3 | 0.126 | Non-allergen | 98.24% |
| | 619-627 | 9 | RINDAVAII | 2 | 1&3 | 0.20796 | Non-allergen | 97.06% |
| | 666-674 | 9 | RLRDRLRLL | 4 | 1&3 | 0.11628 | Non-allergen | 98.24% |
| | 782-790 | 9 | ATVFAQIVK | 1 | 1&3 | 0.1967 | Allergen | 87.65% |
| | 625-633 | 9 | AIITAANRL | 10 | 2&3 | 0.18197 | Allergen | 97.06% |
| VP3 | 72-80 | 9 | LFTLIRCNF | 2 | 1&3 | 0.13048 | Non-allergen | 68.66% |
| | 252-260 | 9 | SQFDIGQYK | 2 | 1&3 | 0.1219 | Non-allergen | 98.62% |
| | 397-405 | 9 | LLHHPTTEI | 2 | 1&3 | 0.16005 | Allergen | 56.22% |
| | 612-620 | 9 | HVYNALIYY | 9 | 1&3 | 0.13011 | Allergen | 97.24% |
| VP4 | 288-296 | 9 | GYKWSEISF | 2 | 1&3 | 0.1017 | Non-allergen | 25.93% |
| | 460-468 | 9 | EYYEIAGRF | 2 | 1&3 | 0.32482 | Allergen | 22.22% |
| | 720-728 | 9 | AIIDFKTLK | 3 | 1&3 | 0.0028 | Non-allergen | 95.68% |
| VP6 | 218-226 | 9 | LTTATITLL | 13 | 1&2 | 0.24133 | Allergen | 91.80% |
| | 314-322 | 9 | FEHHATVGL | 11 | 1&2 | 0.17233 | Non-allergen | 49.18% |
| | 226-234 | 9 | LPDAERFSF | 3 | 1&3 | 0.19493 | Non-allergen | 95.08% |
| | 253-261 | 9 | ILRPNNVEV | 2 | 1&3 | 0.08659 | Non-allergen | 83.61% |
| VP7 | 15-23 | 9 | SIILLNYIL | 12 | 1&2 | 0.08979 | Non-allergen | 67.91% |
| | 166-174 | 9 | NPMDITLYY | 2 | 1&3 | 0.11994 | Non-allergen | 70.90% |
| | 225-233 | 9 | VITDVVDGV | 3 | 1&3 | 0.1525 | Allergen | 40.30% |
| | 253-261 | 9 | GPRENVAVI | 1 | 1&3 | 0.20725 | Allergen | 51.49% |
| | 316-324 | 9 | SLNSAAFYY | 6 | 1&3 | 0.003 | Allergen | 79.10% |
| NSP2 | 234-242 | 9 | VANHADRVY | 4 | 1&2 | 0.15723 | Allergen | 50.00% |
| | 9-18 | 9 | YPHLENDSY | 2 | 1&3 | 0.01281 | Allergen | 94.68% |
| | 177-185 | 9 | MPILDQNFI | 3 | 1&3 | 0.00754 | Allergen | 79.79% |
| | 179-187 | 9 | ILDQNFIEY | 3 | 1&3 | 0.16536 | Allergen | 76.60% |
| NSP3 | 16-24 | 9 | NSSFEAAVV | 2 | 1&2 | 0.25557 | Allergen | 42.39% |
| | 66-74 | 9 | KAITIDQAL | 1 | 1&2 | 0.15784 | Allergen | 51.09% |
| | 58-66 | 9 | GVKNNLIGK | 5 | 1&3 | 0.03887 | Non-allergen | 28.26% |
| NSP4 | 32-40 | 9 | YFPYIASVL | 9 | 1&2 | 0.04361 | Allergen | 94.64% |
| | 36-44 | 9 | IASVLTVLF | 8 | 1&2 | 0.04194 | Non-allergen | 91.07% |
| NSP5 | 2-10 | 9 | SLSIDVTSL | 2 | 1&3 | 0.07678 | Allergen | 92.55% |
| VP6\_Group B | 62-70 | 9 | TINAPIISL | 12 | 1,2&3 | 0.16741 | Non-allergen | 45.24% |
| | 89-97 | 9 | ILDVLAAAI | 3 | 1&3 | 0.13065 | Non-allergen | 64.29% |
| | 200-208 | 9 | SEHRFTVEL | 13 | 1&2 | 0.30646 | Non-allergen | 45.24% |
| VP6\_Group C | 33-41 | 9 | QTNELIRTL | 2 | 1&2 | 0.27949 | Allergen | 93.62% |
| | 325-233 | 9 | ILDATTESV | 3 | 1&3 | 0.10875 | Non-allergen | 69.57% |

## Slide 7
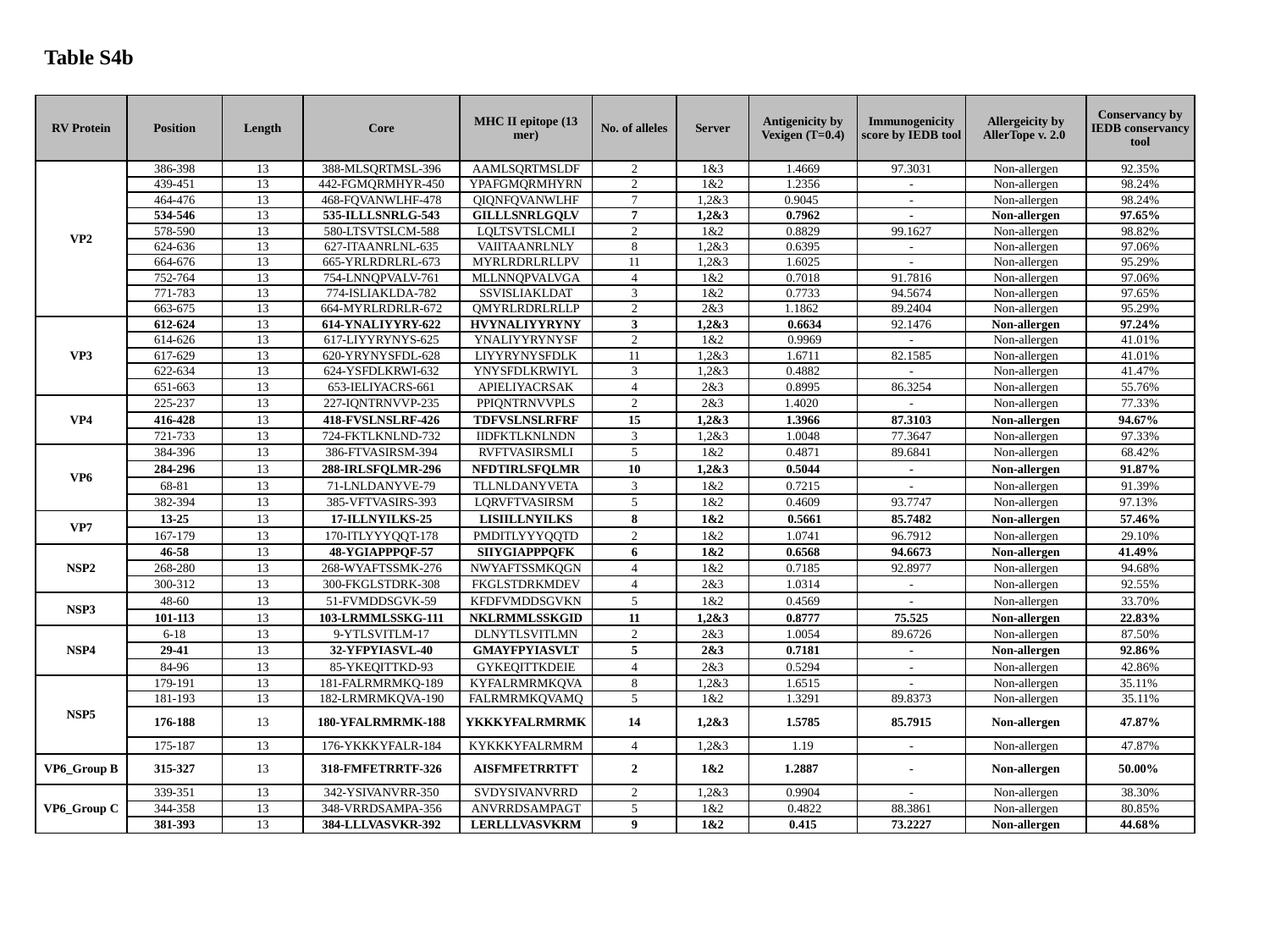

Table S4b
| RV Protein | Position | Length | Core | MHC II epitope (13 mer) | No. of alleles | Server | Antigenicity by Vexigen (T=0.4) | Immunogenicity score by IEDB tool | Allergeicity by AllerTope v. 2.0 | Conservancy by IEDB conservancy tool |
| --- | --- | --- | --- | --- | --- | --- | --- | --- | --- | --- |
| VP2 | 386-398 | 13 | 388-MLSQRTMSL-396 | AAMLSQRTMSLDF | 2 | 1&3 | 1.4669 | 97.3031 | Non-allergen | 92.35% |
| | 439-451 | 13 | 442-FGMQRMHYR-450 | YPAFGMQRMHYRN | 2 | 1&2 | 1.2356 | - | Non-allergen | 98.24% |
| | 464-476 | 13 | 468-FQVANWLHF-478 | QIQNFQVANWLHF | 7 | 1,2&3 | 0.9045 | - | Non-allergen | 98.24% |
| | 534-546 | 13 | 535-ILLLSNRLG-543 | GILLLSNRLGQLV | 7 | 1,2&3 | 0.7962 | - | Non-allergen | 97.65% |
| | 578-590 | 13 | 580-LTSVTSLCM-588 | LQLTSVTSLCMLI | 2 | 1&2 | 0.8829 | 99.1627 | Non-allergen | 98.82% |
| | 624-636 | 13 | 627-ITAANRLNL-635 | VAIITAANRLNLY | 8 | 1,2&3 | 0.6395 | - | Non-allergen | 97.06% |
| | 664-676 | 13 | 665-YRLRDRLRL-673 | MYRLRDRLRLLPV | 11 | 1,2&3 | 1.6025 | - | Non-allergen | 95.29% |
| | 752-764 | 13 | 754-LNNQPVALV-761 | MLLNNQPVALVGA | 4 | 1&2 | 0.7018 | 91.7816 | Non-allergen | 97.06% |
| | 771-783 | 13 | 774-ISLIAKLDA-782 | SSVISLIAKLDAT | 3 | 1&2 | 0.7733 | 94.5674 | Non-allergen | 97.65% |
| | 663-675 | 13 | 664-MYRLRDRLR-672 | QMYRLRDRLRLLP | 2 | 2&3 | 1.1862 | 89.2404 | Non-allergen | 95.29% |
| VP3 | 612-624 | 13 | 614-YNALIYYRY-622 | HVYNALIYYRYNY | 3 | 1,2&3 | 0.6634 | 92.1476 | Non-allergen | 97.24% |
| | 614-626 | 13 | 617-LIYYRYNYS-625 | YNALIYYRYNYSF | 2 | 1&2 | 0.9969 | - | Non-allergen | 41.01% |
| | 617-629 | 13 | 620-YRYNYSFDL-628 | LIYYRYNYSFDLK | 11 | 1,2&3 | 1.6711 | 82.1585 | Non-allergen | 41.01% |
| | 622-634 | 13 | 624-YSFDLKRWI-632 | YNYSFDLKRWIYL | 3 | 1,2&3 | 0.4882 | - | Non-allergen | 41.47% |
| | 651-663 | 13 | 653-IELIYACRS-661 | APIELIYACRSAK | 4 | 2&3 | 0.8995 | 86.3254 | Non-allergen | 55.76% |
| VP4 | 225-237 | 13 | 227-IQNTRNVVP-235 | PPIQNTRNVVPLS | 2 | 2&3 | 1.4020 | - | Non-allergen | 77.33% |
| | 416-428 | 13 | 418-FVSLNSLRF-426 | TDFVSLNSLRFRF | 15 | 1,2&3 | 1.3966 | 87.3103 | Non-allergen | 94.67% |
| | 721-733 | 13 | 724-FKTLKNLND-732 | IIDFKTLKNLNDN | 3 | 1,2&3 | 1.0048 | 77.3647 | Non-allergen | 97.33% |
| VP6 | 384-396 | 13 | 386-FTVASIRSM-394 | RVFTVASIRSMLI | 5 | 1&2 | 0.4871 | 89.6841 | Non-allergen | 68.42% |
| | 284-296 | 13 | 288-IRLSFQLMR-296 | NFDTIRLSFQLMR | 10 | 1,2&3 | 0.5044 | - | Non-allergen | 91.87% |
| | 68-81 | 13 | 71-LNLDANYVE-79 | TLLNLDANYVETA | 3 | 1&2 | 0.7215 | - | Non-allergen | 91.39% |
| | 382-394 | 13 | 385-VFTVASIRS-393 | LQRVFTVASIRSM | 5 | 1&2 | 0.4609 | 93.7747 | Non-allergen | 97.13% |
| VP7 | 13-25 | 13 | 17-ILLNYILKS-25 | LISIILLNYILKS | 8 | 1&2 | 0.5661 | 85.7482 | Non-allergen | 57.46% |
| | 167-179 | 13 | 170-ITLYYYQQT-178 | PMDITLYYYQQTD | 2 | 1&2 | 1.0741 | 96.7912 | Non-allergen | 29.10% |
| NSP2 | 46-58 | 13 | 48-YGIAPPPQF-57 | SIIYGIAPPPQFK | 6 | 1&2 | 0.6568 | 94.6673 | Non-allergen | 41.49% |
| | 268-280 | 13 | 268-WYAFTSSMK-276 | NWYAFTSSMKQGN | 4 | 1&2 | 0.7185 | 92.8977 | Non-allergen | 94.68% |
| | 300-312 | 13 | 300-FKGLSTDRK-308 | FKGLSTDRKMDEV | 4 | 2&3 | 1.0314 | - | Non-allergen | 92.55% |
| NSP3 | 48-60 | 13 | 51-FVMDDSGVK-59 | KFDFVMDDSGVKN | 5 | 1&2 | 0.4569 | - | Non-allergen | 33.70% |
| | 101-113 | 13 | 103-LRMMLSSKG-111 | NKLRMMLSSKGID | 11 | 1,2&3 | 0.8777 | 75.525 | Non-allergen | 22.83% |
| NSP4 | 6-18 | 13 | 9-YTLSVITLM-17 | DLNYTLSVITLMN | 2 | 2&3 | 1.0054 | 89.6726 | Non-allergen | 87.50% |
| | 29-41 | 13 | 32-YFPYIASVL-40 | GMAYFPYIASVLT | 5 | 2&3 | 0.7181 | - | Non-allergen | 92.86% |
| | 84-96 | 13 | 85-YKEQITTKD-93 | GYKEQITTKDEIE | 4 | 2&3 | 0.5294 | - | Non-allergen | 42.86% |
| NSP5 | 179-191 | 13 | 181-FALRMRMKQ-189 | KYFALRMRMKQVA | 8 | 1,2&3 | 1.6515 | - | Non-allergen | 35.11% |
| | 181-193 | 13 | 182-LRMRMKQVA-190 | FALRMRMKQVAMQ | 5 | 1&2 | 1.3291 | 89.8373 | Non-allergen | 35.11% |
| | 176-188 | 13 | 180-YFALRMRMK-188 | YKKKYFALRMRMK | 14 | 1,2&3 | 1.5785 | 85.7915 | Non-allergen | 47.87% |
| | 175-187 | 13 | 176-YKKKYFALR-184 | KYKKKYFALRMRM | 4 | 1,2&3 | 1.19 | - | Non-allergen | 47.87% |
| VP6\_Group B | 315-327 | 13 | 318-FMFETRRTF-326 | AISFMFETRRTFT | 2 | 1&2 | 1.2887 | - | Non-allergen | 50.00% |
| VP6\_Group C | 339-351 | 13 | 342-YSIVANVRR-350 | SVDYSIVANVRRD | 2 | 1,2&3 | 0.9904 | - | Non-allergen | 38.30% |
| | 344-358 | 13 | 348-VRRDSAMPA-356 | ANVRRDSAMPAGT | 5 | 1&2 | 0.4822 | 88.3861 | Non-allergen | 80.85% |
| | 381-393 | 13 | 384-LLLVASVKR-392 | LERLLLVASVKRM | 9 | 1&2 | 0.415 | 73.2227 | Non-allergen | 44.68% |

## Slide 8
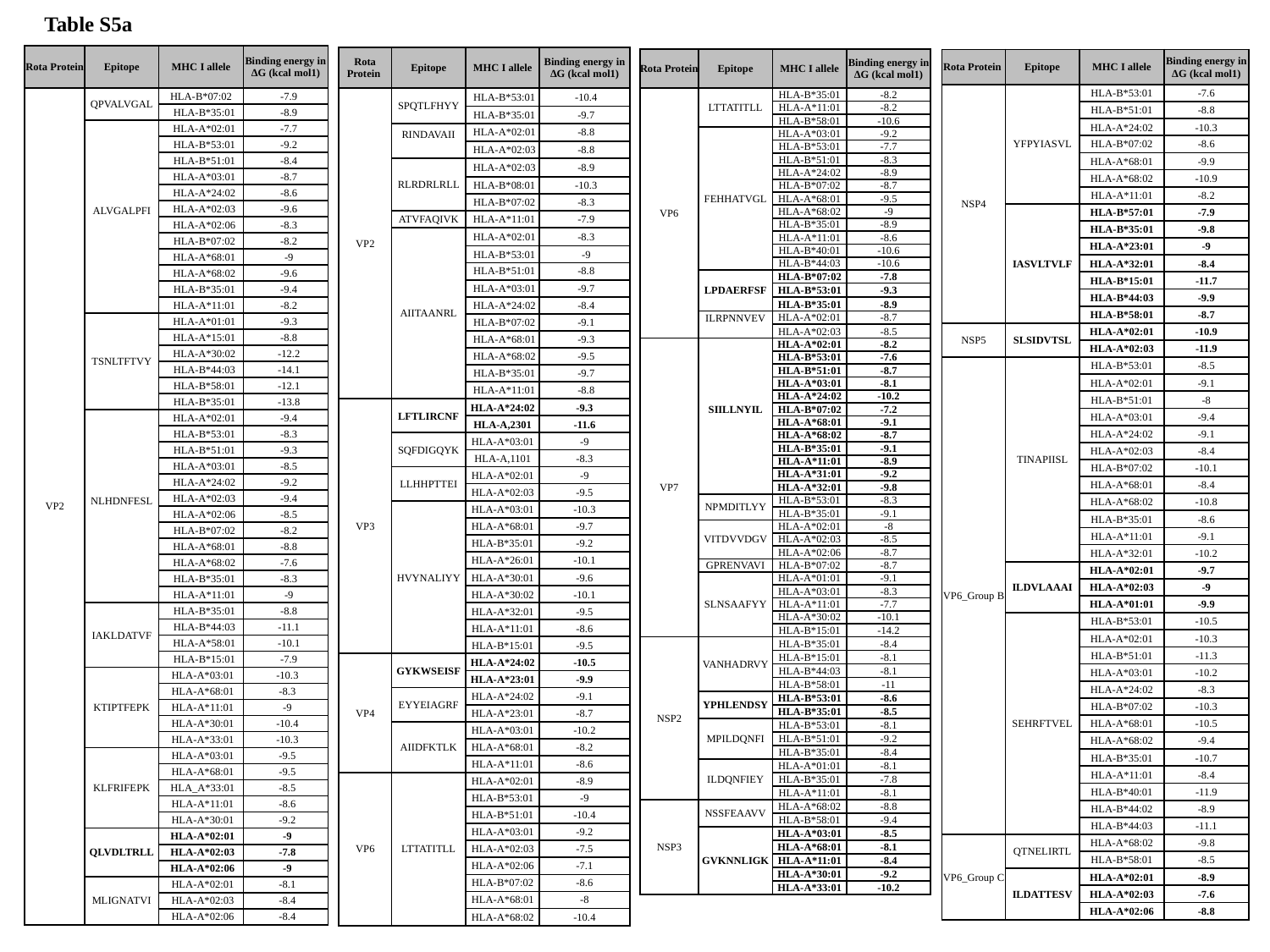

Table S5a
| Rota Protein | Epitope | MHC I allele | Binding energy in ΔG (kcal mol1) |
| --- | --- | --- | --- |
| VP2 | QPVALVGAL | HLA-B\*07:02 | -7.9 |
| | | HLA-B\*35:01 | -8.9 |
| | ALVGALPFI | HLA-A\*02:01 | -7.7 |
| | | HLA-B\*53:01 | -9.2 |
| | | HLA-B\*51:01 | -8.4 |
| | | HLA-A\*03:01 | -8.7 |
| | | HLA-A\*24:02 | -8.6 |
| | | HLA-A\*02:03 | -9.6 |
| | | HLA-A\*02:06 | -8.3 |
| | | HLA-B\*07:02 | -8.2 |
| | | HLA-A\*68:01 | -9 |
| | | HLA-A\*68:02 | -9.6 |
| | | HLA-B\*35:01 | -9.4 |
| | | HLA-A\*11:01 | -8.2 |
| | TSNLTFTVY | HLA-A\*01:01 | -9.3 |
| | | HLA-A\*15:01 | -8.8 |
| | | HLA-A\*30:02 | -12.2 |
| | | HLA-B\*44:03 | -14.1 |
| | | HLA-B\*58:01 | -12.1 |
| | | HLA-B\*35:01 | -13.8 |
| | NLHDNFESL | HLA-A\*02:01 | -9.4 |
| | | HLA-B\*53:01 | -8.3 |
| | | HLA-B\*51:01 | -9.3 |
| | | HLA-A\*03:01 | -8.5 |
| | | HLA-A\*24:02 | -9.2 |
| | | HLA-A\*02:03 | -9.4 |
| | | HLA-A\*02:06 | -8.5 |
| | | HLA-B\*07:02 | -8.2 |
| | | HLA-A\*68:01 | -8.8 |
| | | HLA-A\*68:02 | -7.6 |
| | | HLA-B\*35:01 | -8.3 |
| | | HLA-A\*11:01 | -9 |
| | IAKLDATVF | HLA-B\*35:01 | -8.8 |
| | | HLA-B\*44:03 | -11.1 |
| | | HLA-A\*58:01 | -10.1 |
| | | HLA-B\*15:01 | -7.9 |
| | KTIPTFEPK | HLA-A\*03:01 | -10.3 |
| | | HLA-A\*68:01 | -8.3 |
| | | HLA-A\*11:01 | -9 |
| | | HLA-A\*30:01 | -10.4 |
| | | HLA-A\*33:01 | -10.3 |
| | KLFRIFEPK | HLA-A\*03:01 | -9.5 |
| | | HLA-A\*68:01 | -9.5 |
| | | HLA\_A\*33:01 | -8.5 |
| | | HLA-A\*11:01 | -8.6 |
| | | HLA-A\*30:01 | -9.2 |
| | QLVDLTRLL | HLA-A\*02:01 | -9 |
| | | HLA-A\*02:03 | -7.8 |
| | | HLA-A\*02:06 | -9 |
| | MLIGNATVI | HLA-A\*02:01 | -8.1 |
| | | HLA-A\*02:03 | -8.4 |
| | | HLA-A\*02:06 | -8.4 |
| Rota Protein | Epitope | MHC I allele | Binding energy in ΔG (kcal mol1) |
| --- | --- | --- | --- |
| VP2 | SPQTLFHYY | HLA-B\*53:01 | -10.4 |
| | | HLA-B\*35:01 | -9.7 |
| | RINDAVAII | HLA-A\*02:01 | -8.8 |
| | | HLA-A\*02:03 | -8.8 |
| | RLRDRLRLL | HLA-A\*02:03 | -8.9 |
| | | HLA-B\*08:01 | -10.3 |
| | | HLA-B\*07:02 | -8.3 |
| | ATVFAQIVK | HLA-A\*11:01 | -7.9 |
| | AIITAANRL | HLA-A\*02:01 | -8.3 |
| | | HLA-B\*53:01 | -9 |
| | | HLA-B\*51:01 | -8.8 |
| | | HLA-A\*03:01 | -9.7 |
| | | HLA-A\*24:02 | -8.4 |
| | | HLA-B\*07:02 | -9.1 |
| | | HLA-A\*68:01 | -9.3 |
| | | HLA-A\*68:02 | -9.5 |
| | | HLA-B\*35:01 | -9.7 |
| | | HLA-A\*11:01 | -8.8 |
| VP3 | LFTLIRCNF | HLA-A\*24:02 | -9.3 |
| | | HLA-A,2301 | -11.6 |
| | SQFDIGQYK | HLA-A\*03:01 | -9 |
| | | HLA-A,1101 | -8.3 |
| | LLHHPTTEI | HLA-A\*02:01 | -9 |
| | | HLA-A\*02:03 | -9.5 |
| | HVYNALIYY | HLA-A\*03:01 | -10.3 |
| | | HLA-A\*68:01 | -9.7 |
| | | HLA-B\*35:01 | -9.2 |
| | | HLA-A\*26:01 | -10.1 |
| | | HLA-A\*30:01 | -9.6 |
| | | HLA-A\*30:02 | -10.1 |
| | | HLA-A\*32:01 | -9.5 |
| | | HLA-A\*11:01 | -8.6 |
| | | HLA-B\*15:01 | -9.5 |
| VP4 | GYKWSEISF | HLA-A\*24:02 | -10.5 |
| | | HLA-A\*23:01 | -9.9 |
| | EYYEIAGRF | HLA-A\*24:02 | -9.1 |
| | | HLA-A\*23:01 | -8.7 |
| | AIIDFKTLK | HLA-A\*03:01 | -10.2 |
| | | HLA-A\*68:01 | -8.2 |
| | | HLA-A\*11:01 | -8.6 |
| VP6 | LTTATITLL | HLA-A\*02:01 | -8.9 |
| | | HLA-B\*53:01 | -9 |
| | | HLA-B\*51:01 | -10.4 |
| | | HLA-A\*03:01 | -9.2 |
| | | HLA-A\*02:03 | -7.5 |
| | | HLA-A\*02:06 | -7.1 |
| | | HLA-B\*07:02 | -8.6 |
| | | HLA-A\*68:01 | -8 |
| | | HLA-A\*68:02 | -10.4 |
| Rota Protein | Epitope | MHC I allele | Binding energy in ΔG (kcal mol1) |
| --- | --- | --- | --- |
| VP6 | LTTATITLL | HLA-B\*35:01 | -8.2 |
| | | HLA-A\*11:01 | -8.2 |
| | | HLA-B\*58:01 | -10.6 |
| | FEHHATVGL | HLA-A\*03:01 | -9.2 |
| | | HLA-B\*53:01 | -7.7 |
| | | HLA-B\*51:01 | -8.3 |
| | | HLA-A\*24:02 | -8.9 |
| | | HLA-B\*07:02 | -8.7 |
| | | HLA-A\*68:01 | -9.5 |
| | | HLA-A\*68:02 | -9 |
| | | HLA-B\*35:01 | -8.9 |
| | | HLA-A\*11:01 | -8.6 |
| | | HLA-B\*40:01 | -10.6 |
| | | HLA-B\*44:03 | -10.6 |
| | LPDAERFSF | HLA-B\*07:02 | -7.8 |
| | | HLA-B\*53:01 | -9.3 |
| | | HLA-B\*35:01 | -8.9 |
| | ILRPNNVEV | HLA-A\*02:01 | -8.7 |
| | | HLA-A\*02:03 | -8.5 |
| VP7 | SIILLNYIL | HLA-A\*02:01 | -8.2 |
| | | HLA-B\*53:01 | -7.6 |
| | | HLA-B\*51:01 | -8.7 |
| | | HLA-A\*03:01 | -8.1 |
| | | HLA-A\*24:02 | -10.2 |
| | | HLA-B\*07:02 | -7.2 |
| | | HLA-A\*68:01 | -9.1 |
| | | HLA-A\*68:02 | -8.7 |
| | | HLA-B\*35:01 | -9.1 |
| | | HLA-A\*11:01 | -8.9 |
| | | HLA-A\*31:01 | -9.2 |
| | | HLA-A\*32:01 | -9.8 |
| | NPMDITLYY | HLA-B\*53:01 | -8.3 |
| | | HLA-B\*35:01 | -9.1 |
| | VITDVVDGV | HLA-A\*02:01 | -8 |
| | | HLA-A\*02:03 | -8.5 |
| | | HLA-A\*02:06 | -8.7 |
| | GPRENVAVI | HLA-B\*07:02 | -8.7 |
| | SLNSAAFYY | HLA-A\*01:01 | -9.1 |
| | | HLA-A\*03:01 | -8.3 |
| | | HLA-A\*11:01 | -7.7 |
| | | HLA-A\*30:02 | -10.1 |
| | | HLA-B\*15:01 | -14.2 |
| NSP2 | VANHADRVY | HLA-B\*35:01 | -8.4 |
| | | HLA-B\*15:01 | -8.1 |
| | | HLA-B\*44:03 | -8.1 |
| | | HLA-B\*58:01 | -11 |
| | YPHLENDSY | HLA-B\*53:01 | -8.6 |
| | | HLA-B\*35:01 | -8.5 |
| | MPILDQNFI | HLA-B\*53:01 | -8.1 |
| | | HLA-B\*51:01 | -9.2 |
| | | HLA-B\*35:01 | -8.4 |
| | ILDQNFIEY | HLA-A\*01:01 | -8.1 |
| | | HLA-B\*35:01 | -7.8 |
| | | HLA-A\*11:01 | -8.1 |
| NSP3 | NSSFEAAVV | HLA-A\*68:02 | -8.8 |
| | | HLA-B\*58:01 | -9.4 |
| | GVKNNLIGK | HLA-A\*03:01 | -8.5 |
| | | HLA-A\*68:01 | -8.1 |
| | | HLA-A\*11:01 | -8.4 |
| | | HLA-A\*30:01 | -9.2 |
| | | HLA-A\*33:01 | -10.2 |
| Rota Protein | Epitope | MHC I allele | Binding energy in ΔG (kcal mol1) |
| --- | --- | --- | --- |
| NSP4 | YFPYIASVL | HLA-B\*53:01 | -7.6 |
| | | HLA-B\*51:01 | -8.8 |
| | | HLA-A\*24:02 | -10.3 |
| | | HLA-B\*07:02 | -8.6 |
| | | HLA-A\*68:01 | -9.9 |
| | | HLA-A\*68:02 | -10.9 |
| | | HLA-A\*11:01 | -8.2 |
| | IASVLTVLF | HLA-B\*57:01 | -7.9 |
| | | HLA-B\*35:01 | -9.8 |
| | | HLA-A\*23:01 | -9 |
| | | HLA-A\*32:01 | -8.4 |
| | | HLA-B\*15:01 | -11.7 |
| | | HLA-B\*44:03 | -9.9 |
| | | HLA-B\*58:01 | -8.7 |
| NSP5 | SLSIDVTSL | HLA-A\*02:01 | -10.9 |
| | | HLA-A\*02:03 | -11.9 |
| VP6\_Group B | TINAPIISL | HLA-B\*53:01 | -8.5 |
| | | HLA-A\*02:01 | -9.1 |
| | | HLA-B\*51:01 | -8 |
| | | HLA-A\*03:01 | -9.4 |
| | | HLA-A\*24:02 | -9.1 |
| | | HLA-A\*02:03 | -8.4 |
| | | HLA-B\*07:02 | -10.1 |
| | | HLA-A\*68:01 | -8.4 |
| | | HLA-A\*68:02 | -10.8 |
| | | HLA-B\*35:01 | -8.6 |
| | | HLA-A\*11:01 | -9.1 |
| | | HLA-A\*32:01 | -10.2 |
| | ILDVLAAAI | HLA-A\*02:01 | -9.7 |
| | | HLA-A\*02:03 | -9 |
| | | HLA-A\*01:01 | -9.9 |
| | SEHRFTVEL | HLA-B\*53:01 | -10.5 |
| | | HLA-A\*02:01 | -10.3 |
| | | HLA-B\*51:01 | -11.3 |
| | | HLA-A\*03:01 | -10.2 |
| | | HLA-A\*24:02 | -8.3 |
| | | HLA-B\*07:02 | -10.3 |
| | | HLA-A\*68:01 | -10.5 |
| | | HLA-A\*68:02 | -9.4 |
| | | HLA-B\*35:01 | -10.7 |
| | | HLA-A\*11:01 | -8.4 |
| | | HLA-B\*40:01 | -11.9 |
| | | HLA-B\*44:02 | -8.9 |
| | | HLA-B\*44:03 | -11.1 |
| VP6\_Group C | QTNELIRTL | HLA-A\*68:02 | -9.8 |
| | | HLA-B\*58:01 | -8.5 |
| | ILDATTESV | HLA-A\*02:01 | -8.9 |
| | | HLA-A\*02:03 | -7.6 |
| | | HLA-A\*02:06 | -8.8 |

## Slide 9
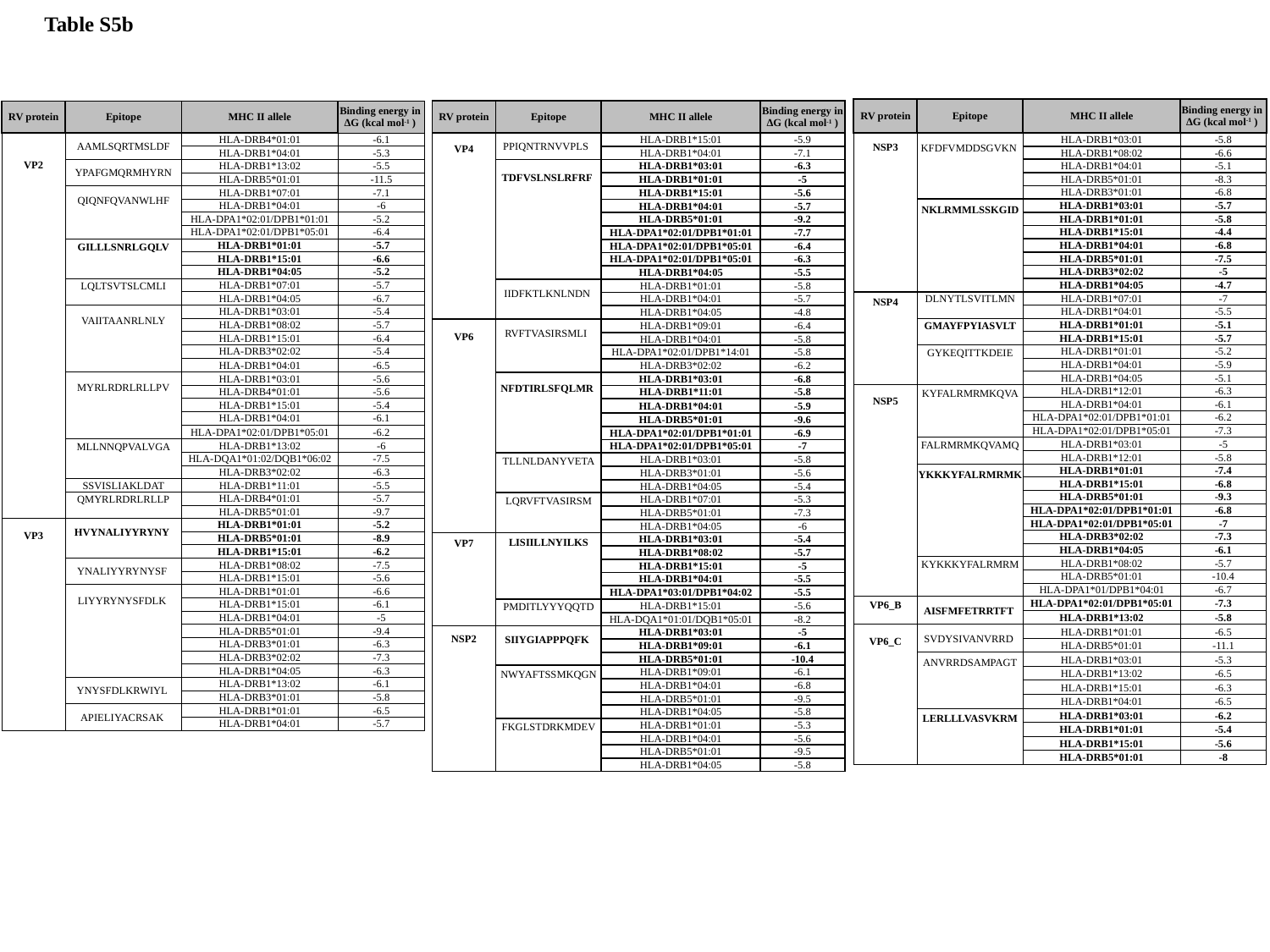

Table S5b
| RV protein | Epitope | MHC II allele | Binding energy in ΔG (kcal mol-1 ) |
| --- | --- | --- | --- |
| NSP3 | KFDFVMDDSGVKN | HLA-DRB1\*03:01 | -5.8 |
| | | HLA-DRB1\*08:02 | -6.6 |
| | | HLA-DRB1\*04:01 | -5.1 |
| | | HLA-DRB5\*01:01 | -8.3 |
| | | HLA-DRB3\*01:01 | -6.8 |
| | NKLRMMLSSKGID | HLA-DRB1\*03:01 | -5.7 |
| | | HLA-DRB1\*01:01 | -5.8 |
| | | HLA-DRB1\*15:01 | -4.4 |
| | | HLA-DRB1\*04:01 | -6.8 |
| | | HLA-DRB5\*01:01 | -7.5 |
| | | HLA-DRB3\*02:02 | -5 |
| | | HLA-DRB1\*04:05 | -4.7 |
| NSP4 | DLNYTLSVITLMN | HLA-DRB1\*07:01 | -7 |
| | | HLA-DRB1\*04:01 | -5.5 |
| | GMAYFPYIASVLT | HLA-DRB1\*01:01 | -5.1 |
| | | HLA-DRB1\*15:01 | -5.7 |
| | GYKEQITTKDEIE | HLA-DRB1\*01:01 | -5.2 |
| | | HLA-DRB1\*04:01 | -5.9 |
| | | HLA-DRB1\*04:05 | -5.1 |
| NSP5 | KYFALRMRMKQVA | HLA-DRB1\*12:01 | -6.3 |
| | | HLA-DRB1\*04:01 | -6.1 |
| | | HLA-DPA1\*02:01/DPB1\*01:01 | -6.2 |
| | | HLA-DPA1\*02:01/DPB1\*05:01 | -7.3 |
| | FALRMRMKQVAMQ | HLA-DRB1\*03:01 | -5 |
| | | HLA-DRB1\*12:01 | -5.8 |
| | YKKKYFALRMRMK | HLA-DRB1\*01:01 | -7.4 |
| | | HLA-DRB1\*15:01 | -6.8 |
| | | HLA-DRB5\*01:01 | -9.3 |
| | | HLA-DPA1\*02:01/DPB1\*01:01 | -6.8 |
| | | HLA-DPA1\*02:01/DPB1\*05:01 | -7 |
| | | HLA-DRB3\*02:02 | -7.3 |
| | | HLA-DRB1\*04:05 | -6.1 |
| | KYKKKYFALRMRM | HLA-DRB1\*08:02 | -5.7 |
| | | HLA-DRB5\*01:01 | -10.4 |
| | | HLA-DPA1\*01/DPB1\*04:01 | -6.7 |
| VP6\_B | AISFMFETRRTFT | HLA-DPA1\*02:01/DPB1\*05:01 | -7.3 |
| | | HLA-DRB1\*13:02 | -5.8 |
| VP6\_C | SVDYSIVANVRRD | HLA-DRB1\*01:01 | -6.5 |
| | | HLA-DRB5\*01:01 | -11.1 |
| | ANVRRDSAMPAGT | HLA-DRB1\*03:01 | -5.3 |
| | | HLA-DRB1\*13:02 | -6.5 |
| | | HLA-DRB1\*15:01 | -6.3 |
| | | HLA-DRB1\*04:01 | -6.5 |
| | LERLLLVASVKRM | HLA-DRB1\*03:01 | -6.2 |
| | | HLA-DRB1\*01:01 | -5.4 |
| | | HLA-DRB1\*15:01 | -5.6 |
| | | HLA-DRB5\*01:01 | -8 |
| RV protein | Epitope | MHC II allele | Binding energy in ΔG (kcal mol-1 ) |
| --- | --- | --- | --- |
| VP4 | PPIQNTRNVVPLS | HLA-DRB1\*15:01 | -5.9 |
| | | HLA-DRB1\*04:01 | -7.1 |
| | TDFVSLNSLRFRF | HLA-DRB1\*03:01 | -6.3 |
| | | HLA-DRB1\*01:01 | -5 |
| | | HLA-DRB1\*15:01 | -5.6 |
| | | HLA-DRB1\*04:01 | -5.7 |
| | | HLA-DRB5\*01:01 | -9.2 |
| | | HLA-DPA1\*02:01/DPB1\*01:01 | -7.7 |
| | | HLA-DPA1\*02:01/DPB1\*05:01 | -6.4 |
| | | HLA-DPA1\*02:01/DPB1\*05:01 | -6.3 |
| | | HLA-DRB1\*04:05 | -5.5 |
| | IIDFKTLKNLNDN | HLA-DRB1\*01:01 | -5.8 |
| | | HLA-DRB1\*04:01 | -5.7 |
| | | HLA-DRB1\*04:05 | -4.8 |
| VP6 | RVFTVASIRSMLI | HLA-DRB1\*09:01 | -6.4 |
| | | HLA-DRB1\*04:01 | -5.8 |
| | | HLA-DPA1\*02:01/DPB1\*14:01 | -5.8 |
| | | HLA-DRB3\*02:02 | -6.2 |
| | NFDTIRLSFQLMR | HLA-DRB1\*03:01 | -6.8 |
| | | HLA-DRB1\*11:01 | -5.8 |
| | | HLA-DRB1\*04:01 | -5.9 |
| | | HLA-DRB5\*01:01 | -9.6 |
| | | HLA-DPA1\*02:01/DPB1\*01:01 | -6.9 |
| | | HLA-DPA1\*02:01/DPB1\*05:01 | -7 |
| | TLLNLDANYVETA | HLA-DRB1\*03:01 | -5.8 |
| | | HLA-DRB3\*01:01 | -5.6 |
| | | HLA-DRB1\*04:05 | -5.4 |
| | LQRVFTVASIRSM | HLA-DRB1\*07:01 | -5.3 |
| | | HLA-DRB5\*01:01 | -7.3 |
| | | HLA-DRB1\*04:05 | -6 |
| VP7 | LISIILLNYILKS | HLA-DRB1\*03:01 | -5.4 |
| | | HLA-DRB1\*08:02 | -5.7 |
| | | HLA-DRB1\*15:01 | -5 |
| | | HLA-DRB1\*04:01 | -5.5 |
| | | HLA-DPA1\*03:01/DPB1\*04:02 | -5.5 |
| | PMDITLYYYQQTD | HLA-DRB1\*15:01 | -5.6 |
| | | HLA-DQA1\*01:01/DQB1\*05:01 | -8.2 |
| NSP2 | SIIYGIAPPPQFK | HLA-DRB1\*03:01 | -5 |
| | | HLA-DRB1\*09:01 | -6.1 |
| | | HLA-DRB5\*01:01 | -10.4 |
| | NWYAFTSSMKQGN | HLA-DRB1\*09:01 | -6.1 |
| | | HLA-DRB1\*04:01 | -6.8 |
| | | HLA-DRB5\*01:01 | -9.5 |
| | | HLA-DRB1\*04:05 | -5.8 |
| | FKGLSTDRKMDEV | HLA-DRB1\*01:01 | -5.3 |
| | | HLA-DRB1\*04:01 | -5.6 |
| | | HLA-DRB5\*01:01 | -9.5 |
| | | HLA-DRB1\*04:05 | -5.8 |
| RV protein | Epitope | MHC II allele | Binding energy in ΔG (kcal mol-1 ) |
| --- | --- | --- | --- |
| VP2 | AAMLSQRTMSLDF | HLA-DRB4\*01:01 | -6.1 |
| | | HLA-DRB1\*04:01 | -5.3 |
| | YPAFGMQRMHYRN | HLA-DRB1\*13:02 | -5.5 |
| | | HLA-DRB5\*01:01 | -11.5 |
| | QIQNFQVANWLHF | HLA-DRB1\*07:01 | -7.1 |
| | | HLA-DRB1\*04:01 | -6 |
| | | HLA-DPA1\*02:01/DPB1\*01:01 | -5.2 |
| | | HLA-DPA1\*02:01/DPB1\*05:01 | -6.4 |
| | GILLLSNRLGQLV | HLA-DRB1\*01:01 | -5.7 |
| | | HLA-DRB1\*15:01 | -6.6 |
| | | HLA-DRB1\*04:05 | -5.2 |
| | LQLTSVTSLCMLI | HLA-DRB1\*07:01 | -5.7 |
| | | HLA-DRB1\*04:05 | -6.7 |
| | VAIITAANRLNLY | HLA-DRB1\*03:01 | -5.4 |
| | | HLA-DRB1\*08:02 | -5.7 |
| | | HLA-DRB1\*15:01 | -6.4 |
| | | HLA-DRB3\*02:02 | -5.4 |
| | | HLA-DRB1\*04:01 | -6.5 |
| | MYRLRDRLRLLPV | HLA-DRB1\*03:01 | -5.6 |
| | | HLA-DRB4\*01:01 | -5.6 |
| | | HLA-DRB1\*15:01 | -5.4 |
| | | HLA-DRB1\*04:01 | -6.1 |
| | | HLA-DPA1\*02:01/DPB1\*05:01 | -6.2 |
| | MLLNNQPVALVGA | HLA-DRB1\*13:02 | -6 |
| | | HLA-DQA1\*01:02/DQB1\*06:02 | -7.5 |
| | | HLA-DRB3\*02:02 | -6.3 |
| | SSVISLIAKLDAT | HLA-DRB1\*11:01 | -5.5 |
| | QMYRLRDRLRLLP | HLA-DRB4\*01:01 | -5.7 |
| | | HLA-DRB5\*01:01 | -9.7 |
| VP3 | HVYNALIYYRYNY | HLA-DRB1\*01:01 | -5.2 |
| | | HLA-DRB5\*01:01 | -8.9 |
| | | HLA-DRB1\*15:01 | -6.2 |
| | YNALIYYRYNYSF | HLA-DRB1\*08:02 | -7.5 |
| | | HLA-DRB1\*15:01 | -5.6 |
| | LIYYRYNYSFDLK | HLA-DRB1\*01:01 | -6.6 |
| | | HLA-DRB1\*15:01 | -6.1 |
| | | HLA-DRB1\*04:01 | -5 |
| | | HLA-DRB5\*01:01 | -9.4 |
| | | HLA-DRB3\*01:01 | -6.3 |
| | | HLA-DRB3\*02:02 | -7.3 |
| | | HLA-DRB1\*04:05 | -6.3 |
| | YNYSFDLKRWIYL | HLA-DRB1\*13:02 | -6.1 |
| | | HLA-DRB3\*01:01 | -5.8 |
| | APIELIYACRSAK | HLA-DRB1\*01:01 | -6.5 |
| | | HLA-DRB1\*04:01 | -5.7 |

## Slide 10
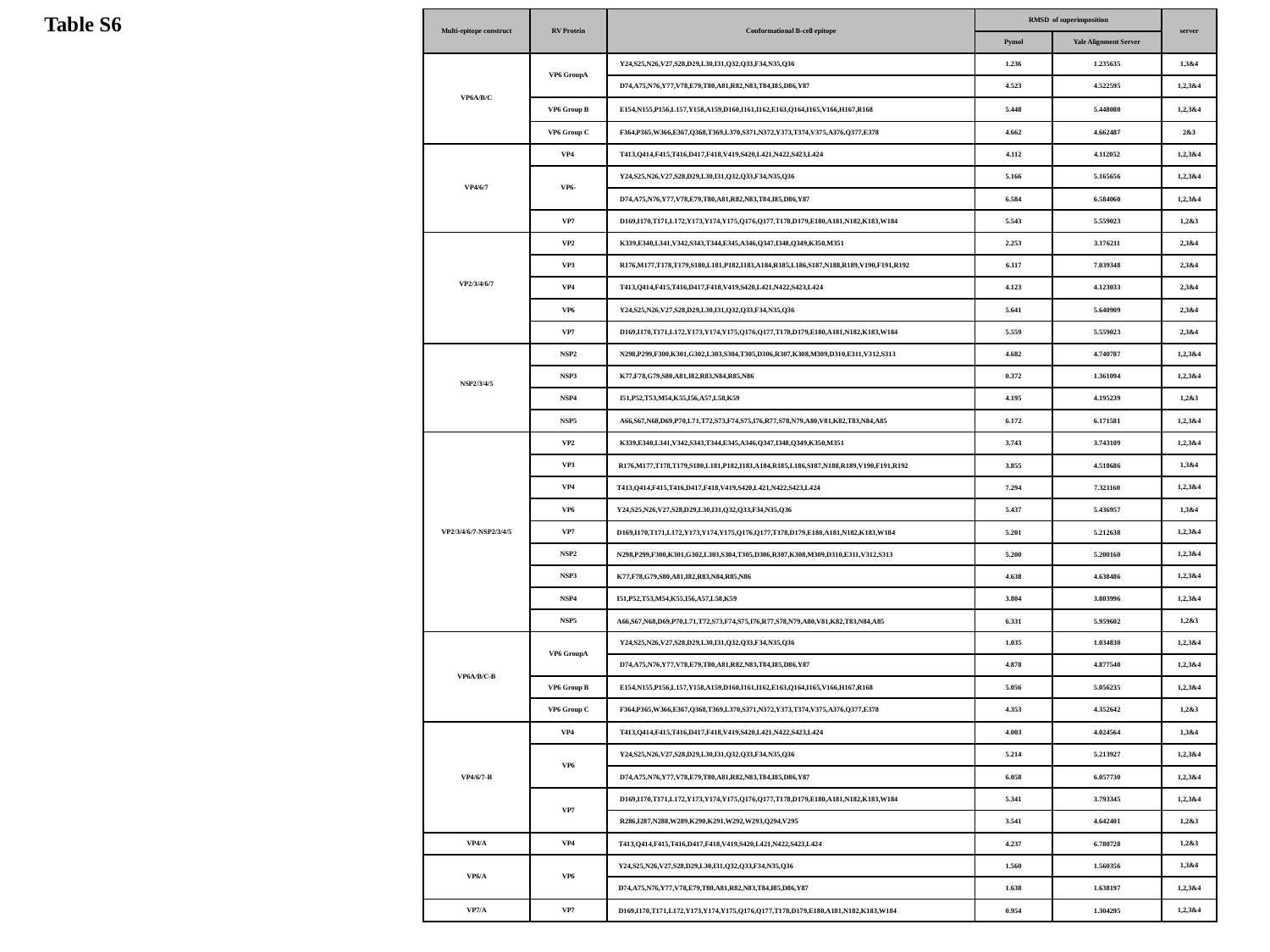

Table S6
| Multi-epitope construct | RV Protein | Conformational B-cell epitope | RMSD of superimposition | | server |
| --- | --- | --- | --- | --- | --- |
| | | | Pymol | Yale Alignment Server | |
| VP6A/B/C | VP6 GroupA | Y24,S25,N26,V27,S28,D29,L30,I31,Q32,Q33,F34,N35,Q36 | 1.236 | 1.235635 | 1,3&4 |
| | | D74,A75,N76,Y77,V78,E79,T80,A81,R82,N83,T84,I85,D86,Y87 | 4.523 | 4.522595 | 1,2,3&4 |
| | VP6 Group B | E154,N155,P156,L157,Y158,A159,D160,I161,I162,E163,Q164,I165,V166,H167,R168 | 5.448 | 5.448080 | 1,2,3&4 |
| | VP6 Group C | F364,P365,W366,E367,Q368,T369,L370,S371,N372,Y373,T374,V375,A376,Q377,E378 | 4.662 | 4.662487 | 2&3 |
| VP4/6/7 | VP4 | T413,Q414,F415,T416,D417,F418,V419,S420,L421,N422,S423,L424 | 4.112 | 4.112052 | 1,2,3&4 |
| | VP6- | Y24,S25,N26,V27,S28,D29,L30,I31,Q32,Q33,F34,N35,Q36 | 5.166 | 5.165656 | 1,2,3&4 |
| | | D74,A75,N76,Y77,V78,E79,T80,A81,R82,N83,T84,I85,D86,Y87 | 6.584 | 6.584060 | 1,2,3&4 |
| | VP7 | D169,I170,T171,L172,Y173,Y174,Y175,Q176,Q177,T178,D179,E180,A181,N182,K183,W184 | 5.543 | 5.559023 | 1,2&3 |
| VP2/3/4/6/7 | VP2 | K339,E340,L341,V342,S343,T344,E345,A346,Q347,I348,Q349,K350,M351 | 2.253 | 3.176211 | 2,3&4 |
| | VP3 | R176,M177,T178,T179,S180,L181,P182,I183,A184,R185,L186,S187,N188,R189,V190,F191,R192 | 6.117 | 7.039348 | 2,3&4 |
| | VP4 | T413,Q414,F415,T416,D417,F418,V419,S420,L421,N422,S423,L424 | 4.123 | 4.123033 | 2,3&4 |
| | VP6 | Y24,S25,N26,V27,S28,D29,L30,I31,Q32,Q33,F34,N35,Q36 | 5.641 | 5.640909 | 2,3&4 |
| | VP7 | D169,I170,T171,L172,Y173,Y174,Y175,Q176,Q177,T178,D179,E180,A181,N182,K183,W184 | 5.559 | 5.559023 | 2,3&4 |
| NSP2/3/4/5 | NSP2 | N298,P299,F300,K301,G302,L303,S304,T305,D306,R307,K308,M309,D310,E311,V312,S313 | 4.682 | 4.740787 | 1,2,3&4 |
| | NSP3 | K77,F78,G79,S80,A81,I82,R83,N84,R85,N86 | 0.372 | 1.361094 | 1,2,3&4 |
| | NSP4 | I51,P52,T53,M54,K55,I56,A57,L58,K59 | 4.195 | 4.195239 | 1,2&3 |
| | NSP5 | A66,S67,N68,D69,P70,L71,T72,S73,F74,S75,I76,R77,S78,N79,A80,V81,K82,T83,N84,A85 | 6.172 | 6.171581 | 1,2,3&4 |
| VP2/3/4/6/7-NSP2/3/4/5 | VP2 | K339,E340,L341,V342,S343,T344,E345,A346,Q347,I348,Q349,K350,M351 | 3.743 | 3.743109 | 1,2,3&4 |
| | VP3 | R176,M177,T178,T179,S180,L181,P182,I183,A184,R185,L186,S187,N188,R189,V190,F191,R192 | 3.855 | 4.518686 | 1,3&4 |
| | VP4 | T413,Q414,F415,T416,D417,F418,V419,S420,L421,N422,S423,L424 | 7.294 | 7.321160 | 1,2,3&4 |
| | VP6 | Y24,S25,N26,V27,S28,D29,L30,I31,Q32,Q33,F34,N35,Q36 | 5.437 | 5.436957 | 1,3&4 |
| | VP7 | D169,I170,T171,L172,Y173,Y174,Y175,Q176,Q177,T178,D179,E180,A181,N182,K183,W184 | 5.201 | 5.212638 | 1,2,3&4 |
| | NSP2 | N298,P299,F300,K301,G302,L303,S304,T305,D306,R307,K308,M309,D310,E311,V312,S313 | 5.200 | 5.200160 | 1,2,3&4 |
| | NSP3 | K77,F78,G79,S80,A81,I82,R83,N84,R85,N86 | 4.638 | 4.638486 | 1,2,3&4 |
| | NSP4 | I51,P52,T53,M54,K55,I56,A57,L58,K59 | 3.804 | 3.803996 | 1,2,3&4 |
| | NSP5 | A66,S67,N68,D69,P70,L71,T72,S73,F74,S75,I76,R77,S78,N79,A80,V81,K82,T83,N84,A85 | 6.331 | 5.959602 | 1,2&3 |
| VP6A/B/C-B | VP6 GroupA | Y24,S25,N26,V27,S28,D29,L30,I31,Q32,Q33,F34,N35,Q36 | 1.035 | 1.034830 | 1,2,3&4 |
| | | D74,A75,N76,Y77,V78,E79,T80,A81,R82,N83,T84,I85,D86,Y87 | 4.878 | 4.877540 | 1,2,3&4 |
| | VP6 Group B | E154,N155,P156,L157,Y158,A159,D160,I161,I162,E163,Q164,I165,V166,H167,R168 | 5.056 | 5.056235 | 1,2,3&4 |
| | VP6 Group C | F364,P365,W366,E367,Q368,T369,L370,S371,N372,Y373,T374,V375,A376,Q377,E378 | 4.353 | 4.352642 | 1,2&3 |
| VP4/6/7-B | VP4 | T413,Q414,F415,T416,D417,F418,V419,S420,L421,N422,S423,L424 | 4.003 | 4.024564 | 1,3&4 |
| | VP6 | Y24,S25,N26,V27,S28,D29,L30,I31,Q32,Q33,F34,N35,Q36 | 5.214 | 5.213927 | 1,2,3&4 |
| | | D74,A75,N76,Y77,V78,E79,T80,A81,R82,N83,T84,I85,D86,Y87 | 6.058 | 6.057730 | 1,2,3&4 |
| | VP7 | D169,I170,T171,L172,Y173,Y174,Y175,Q176,Q177,T178,D179,E180,A181,N182,K183,W184 | 5.341 | 3.793345 | 1,2,3&4 |
| | | R286,I287,N288,W289,K290,K291,W292,W293,Q294,V295 | 3.541 | 4.642401 | 1,2&3 |
| VP4/A | VP4 | T413,Q414,F415,T416,D417,F418,V419,S420,L421,N422,S423,L424 | 4.237 | 6.780728 | 1,2&3 |
| VP6/A | VP6 | Y24,S25,N26,V27,S28,D29,L30,I31,Q32,Q33,F34,N35,Q36 | 1.560 | 1.560356 | 1,3&4 |
| | | D74,A75,N76,Y77,V78,E79,T80,A81,R82,N83,T84,I85,D86,Y87 | 1.638 | 1.638197 | 1,2,3&4 |
| VP7/A | VP7 | D169,I170,T171,L172,Y173,Y174,Y175,Q176,Q177,T178,D179,E180,A181,N182,K183,W184 | 0.954 | 1.304295 | 1,2,3&4 |

## Slide 11
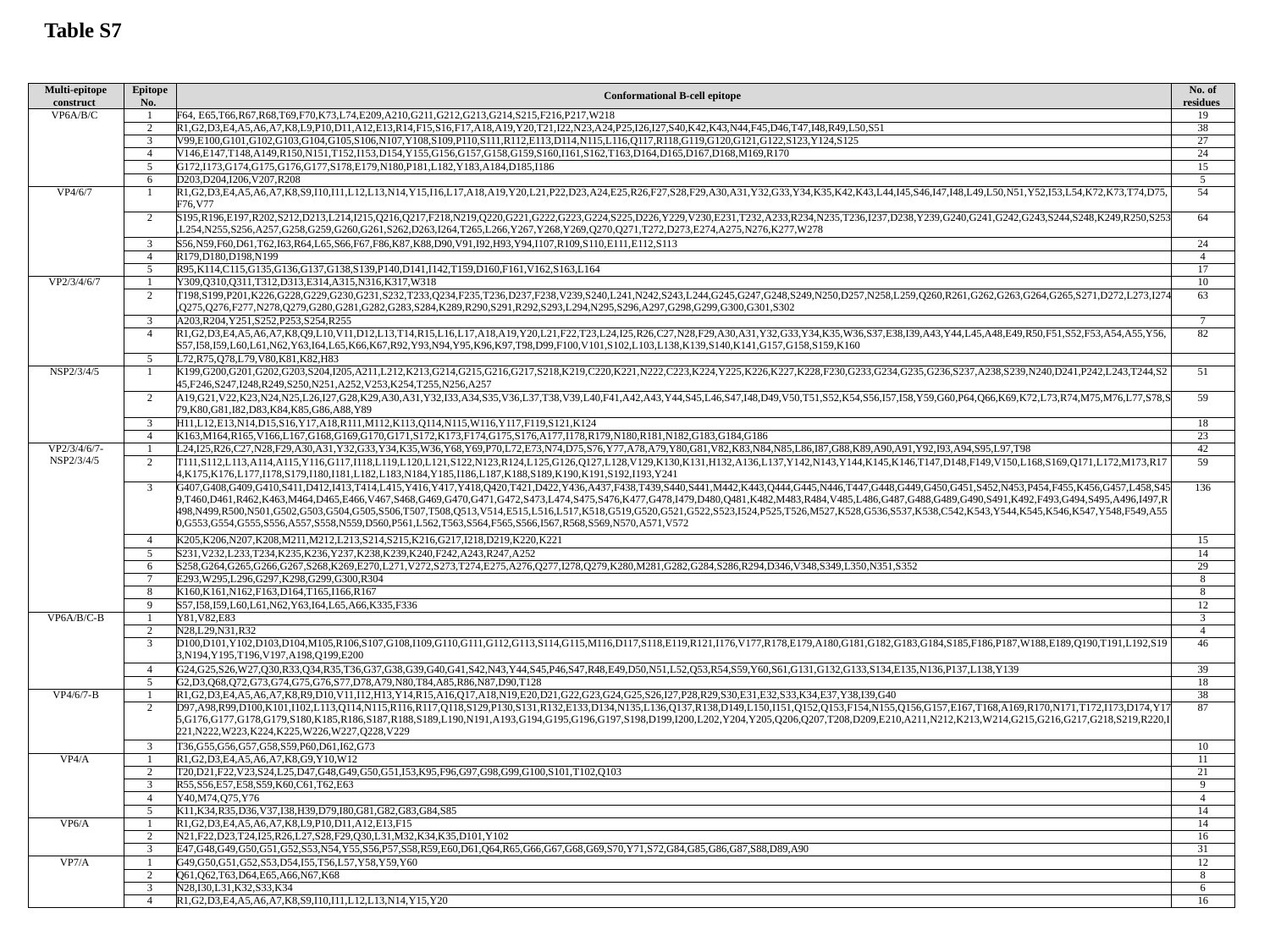

Table S7
| Multi-epitope construct | Epitope No. | Conformational B-cell epitope | No. of residues |
| --- | --- | --- | --- |
| VP6A/B/C | 1 | F64, E65,T66,R67,R68,T69,F70,K73,L74,E209,A210,G211,G212,G213,G214,S215,F216,P217,W218 | 19 |
| | 2 | R1,G2,D3,E4,A5,A6,A7,K8,L9,P10,D11,A12,E13,R14,F15,S16,F17,A18,A19,Y20,T21,I22,N23,A24,P25,I26,I27,S40,K42,K43,N44,F45,D46,T47,I48,R49,L50,S51 | 38 |
| | 3 | V99,E100,G101,G102,G103,G104,G105,S106,N107,Y108,S109,P110,S111,R112,E113,D114,N115,L116,Q117,R118,G119,G120,G121,G122,S123,Y124,S125 | 27 |
| | 4 | V146,E147,T148,A149,R150,N151,T152,I153,D154,Y155,G156,G157,G158,G159,S160,I161,S162,T163,D164,D165,D167,D168,M169,R170 | 24 |
| | 5 | G172,I173,G174,G175,G176,G177,S178,E179,N180,P181,L182,Y183,A184,D185,I186 | 15 |
| | 6 | D203,D204,I206,V207,R208 | 5 |
| VP4/6/7 | 1 | R1,G2,D3,E4,A5,A6,A7,K8,S9,I10,I11,L12,L13,N14,Y15,I16,L17,A18,A19,Y20,L21,P22,D23,A24,E25,R26,F27,S28,F29,A30,A31,Y32,G33,Y34,K35,K42,K43,L44,I45,S46,I47,I48,L49,L50,N51,Y52,I53,L54,K72,K73,T74,D75,F76,V77 | 54 |
| | 2 | S195,R196,E197,R202,S212,D213,L214,I215,Q216,Q217,F218,N219,Q220,G221,G222,G223,G224,S225,D226,Y229,V230,E231,T232,A233,R234,N235,T236,I237,D238,Y239,G240,G241,G242,G243,S244,S248,K249,R250,S253,L254,N255,S256,A257,G258,G259,G260,G261,S262,D263,I264,T265,L266,Y267,Y268,Y269,Q270,Q271,T272,D273,E274,A275,N276,K277,W278 | 64 |
| | 3 | S56,N59,F60,D61,T62,I63,R64,L65,S66,F67,F86,K87,K88,D90,V91,I92,H93,Y94,I107,R109,S110,E111,E112,S113 | 24 |
| | 4 | R179,D180,D198,N199 | 4 |
| | 5 | R95,K114,C115,G135,G136,G137,G138,S139,P140,D141,I142,T159,D160,F161,V162,S163,L164 | 17 |
| VP2/3/4/6/7 | 1 | Y309,Q310,Q311,T312,D313,E314,A315,N316,K317,W318 | 10 |
| | 2 | T198,S199,P201,K226,G228,G229,G230,G231,S232,T233,Q234,F235,T236,D237,F238,V239,S240,L241,N242,S243,L244,G245,G247,G248,S249,N250,D257,N258,L259,Q260,R261,G262,G263,G264,G265,S271,D272,L273,I274,Q275,Q276,F277,N278,Q279,G280,G281,G282,G283,S284,K289,R290,S291,R292,S293,L294,N295,S296,A297,G298,G299,G300,G301,S302 | 63 |
| | 3 | A203,R204,Y251,S252,P253,S254,R255 | 7 |
| | 4 | R1,G2,D3,E4,A5,A6,A7,K8,Q9,L10,V11,D12,L13,T14,R15,L16,L17,A18,A19,Y20,L21,F22,T23,L24,I25,R26,C27,N28,F29,A30,A31,Y32,G33,Y34,K35,W36,S37,E38,I39,A43,Y44,L45,A48,E49,R50,F51,S52,F53,A54,A55,Y56,S57,I58,I59,L60,L61,N62,Y63,I64,L65,K66,K67,R92,Y93,N94,Y95,K96,K97,T98,D99,F100,V101,S102,L103,L138,K139,S140,K141,G157,G158,S159,K160 | 82 |
| | 5 | L72,R75,Q78,L79,V80,K81,K82,H83 | |
| NSP2/3/4/5 | 1 | K199,G200,G201,G202,G203,S204,I205,A211,L212,K213,G214,G215,G216,G217,S218,K219,C220,K221,N222,C223,K224,Y225,K226,K227,K228,F230,G233,G234,G235,G236,S237,A238,S239,N240,D241,P242,L243,T244,S245,F246,S247,I248,R249,S250,N251,A252,V253,K254,T255,N256,A257 | 51 |
| | 2 | A19,G21,V22,K23,N24,N25,L26,I27,G28,K29,A30,A31,Y32,I33,A34,S35,V36,L37,T38,V39,L40,F41,A42,A43,Y44,S45,L46,S47,I48,D49,V50,T51,S52,K54,S56,I57,I58,Y59,G60,P64,Q66,K69,K72,L73,R74,M75,M76,L77,S78,S79,K80,G81,I82,D83,K84,K85,G86,A88,Y89 | 59 |
| | 3 | H11,L12,E13,N14,D15,S16,Y17,A18,R111,M112,K113,Q114,N115,W116,Y117,F119,S121,K124 | 18 |
| | 4 | K163,M164,R165,V166,L167,G168,G169,G170,G171,S172,K173,F174,G175,S176,A177,I178,R179,N180,R181,N182,G183,G184,G186 | 23 |
| VP2/3/4/6/7-NSP2/3/4/5 | 1 | L24,I25,R26,C27,N28,F29,A30,A31,Y32,G33,Y34,K35,W36,Y68,Y69,P70,L72,E73,N74,D75,S76,Y77,A78,A79,Y80,G81,V82,K83,N84,N85,L86,I87,G88,K89,A90,A91,Y92,I93,A94,S95,L97,T98 | 42 |
| | 2 | T111,S112,L113,A114,A115,Y116,G117,I118,L119,L120,L121,S122,N123,R124,L125,G126,Q127,L128,V129,K130,K131,H132,A136,L137,Y142,N143,Y144,K145,K146,T147,D148,F149,V150,L168,S169,Q171,L172,M173,R174,K175,K176,L177,I178,S179,I180,I181,L182,L183,N184,Y185,I186,L187,K188,S189,K190,K191,S192,I193,Y241 | 59 |
| | 3 | G407,G408,G409,G410,S411,D412,I413,T414,L415,Y416,Y417,Y418,Q420,T421,D422,Y436,A437,F438,T439,S440,S441,M442,K443,Q444,G445,N446,T447,G448,G449,G450,G451,S452,N453,P454,F455,K456,G457,L458,S459,T460,D461,R462,K463,M464,D465,E466,V467,S468,G469,G470,G471,G472,S473,L474,S475,S476,K477,G478,I479,D480,Q481,K482,M483,R484,V485,L486,G487,G488,G489,G490,S491,K492,F493,G494,S495,A496,I497,R498,N499,R500,N501,G502,G503,G504,G505,S506,T507,T508,Q513,V514,E515,L516,L517,K518,G519,G520,G521,G522,S523,I524,P525,T526,M527,K528,G536,S537,K538,C542,K543,Y544,K545,K546,K547,Y548,F549,A550,G553,G554,G555,S556,A557,S558,N559,D560,P561,L562,T563,S564,F565,S566,I567,R568,S569,N570,A571,V572 | 136 |
| | 4 | K205,K206,N207,K208,M211,M212,L213,S214,S215,K216,G217,I218,D219,K220,K221 | 15 |
| | 5 | S231,V232,L233,T234,K235,K236,Y237,K238,K239,K240,F242,A243,R247,A252 | 14 |
| | 6 | S258,G264,G265,G266,G267,S268,K269,E270,L271,V272,S273,T274,E275,A276,Q277,I278,Q279,K280,M281,G282,G284,S286,R294,D346,V348,S349,L350,N351,S352 | 29 |
| | 7 | E293,W295,L296,G297,K298,G299,G300,R304 | 8 |
| | 8 | K160,K161,N162,F163,D164,T165,I166,R167 | 8 |
| | 9 | S57,I58,I59,L60,L61,N62,Y63,I64,L65,A66,K335,F336 | 12 |
| VP6A/B/C-B | 1 | Y81,V82,E83 | 3 |
| | 2 | N28,L29,N31,R32 | 4 |
| | 3 | D100,D101,Y102,D103,D104,M105,R106,S107,G108,I109,G110,G111,G112,G113,S114,G115,M116,D117,S118,E119,R121,I176,V177,R178,E179,A180,G181,G182,G183,G184,S185,F186,P187,W188,E189,Q190,T191,L192,S193,N194,Y195,T196,V197,A198,Q199,E200 | 46 |
| | 4 | G24,G25,S26,W27,Q30,R33,Q34,R35,T36,G37,G38,G39,G40,G41,S42,N43,Y44,S45,P46,S47,R48,E49,D50,N51,L52,Q53,R54,S59,Y60,S61,G131,G132,G133,S134,E135,N136,P137,L138,Y139 | 39 |
| | 5 | G2,D3,Q68,Q72,G73,G74,G75,G76,S77,D78,A79,N80,T84,A85,R86,N87,D90,T128 | 18 |
| VP4/6/7-B | 1 | R1,G2,D3,E4,A5,A6,A7,K8,R9,D10,V11,I12,H13,Y14,R15,A16,Q17,A18,N19,E20,D21,G22,G23,G24,G25,S26,I27,P28,R29,S30,E31,E32,S33,K34,E37,Y38,I39,G40 | 38 |
| | 2 | D97,A98,R99,D100,K101,I102,L113,Q114,N115,R116,R117,Q118,S129,P130,S131,R132,E133,D134,N135,L136,Q137,R138,D149,L150,I151,Q152,Q153,F154,N155,Q156,G157,E167,T168,A169,R170,N171,T172,I173,D174,Y175,G176,G177,G178,G179,S180,K185,R186,S187,R188,S189,L190,N191,A193,G194,G195,G196,G197,S198,D199,I200,L202,Y204,Y205,Q206,Q207,T208,D209,E210,A211,N212,K213,W214,G215,G216,G217,G218,S219,R220,I221,N222,W223,K224,K225,W226,W227,Q228,V229 | 87 |
| | 3 | T36,G55,G56,G57,G58,S59,P60,D61,I62,G73 | 10 |
| VP4/A | 1 | R1,G2,D3,E4,A5,A6,A7,K8,G9,Y10,W12 | 11 |
| | 2 | T20,D21,F22,V23,S24,L25,D47,G48,G49,G50,G51,I53,K95,F96,G97,G98,G99,G100,S101,T102,Q103 | 21 |
| | 3 | R55,S56,E57,E58,S59,K60,C61,T62,E63 | 9 |
| | 4 | Y40,M74,Q75,Y76 | 4 |
| | 5 | K11,K34,R35,D36,V37,I38,H39,D79,I80,G81,G82,G83,G84,S85 | 14 |
| VP6/A | 1 | R1,G2,D3,E4,A5,A6,A7,K8,L9,P10,D11,A12,E13,F15 | 14 |
| | 2 | N21,F22,D23,T24,I25,R26,L27,S28,F29,Q30,L31,M32,K34,K35,D101,Y102 | 16 |
| | 3 | E47,G48,G49,G50,G51,G52,S53,N54,Y55,S56,P57,S58,R59,E60,D61,Q64,R65,G66,G67,G68,G69,S70,Y71,S72,G84,G85,G86,G87,S88,D89,A90 | 31 |
| VP7/A | 1 | G49,G50,G51,G52,S53,D54,I55,T56,L57,Y58,Y59,Y60 | 12 |
| | 2 | Q61,Q62,T63,D64,E65,A66,N67,K68 | 8 |
| | 3 | N28,I30,L31,K32,S33,K34 | 6 |
| | 4 | R1,G2,D3,E4,A5,A6,A7,K8,S9,I10,I11,L12,L13,N14,Y15,Y20 | 16 |

## Slide 12
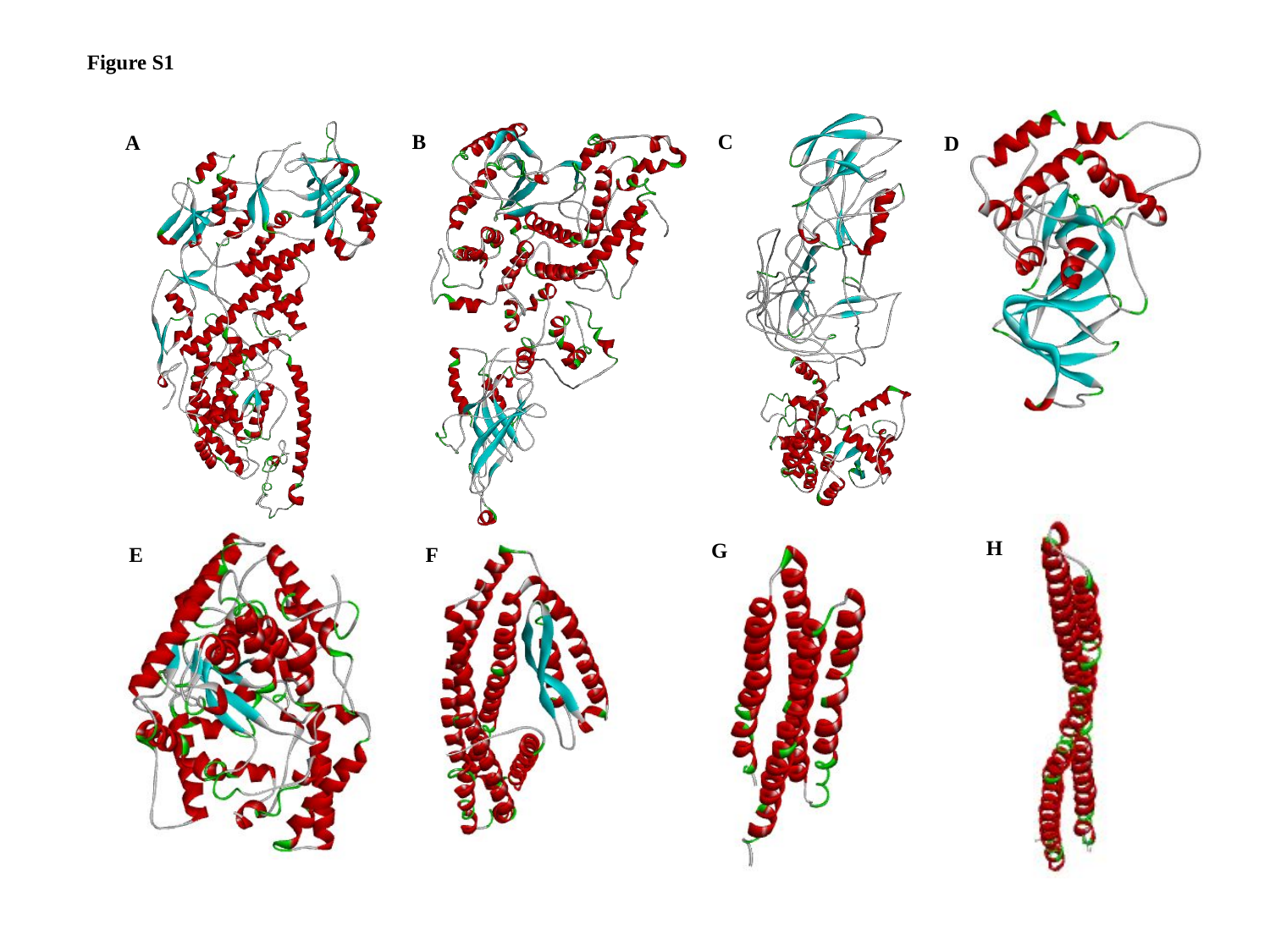

Figure S1
B
C
A
D
H
G
F
E

## Slide 13
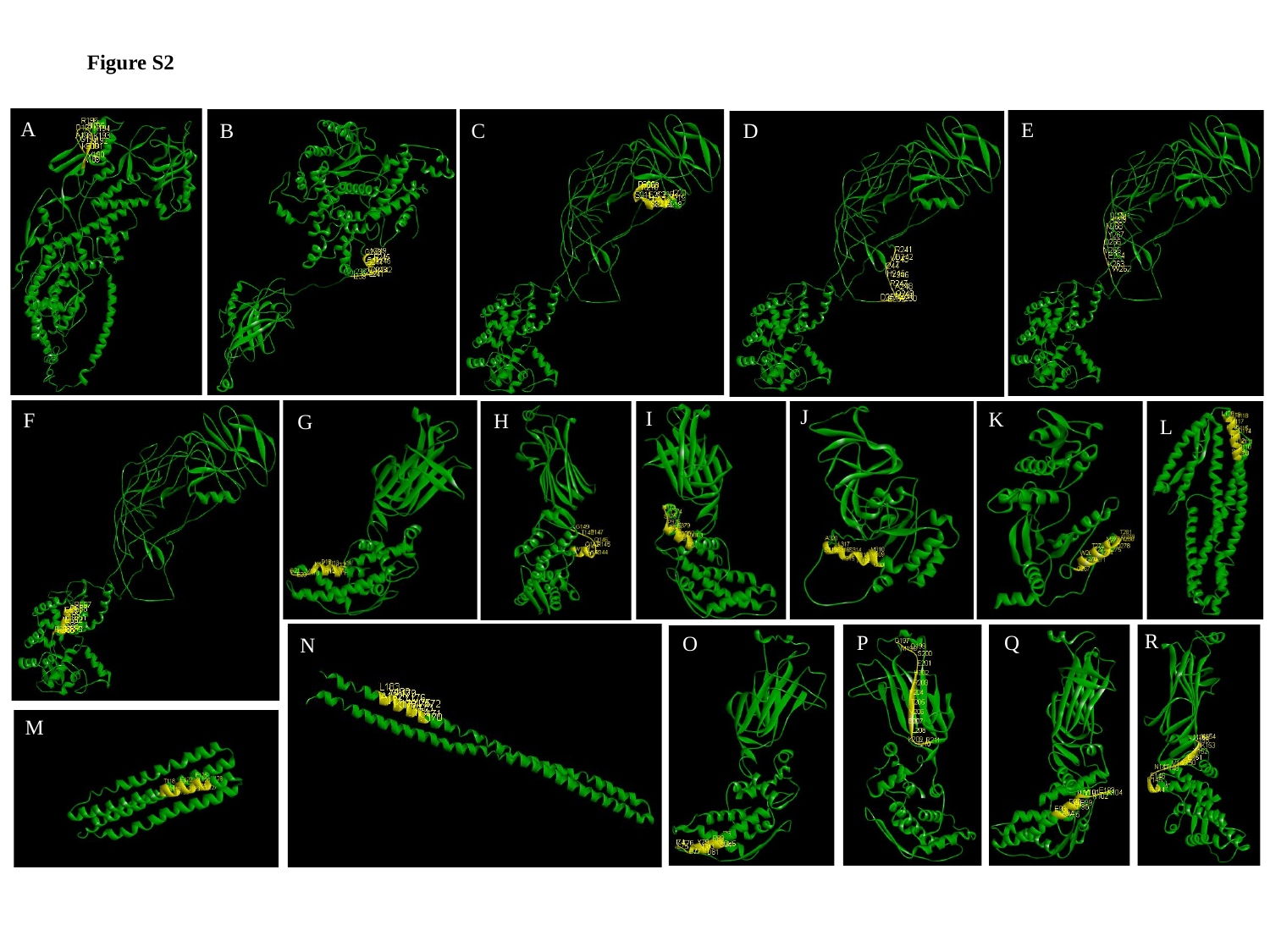

Figure S2
A
B
C
E
D
J
I
K
F
H
G
L
R
P
Q
O
N
M

## Slide 14
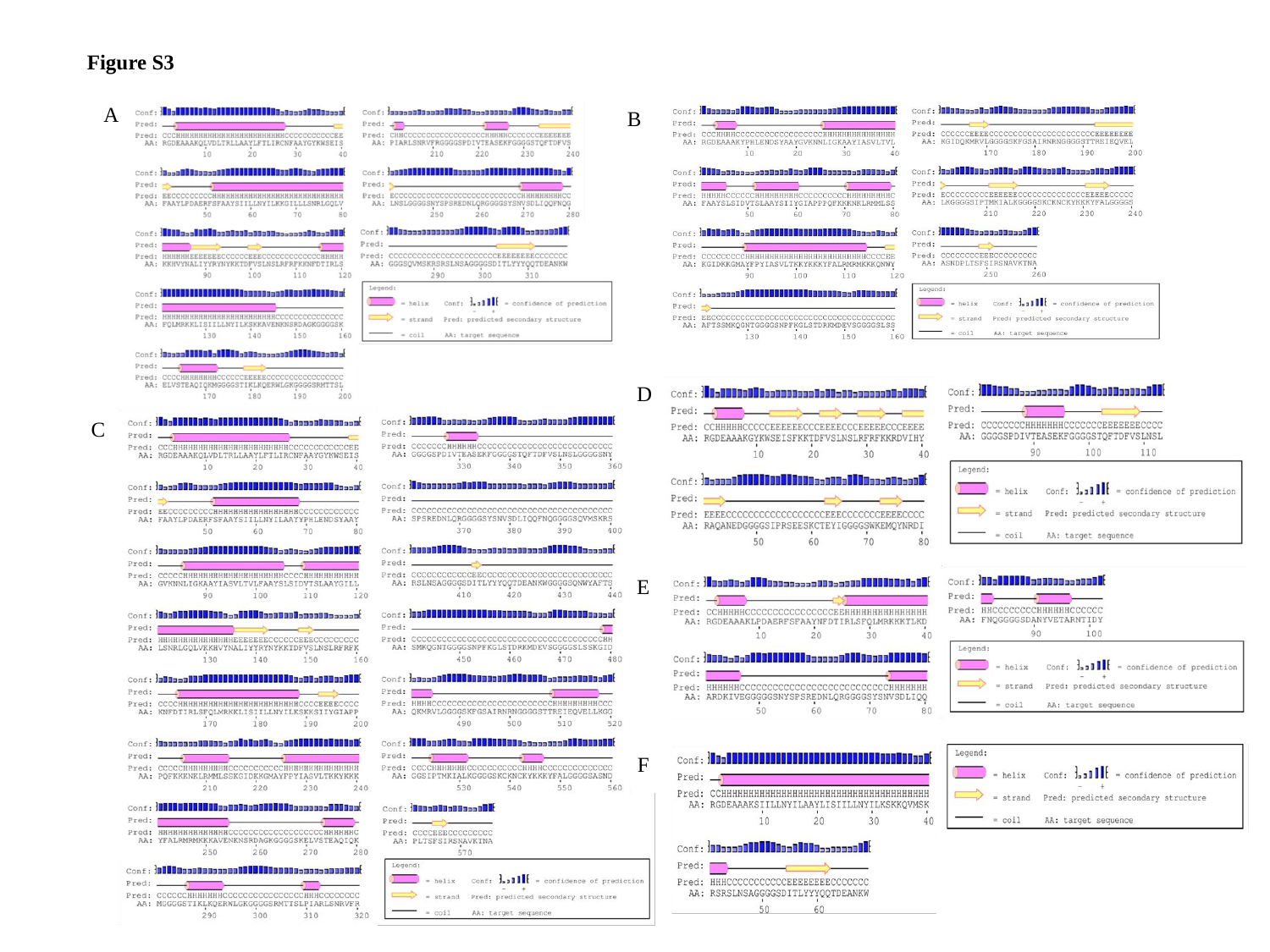

Figure S3
A
B
D
C
E
F

## Slide 15
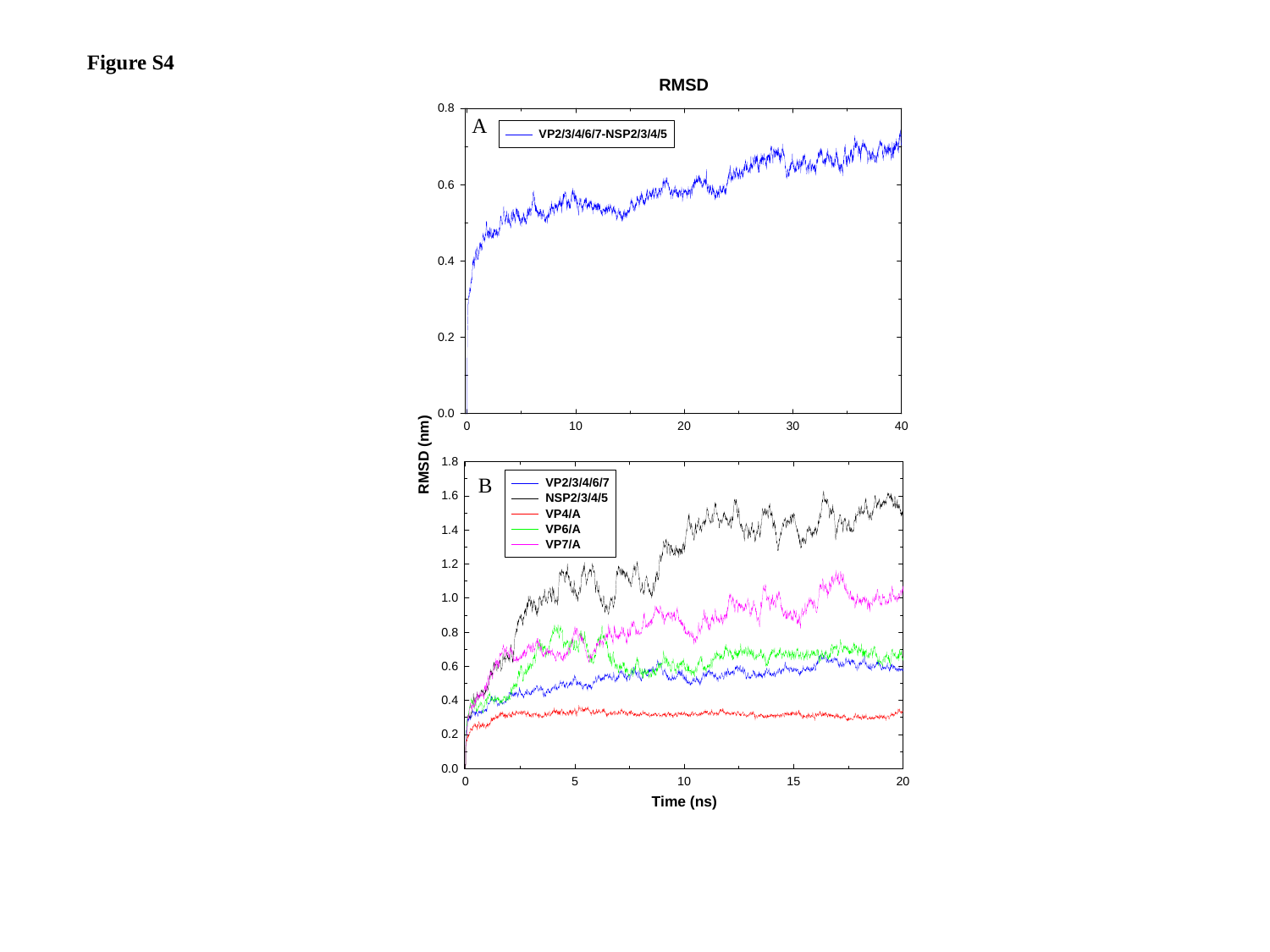

Figure S4
A
B

## Slide 16
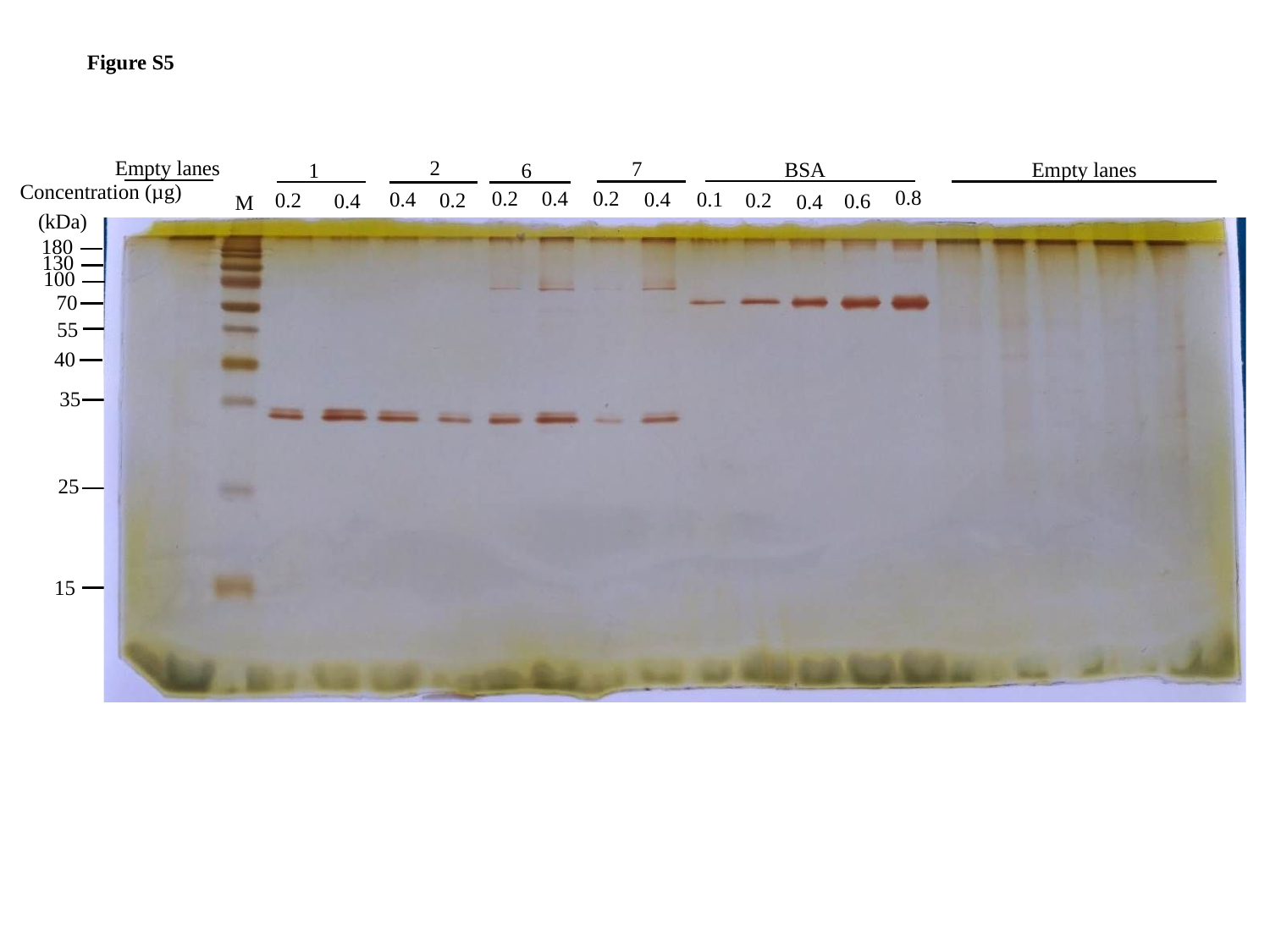

Figure S5
2
Empty lanes
7
BSA
Empty lanes
1
6
Concentration (µg)
0.8
0.2
0.4
0.2
0.4
0.1
0.4
0.2
0.2
0.2
0.6
0.4
0.4
M
(kDa)
180
130
100
70
55
40
35
25
15
